# Seeds of Hope: Cross-National Analysis of Childhood Predictors of Hope in 22 Countries

**DOI:** 10.1007/s11482-025-10450-0

**Published:** 2025-05-19

**Authors:** Victor Counted, Katelyn N. G. Long, Richard G. Cowden, Charlotte V. O. Witvliet, Cristina Gibson, Alicia Cortright, James Walsh, Emily Purcell, Fernando Garzon, William Hathaway, Byron R. Johnson, Tyler J. VanderWeele

**Affiliations:** 1 College of Health and Behavioral Sciences, Regent University, Virginia Beach, VA 23464 USA; 2 The Human Flourishing Program, Harvard University, Cambridge, MA USA; 3 Institute for Studies of Religion, Baylor University, Waco, TX USA; 4 Graziadio Business School, Pepperdine University, Los Angeles County, CA USA; 5 The Agency Fund, Berkeley, CA USA; 6 The Meros Center, Wisconsin Lutheran College, Wauwatosa, WI USA; 7 Psychology Department, Belmont University, Nashville, TN USA; 8 Department of Epidemiology, Harvard T.H. Chan School of Public Health, Boston, MA USA

**Keywords:** Hope, Cross-Cultural, Flourishing, Global Flourishing Study, Psychological Well-being, Self-Rated Hope, Spatial Hope in Adulthood

## Abstract

This study investigates childhood predictors of adult hope across 22 countries, leveraging data from 202,898 participants in the Global Flourishing Study. Key findings indicate that positive childhood experiences, such as excellent or very good health, supportive parental relationships, and regular religious attendance, are strongly associated with higher levels of hope in adulthood. Conversely, negative experiences like abuse and feeling like an outsider during childhood are linked to lower levels of hope. In many countries, excellent self-rated health during childhood emerged as a strong predictor of future hope (β = 0.48, 95% CI: 0.31, 0.65), whereas experiences of abuse correlated with lower hope levels (β = -0.16, 95% CI: -0.22, -0.10). The association between attending religious services during childhood and future hope showed notable variation in effect sizes across countries, with weekly childhood attendance in Sweden demonstrating a significant increase in hope (β = 0.36, 95% CI: 0.17, 0.54) on weekly attendance, while Indonesia also showed a significant but smaller increase (β = 0.18, 95% CI: -0.04, 0.40), with Argentina showing essentially no association (β = 0.03, 95% CI: -0.13, 0.20). The impact of parental relationships varied, with notable differences observed in countries like Germany and India. These associations were robust to potential confounding, as indicated by sensitivity analyses using E-values. This cross-national study underscores the potential role of early life experiences in shaping hope, offering valuable benchmarks for future research and interventions aimed at fostering hope from a young age in diverse cultural contexts.

**Supplementary Information:**

The online version contains supplementary material available at 10.1007/s11482-025-10450-0.

## Introduction

Hope is not merely a disposition for pursuing a good vision for the future but a complex psychological construct that energizes individuals to navigate through adversity. It involves resisting the passivity of both presumption and despair to bolster resilience and recovery from difficult situations (Counted et al., 2022; Witvliet et al., 2022). The psychological science of hope has been shaped substantially by the seminal contributions of Snyder and colleagues (1991), who conceptualized hope as a cognitive process underpinned by 'pathways'—the perceived capacity to devise alternate routes to desired goals—and 'agency'—the individual motivational drive and capacity to pursue these goals. This dual-component model of hope has shaped the discourse in positive psychology, emphasizing the role of hope in navigating life’s challenges and personal growth and establishing ties to a spectrum of positive psychological outcomes, including improved mental health (Long et al., 2024), self-efficacy (Magaletta & Oliver, 1999), enhanced stress management capabilities (Alarcon et al., 2013; Gallagher & Lopez, 2017), and elevated life satisfaction (Bailey et al., 2007).

Many studies within Western societies (McGrath, 2015; Park et al., 2006) that emphasize individual pathways and agency to reach goals (Magaletta & Oliver, 1999; Snyder et al., 1991) have largely overlooked other non-Western societies where collective well-being and shared goals grounded in communal values are important (Thomas et al., 2023, 2025). Several studies have tried to establish whether the two-factor structure of hope (i.e., agency and pathways) for Snyder’s psychometric assessment is applicable across countries. While findings from China, France, Japan, and Portugal have supported this bifurcated model, evidence from analyses with samples from Brazil, Spain, and the Netherlands support a one-factor solution (Edwards & McConnell, 2023). Scholars have also investigated the structure of the Children's Hope Scale (Snyder et al., 1997), with some finding that a one-factor model for the measure of hope may be a better fit for multiple cross-cultural samples (Edwards & McConnell, 2023).

Western-centric bias is evident in the application of hope interventions that may not fully resonate in non-Western settings. For instance, hope interventions focused on individual goal-setting might not be as effective in societies that value collective achievements over personal aspirations, as seen in some East Asian or African communities (Bernardo et al., 2018; Hendriks et al., 2018). Within a South African context, research with community-nominated hope exemplars revealed that many goals arose in adversity and focused on a vision of the common good to benefit others, often far into the future, with pathways that often involved relationships and spirituality (Thomas et al., 2023, 2025).

Existing research predominantly focuses on the manifestations and outcomes of hope in adult populations, largely overlooking the influences of childhood experiences that precede these adult manifestations (Bailey et al., 2007). The importance of studying hope's developmental trajectory becomes clear when considering the global applicability of positive psychological theories and interventions. Adjusting hope interventions (e.g., hope therapy) to account for cultural and contextual nuances, as found to benefit South African trauma survivors (Joubert & Guse, 2022) and juvenile offenders (Marsay et al., 2018), highlights the profound impact of culture and context on the effectiveness of positive psychological support. This underscores the importance of how a diverse ecological system within a particular country might influence how specific childhood factors shape hope. For example, in cultures that prioritize communal identity over individual identity, we might expect the association between quality of relationship with parents to have a stronger role in shaping hope. Cross-national understanding of childhood factors linked to hope in adulthood could support the development of more universally applicable and nationally resonant interventions to promote hope. Exploring how hope is cultivated, especially how it is influenced by childhood experiences across diverse cultural backgrounds, remains underexplored. The gap is particularly pronounced in cross-cultural research, where the influence of diverse childhood experiences on the cultivation of hope is not fully understood. This oversight in literature not only constrains the holistic understanding of the childhood experiences that shape hope but also limits the development of culturally sensitive interventions designed to nurture hope across diverse populations.

Aside from the value of cross-cultural perspectives, understanding childhood antecedents of adult hope can inform the development of early educational and health interventions aimed at fostering resilience, a positive outlook, and a range of other health and well-being outcomes for future generations. For instance, the year a person is born can introduce generational influence, where historical and socio-economic conditions prevailing during childhood could influence one’s outlook on life. An example of this comes from research that suggests children who grow up during economic recessions may develop different levels of hope compared to those raised in prosperity (Elder, 1998), which in turn may affect other positive outcomes (e.g., feeling good, happiness, optimism about the future) in adulthood (Long et al., 2024). Gender roles and societal expectations might further shape hopeful aspirations, as these factors could determine the resources and support available for coping with adversity (Eccles, 1994). Family structure and the quality of parental relationships are also pivotal, with studies suggesting that secure and supportive relationships with parents are foundational for nurturing hope (Bowlby, 2008; Jiang et al., 2013; Shorey et al., 2003). This hope mediates the attachment-health link (Jiang et al., 2013), fostering a sense of possibility and agency that persists into adulthood. Religious service attendance and affiliation can provide a thick community and a framework for understanding life's challenges, offering hope through faith and collective support (Koenig, 2013; Pawlikoswku et al., 2019), especially for those navigating hardship while belonging in healthy communities foster positive religious coping (Pargament, 1997). According to religious coping theory (Pargament, 1997), attending religious services places individuals within a context rich in resources that offer significant support and guidance for the coping process that significantly impacts the outcomes of coping. For instance, through communal prayers, shared narratives of perseverance, and spiritual teachings, attendees gain access to collective wisdom and emotional support. These practices (in addition to participating in group rituals or receiving counsel from religious leaders) equip individuals with practical and emotional tools to navigate life's challenges, thereby shaping and reinforcing their sense of hope.

Conversely, research on adverse childhood experiences demonstrates that specific variables during childhood may reduce hope (VanderWeele, 2017). More specifically, studies show that adverse childhood experiences such as experiencing abuse, poor health, or a lower financial status during the early years of life can undermine hope by instilling patterns of learned helplessness, despair, or hopelessness early on (Anda et al., 2010; Conger & Donnellan, 2007). Being perceived as an outsider in one’s community or family, whether due to race, ethnicity, or other factors, can also exacerbate feelings of disconnection and alienation (Utset et al., 2002; Sheehy-Skeffington, 2020), potentially diminishing hope unless countered by strong supportive relationships or community bonds. Recent research suggests that the influence of factors which discouraged hope and other positive psychological experiences such as a sense of dignity during childhood can be diminished in adulthood through involvement in strong communities, and engagement in meaningful work (Gibson, 2022).

Childhood ‘predictors’ do not operate in isolation but can shape the individual’s future outlook (Anda et al., 2010), making the study of the developmental antecedents of hope complex, yet critical, for understanding and supporting human flourishing. Studying these early experiences can help researchers and practitioners to better understand hope and design interventions that address the early onset of hopelessness, tailoring support to reinforce hope across diverse life trajectories. This research is part of a larger project examining childhood predictors of human flourishing on a broader scale (VanderWeele & Johnson, 2025a, b). To maintain consistency across this coordinated set of parallel analyses, we employed standardized analytic methods to ensure that any observed differences are attributable to variations in constructs rather than methodological discrepancies. The shared methodological framework was specifically designed to provide a comprehensive perspective on how childhood experiences influence flourishing. The final selection of childhood predictors was carefully curated to represent a wide range of potential determinants, and the multi-phase process used to choose these indicators is detailed elsewhere (Lomas et al., 2025; Padgett et al., in press-a; in press-b). The selection of items was primarily guided by theoretical considerations and developed in consultation with collaborators at Gallup, who oversaw the sampling process, to determine which items were suitable for inclusion. However, challenges related to multicollinearity necessitated the omission or modification of certain conceptually distinct variables. While these adjustments aimed to minimize such issues, they may still arise for specific outcomes or within certain countries (please see Open Science Framework GFS codebook or related materials; Crabtree et al., 2024). We will henceforth adopt ‘childhood predictors’ as a term to capture the childhood experiences that may contribute to adult hope.

To address the above-mentioned questions, our study examines a number of potential childhood antecedents of adult self-rated hope in a multinational sample of more than 200,000 individuals from 22 countries. We explore the links between retrospectively reported childhood experiences and self-rated hope in adulthood, guided by three research questions and corresponding hypotheses. The first research question investigates how various factors during a child's upbringing—ranging from personal attributes to familial and social environments—are associated with self-rated hope in later life. We hypothesize that childhood experiences across family dynamics (e.g., parental marital status, relationship quality, etc.), socioeconomic conditions (e.g., perceived family income, immigration status, etc.), self-rated childhood health, religious engagement, and adverse experiences (e.g., physical and sexual abuse, feeling like an outsider in the family) will be associated with adult self-rated hope, with positive relational, health, and religious experiences correlating with greater hope and adverse or disrupted contexts associated with lower hope. The second research question extends our initial inquiry, which assessed the consistency of these associations across different countries. We expect that the magnitude and direction of the relationship between childhood experiences and adult self-rated hope will vary by country, considering the different sociocultural, economic, and health systems that shape how individual experiences and broader societal factors contribute to hopeful aspirations. The third research question examines the durability of these observed relationships against potential biases from unmeasured variables. We hypothesize that the observed relationships will remain robust under sensitivity analyses for unmeasured confounding (e.g., using E-values), suggesting that childhood experiences have a durable association with adult hope, even under different scenarios. Findings from these hypotheses are expected to offer valuable evidence for informing perspectives, policy and practice aimed at fostering hope throughout an individual's lifespan.

## Methods

The description of the methods below has been adapted from VanderWeele et al. (in press). Further methodological detail is available elsewhere (Crabtree et al., 2021; Johnson et al., 2024; Lomas et al., 2025; Padgett et al., 2024; Ritter et al., 2024).

### Population

The Global Flourishing Study (GFS) is a study of 202,898 participants from 22 geographically and culturally diverse countries, with nationally representative sampling within each country, concerning the distribution of determinants of well-being. Wave 1 of the data included the following countries and territories: Argentina, Australia, Brazil, Egypt, Germany, Hong Kong, India, Indonesia, Israel, Japan, Kenya, Mexico, Nigeria, the Philippines, Poland, South Africa, Spain, Sweden, Tanzania, Türkiye, United Kingdom, and the United States. The countries were selected to (a) maximize coverage of the world's population, (b) ensure geographic, cultural, and religious diversity, and (c) prioritize feasibility and existing data collection infrastructure. Data collection was carried out by Gallup Inc. Data for Wave 1 were collected principally during 2023, with some countries beginning data collection in 2022 and exact dates varying by country (Ritter et al., 2024). Four additional waves of panel data on the participants are intended to be collected annually from 2024–2027. The precise sampling design to ensure nationally representative samples varied by country and further details are available in Ritter et al. (2024). Survey items included aspects of well-being such as happiness, health, meaning, character, relationships, and financial stability (VanderWeele, 2017), along with other demographic, social, economic, political, religious, personality, childhood, community, health, and well-being variables. The data are publicly available through the Center for Open Science (https://www.cos.io/gfs). During the translation process, Gallup adhered to the TRAPD model (translation, review, adjudication, pretesting, and documentation) for cross-cultural survey research (ccsg.isr.umich.edu/chapters/translation/overview). Further details about methodology and survey development are documented in the GFS Questionnaire Development Report (Crabtree et al., 2021), GFS Methodology (Ritter et al., 2024), and GFS Translations documents (Johnson et al., 2023).

### Measures

#### Candidate Childhood Predictors

We assessed several predictors at baseline, organized into specific categories: demographic, socioeconomic, family dynamics and relationships, health and well-being, and religious and spiritual predictors. For additional details on the measures, see the codebook or Crabtree et al. (2024).

##### Demographic Predictors

Age was classified into groups: 18–24, 25–29, 30–39, 40–49, 50–59, 60–69, 70–79, and 80 + . Participants were asked, "What is your age? Please enter your age below." Gender was assessed with the question, "What is your gender?" with response options being Male, Female, or Other. Immigration status was determined by asking, "Were you born in this country, or not?" with response options of Yes or No. Racial/ethnic identity was assessed in some countries with response categories varying by country.

##### Socioeconomic Predictors

Subjective financial status during childhood was assessed with the question, "Which one of these phrases comes closest to your own feelings about your family's household income when you were growing up, such as when YOU were around 12 years old?" The response options included Lived comfortably, Got by, Found it difficult, and Found it very difficult.

##### Family Dynamics and Relationship Predictors

Parent marital status and family structure during childhood were assessed by asking, "Were your parents married to each other when YOU were around 12 years old?" with response options being Married, Divorced, Never married, or One or both had died. Relationships with parents were assessed with questions such as, "Please think about your relationship with your mother when you were growing up. In general, would you say that relationship was…," with response options of Very good, Somewhat good, Somewhat bad, or Very bad. Responses were dichotomized into Very/Somewhat good versus Very/Somewhat bad. An analogous question assessed the relationship with the father. For respondents without a mother or father due to death or absence, "Does not apply" was treated as a dichotomous control variable. Additionally, feelings of being an outsider in the family were gauged with, "When you were growing up, did you feel like an outsider in your family?" with Yes or No as response options. Experiences of abuse were measured by asking, "Were you ever physically or sexually abused when you were growing up?" with Yes or No responses "(this was not asked in Israel and therefore not part of the meta-analyses)."

##### Self-Rated Health/Well-being Predictor

Self-rated health during childhood was assessed by asking, "In general, how was your health when you were growing up?" with response options of Excellent, Very good, Good, Fair, or Poor.

##### Religious and Spiritual Predictors

Religious service attendance at age 12 was measured with the question, "How often did YOU attend religious services or worship at a temple, mosque, shrine, church, or other religious building when YOU were around 12 years old?" Responses included At least once/week, One-to-three times/month, Less than once/month, or Never. Childhood religious tradition/affiliation included categories such as Christianity, Islam, Hinduism, Buddhism, Judaism, Sikhism, Baha’i, Jainism, Shinto, Taoism, Confucianism, Primal/Animist/Folk religion, Spiritism, African-Derived, some other religion, or no religion/atheist/agnostic, with precise categories varying by country.

### Outcome Variable

Hope about the future was retrospectively assessed with the question, "Despite challenges, I always remain hopeful about the future" (Lomas et al., 2025; Peterson & Saligman, 2004). Response options ranged from 0 = Strongly disagree to 10 = Strongly agree. Higher scores indicated higher levels of hope.

### Analysis

Descriptive statistics for the observed sample, weighted to be nationally representative within country, were estimated for each childhood demographic category. A weighted linear regression model with complex survey adjusted standard errors was fit within each country for hope on all of the aforementioned childhood predictor variables simultaneously.

In the primary analyses, random effects meta-analyses of the regression coefficients (Borenstein et al., 2010; Hunter and Schmidt, 2000) along with confidence intervals, lower and upper limits 95% prediction intervals, heterogeneity (τ), and I^2^ for evidence concerning variation within a given predictive component across countries (Mathur & VanderWeele, 2020). Forest plots of estimates are available in the online supplement. Religious affiliation/tradition and race/ethnicity were used within country as control variables, when available, but these coefficients themselves were not included in the meta-analyses since categories/responses varied by country.

All meta-analyses were conducted in R (R Core Team, 2024) using the metafor package (Viechtbauer, 2010). Within each country, a global test of association of each childhood predictor variable group with the outcome of hope was conducted, and a pooled p-value (Wilson, 2019) across countries reported concerning evidence for association within any country. Bonferroni corrected p-value thresholds are provided based on the number of childhood predictor variables (Abdi, 2007; VanderWeele and Mathur, 2019). For each childhood predictor, we calculated E-values to evaluate the sensitivity of results to unmeasured confounding. An E-value is the minimum strength of the association an unmeasured confounder must have with both the outcome and the predictor, above and beyond all measured covariates, for an unmeasured confounder to explain away an association (VanderWeele and Ding, 2017). As a supplementary analysis, population weighted meta-analyses of the regression coefficients were estimated. All analyses were pre-registered with COS prior to data access, with only slight subsequent modification in the regression analyses due to multicollinearity (https://osf.io/y9kh6); all code to reproduce analyses are openly available in an online repository (Padgett et al., 2024).

#### Missing Data

Missing data on all variables was imputed using multivariate imputation by chained equations, and five imputed datasets were used (Acock, 2005; Rubin, 1996; Sterne et al., 2009; Van Burren, 2023). To account for variation in the assessment of certain variables across countries (e.g., religious affiliation/tradition and race/ethnicity), the imputation process was conducted separately in each country. This within-country imputation approach ensured that the imputation models accurately reflected country-specific contexts and assessment methods. Sampling weights were included in the imputation models to account for specific-variable missingness that may have been related to probability of inclusion in the study.

#### Accounting for Complex Sampling Design

The GFS used different sampling schemes across countries based on availability of existing panels and recruitment needs (Ritter et al., 2024). All analyses accounted for the complex survey design components by including weights, primary sampling units, and strata. Additional methodological detail, including accounting for the complex sampling design, is provided elsewhere.

## Results

### Descriptive Statistics

Table 1 provides the distribution of descriptive statistics. The ages of participants in the observed sample ranged the entire adult lifespan (18–80 +). The gender distribution was nearly balanced, with 51% female, 48% male, and a small representation from ‘other’ gender identities (0.3%). A majority of participants were married (52%), had attained 9–15 years of education (57%), and were employed (39%). Most were native-born (94%). Religious service attendance varied, with most never attending (37%), some attending once a week (19%), and a minority of the sample attending once a week or more (13%).

**Table 1:** Nationally representative childhood descriptive statistics of the observed sample

**Characteristic**	**N = 202,898**^1^
**Relationship with mother**	
Very good	127,836 (63%)
Somewhat good	52,439 (26%)
Somewhat bad	11,060 (5.5%)
Very bad	4,642 (2.3%)
Does not apply	5,965 (2.9%)
Missing	956 (0.5%)
**Relationship with father**	
Very good	107,742 (53%)
Somewhat good	55,714 (27%)
Somewhat bad	15,807 (7.8%)
Very bad	8,278 (4.1%)
Does not apply	13,985 (6.9%)
Missing	1,372 (0.7%)
**Parent marital status**	
Yes, married	152,001 (75%)
No, divorced	17,726 (8.7%)
Never married	15,534 (7.7%)
No, one or both of them had died	7,794 (3.8%)
Missing	9,843 (4.9%)
**Subjective financial status of family growing up**	
Lived comfortably	70,861 (35%)
Got by	82,905 (41%)
Found it difficult	35,852 (18%)
Found it very difficult	12,606 (6.2%)
Missing	674 (0.3%)
**Abuse**	
Yes	29,139 (14%)
No	167,279 (82%)
Missing	6,479 (3.2%)
**Outsider growing up**	
Yes	28,732 (14%)
No	170,577 (84%)
Missing	3,589 (1.8%)
**Self-rated health growing up**	
Excellent	67,121 (33%)
Very good	63,086 (31%)
Good	47,378 (23%)
Fair	19,877 (9.8%)
Poor	4,906 (2.4%)
Missing	530 (0.3%)
**Immigration status**	
Born in this country	190,998 (94%)
Born in another country	9,791 (4.8%)
Missing	2,110 (1.0%)
**Age 12 religious service attendance**	
At least 1/week	83,237 (41%)
1–3/month	33,308 (16%)
< 1/month	36,928 (18%)
Never	47,445 (23%)
Missing	1,980 (1.0%)
**Age group**	
1998–2005; current age: 18–24	27,007 (13%)
1993–1998; age: 25–29	20,700 (10%)
1983–1993; age: 30–39	40,256 (20%)
1973–1983; age 40–49	34,464 (17%)
1963–1973; age 50–59	31,793 (16%)
1953–1963; age 60–69	27,763 (14%)
1943 or earlier; age 80 +	4,119 (2.0%)
1943–1953; age 70–79	16,776 (8.3%)
Missing	20 (< 0.1%)
**Gender**	
Male	98,411 (49%)
Female	103,488 (51%)
Other	602 (0.3%)
Missing	397 (0.2%)
**Country**	
Argentina	6,724 (3.3%)
Australia	3,844 (1.9%)
Brazil	13,204 (6.5%)
Egypt	4,729 (2.3%)
Germany	9,506 (4.7%)
India	12,765 (6.3%)
Indonesia	6,992 (3.4%)
Israel	3,669 (1.8%)
Japan	20,543 (10%)
Kenya	11,389 (5.6%)
Mexico	5,776 (2.8%)
Nigeria	6,827 (3.4%)
Philippines	5,292 (2.6%)
Poland	10,389 (5.1%)
South Africa	2,651 (1.3%)
Spain	6,290 (3.1%)
Tanzania	9,075 (4.5%)
Turkey	1,473 (0.7%)
United Kingdom	5,368 (2.6%)
United States	38,312 (19%)
Sweden	15,068 (7.4%)
Hong Kong (special administrative region of China)	3,012 (1.5%)

^1^n (%)

### Meta-Analytic Results

Table 2 shows the associations between the 11 childhood candidate experiences and hope in adulthood. Several childhood factors were positively associated with an increased sense of hope in adulthood, including experiencing excellent (β = 0.48, 95% CI: 0.31, 0.65) or very good health growing up (β = 0.23, 95% CI: 0.12, 0.34), and regular attendance at religious services at age 12, ranging from at least once/week (β = 0.32, 95% CI: 0.25, 0.40), to 1–3 times/month (β = 0.21, 95% CI: 0.10, 0.32), and less than once/month (β = 0.11, 95% CI: 0.04, 0.18). Having a very or somewhat good relationship with the mother (β = 0.12, 95% CI: -0.00, 0.23) and father (β = 0.14, 95% CI: 0.09, 0.19), living in a family that comfortably met its financial needs (β = 0.09, 95% CI: 0.01, 0.16), and being female (β = 0.08, 95% CI: 0.01, 0.15). Additionally, there was a gradual and positive association between age and levels of hope, where estimated coefficients ranged from 0.06 for age 25–29 to 0.44 for age 80 + , indicating a progressive increase in hope with advance in age.

**Table 2:** Random effects meta-analysis of regression of hope on childhood predictors

					Estimated Proportion of Effects by Threshold				
Variable	Category	Est	95% CI	SE	< -0.10	> 0.10	Heterogeneity (τ)	I^2	Global p-value
Relation- ship with mother	(Ref: Very bad/some what bad)								< .001**
	Very good/somewhat good	0.12	(-0.00,0.23)	0.06	0.18	0.55	0.21	69.6	
Relation- ship with father	(Ref: Very bad/some what bad)								< .001**
	Very good/somewhat good	0.14	(0.09,0.19)	0.02	0.00	1.00	0.02	4.5	
Parent marital status	(Ref: Parents married)								< .001**
	No, divorced	-0.03	(-0.15,0.09)	0.06	0.27	0.27	0.24	79.4	
	Single, never married	-0.01	(-0.11,0.09)	0.05	0.23	0.18	0.18	65.8	
	No, one or both of them had died	-0.01	(-0.13,0.10)	0.06	0.36	0.27	0.20	58.2	
Subjective financial status of family growing up	(Ref: Got by)								< .001**
	Lived comfortably	0.09	(0.01,0.16)	0.04	0.05	0.36	0.16	84.0	
	Found it difficult	-0.00	(-0.05,0.04)	0.02	0.05	0.05	0.06	28.0	
	Found it very difficult	-0.06	(-0.17,0.04)	0.05	0.41	0.27	0.17	51.4	
Abuse	(Ref: No)								< .001**
	Yes	-0.16	(-0.22,-0.10)	0.03	0.76	0.00	0.09	44.1	
Outsider growing up	(Ref: No)								< .001**
	Yes	-0.20	(-0.29,-0.12)	0.04	0.73	0.00	0.16	67.8	
Self-rated health growing up	(Ref: Good)								< .001**
	Excellent	0.48	(0.30,0.67)	0.09	0.00	0.91	0.43	95.7	
	Very good	0.23	(0.12,0.34)	0.06	0.00	0.68	0.24	89.0	
	Fair	-0.13	(-0.25,-0.01)	0.06	0.73	0.18	0.23	79.8	
	Poor	-0.18	(-0.35,-0.01)	0.09	0.64	0.18	0.28	56.5	
Immigra- tion status	(Ref: Born in this country)								< .001**
	Born in another country	0.06	(-0.13,0.26)	0.10	0.41	0.50	0.40	86.4	
Age 12 religious service attendance	(Ref: Never)								< .001**
	At least 1/week	0.32	(0.25,0.40)	0.04	0.00	0.95	0.13	52.2	
	1–3/month	0.21	(0.10,0.32)	0.05	0.05	0.64	0.21	74.1	
	< 1/month	0.11	(0.04,0.18)	0.04	0.00	0.50	0.10	51.3	
Year of birth	(Ref: 1998–2005; age 18–24)								< .001**
	1993–1998; age 25–29	0.05	(-0.02,0.12)	0.04	0.09	0.32	0.12	52.6	
	1983–1993; age 30–39	0.04	(-0.04,0.13)	0.04	0.18	0.32	0.17	73.0	
	1973–1983; age 40–49	0.04	(-0.08,0.16)	0.06	0.32	0.41	0.25	83.1	
	1963–1973; age 50–59	0.09	(-0.07,0.25)	0.08	0.27	0.41	0.37	90.2	
	1953–1963; age 60–69	0.07	(-0.10,0.24)	0.09	0.36	0.55	0.37	87.8	
	1943 or earlier; age 80 +	0.13	(-0.20,0.45)	0.17	0.32	0.55	0.67	86.1	
	1943–1953; age 70–79	0.02	(-0.21,0.25)	0.12	0.41	0.41	0.49	88.3	
Gender	(Ref: Male)								< .001**
	Female	0.08	(0.01,0.15)	0.03	0.05	0.45	0.14	85.5	
	Other	-0.44	(-0.81,-0.06)	0.19	0.78	0.17	0.63	77.5	

*Note.* *p < .05; **p < .004 (Bonferroni corrected threshold)

Conversely, several childhood factors were associated with a decreased sense of hope in adulthood. These included experiencing abuse (β = -0.16, 95% CI: -0.39, -0.23), feeling like an outsider growing up (β = -0.29, 95% CI: -0.39, -0.20), experiencing fair health growing up (β = -0.20, 95% CI: -0.29, -0.12), living in a family where it was very difficult to meet financial needs (β = -0.06, 95% CI: -0.17, -0.04), and one or both parents dying during childhood (β = -0.01, 95% CI: -0.13, -0.10). There was little evidence that childhood immigration status was associated with hope in adulthood. These population-weighted meta-analytic results are consistent with supplementary results (Tables S23, S24; also see forest plots Figures S1 to S27).

### Do Associations Vary by Country?

The random effects meta-analysis revealed substantial cross-country heterogeneity in associations between early-life conditions and hope in adulthood. Self-rated health in childhood showed high heterogeneity (τ = 0.41, I^2^ = 75.9%), indicating substantial variation in its effects on future hope across different cultural and social contexts (see Supplementary Table S2-S24). For example, in Indonesia, excellent self-rated health in childhood was a moderately positive predictor of hope (Table S7b: β = 0.27, 95% CI: 0.15, 0.40), whereas in India, the impact was less pronounced (Table S6b: β = 0.13, 95% CI: -0.06, 0.32). Parental marital status also exhibited considerable variability (τ = 0.25–0.26, I^2^ > 80% for children of divorced or never-married parents), suggesting diverse effects of family stability on hope. In Germany, children from divorced families did not show significant differences in hope compared to those from married families (Table S5b: β = -0.03, p = 0.908), while in Poland children of divorced parents had lower levels of adult hope (β = -0.65, 95% CI: -0.89, -0.40) and in Sweden, children of divorced parents in fact had higher hope levels (Table S6b: β = 0.20, 95% CI: 0.05, 0.35).

The economic background during upbringing, indicated by subjective financial status (τ = 0.23 for 'found it very difficult,' I^2^ = 69.4%), showed significant heterogeneity, highlighting the varied impact of childhood financial stability on hope across countries. For example, 'found it very difficult' (compared to ‘got by’) was negatively associated with hope in India (Table S6b: β = -0.50, 95% CI: -0.70, -0.30) but had some evidence for a moderate positive effect in the Philippines (Table S13b: β = 0.30, 95% CI: -0.02, 0.61). Additionally, the effect of birth year exhibited high heterogeneity, which increased with age (e.g., comparing 18–24 year-olds to 30–39 year-olds, τ = 0.25, I^2^ = 87.4, and comparing them to 60–69 year-olds, τ = 0.54, I^2^ = 94.6). The effect of birth year also varied significantly across countries, with younger age groups (18–24 year-olds) displaying higher levels of hope compared to older age groups (60–69 year-olds) in Germany (Table S5b: β = 0.22, 95% CI: -0.01, 0.45) and lower levels of hope in Indonesia (Table S7b: β = -0.39, p < 0.001). Noteworthy outliers included Egypt, where gender (being female) was a strong positive predictor of hope (Table S4b: β = 0.64, 95% CI: 0.45, 0.83), and the Philippines, where the presence of abuse did not significantly impact hope (Table S9b: β = -0.03, p = 0.707).

Certain childhood experiences consistently showed positive associations with adult hope across countries, such as excellent or very good health growing up and attending religious services at least once a week or 1–3 times/month. For instance, in Indonesia, excellent self-rated health was a strong positive predictor (Table S7b: β = 0.27, 95% CI: 0.15, 0.40), and attending religious services at least once a week was associated with higher hope levels in Egypt (Table S4b: β = 0.44, 95% CI: 0.21, 0.67). Predictors such as experiencing abuse or feeling like an outsider during childhood were almost universally negative. In Germany, experiencing abuse had a significant negative impact on hope (Table S5b: β = -0.20, 95% CI: -0.37, -0.03), and feeling like an outsider was similarly detrimental in Brazil (Table S3b: β = -0.38, 95% CI: -0.55, -0.20).

Other predictors, such as relationships with parents, financial comfort during childhood, and attending religious services less than once a month, generally had positive associations but with considerable variability in magnitude across countries. In Argentina, a good relationship with the mother positively influenced hope (Table S1b: β = 0.41, 95% CI: 0.13, 0.69), while living financially comfortably during childhood (compared to those who ‘got by’) was positively associated with hope in India (Table S6b: β = -0.23, 95% CI: -0.38, -0.08). Some childhood experiences, like experiencing financial difficulty or having fair health, showed mostly negative non-significant associations with some variability. For example, in Indonesia, financial difficulty was negatively associated with hope (Table S7b: β = -0.05, p = 0.18), while fair health showed negative associations in Egypt (Table S4b: β = -0.20, p = 0.20).

Factors such as childhood immigration status and being born female had mixed associations, positive in some countries and negative in others. In South Africa, being born female (vs. male) had a negative association with hope (Table S15b: β = -0.23, 95% CI: -0.44, -0.02), whereas in Egypt, it was positively associated (Table S4b: β = 0.64, 95% CI: 0.45, 0.83). This highlights the diverse cultural contexts and emphasizes the need for tailored approaches in understanding and fostering hope across different regions.

### Additional Analyses

E-values suggested that many of the observed associations were moderately robust to unmeasured confounding (Table 3). For instance, for excellent (vs. good) childhood health, an unmeasured confounder associated with both better health and higher adult hope by risk ratios of 1.78 each could explain away the association, but weaker joint confounder associations could not. To shift the confidence interval to include the null, an unmeasured confounder associated with both the better childhood health and higher adult hope by risk ratios of 1.54 each would suffice. Detailed country-specific results are available in the Appendix.

**Table 3:** Sensitivity of meta-analyzed childhood predictors to unmeasured confounding

Variable	Category	E-value	E-value confidence interval limit
Relationship with mother	(Ref: Very bad/somewhat bad)		
	Very good/somewhat good	1.29	1.00
Relationship with father	(Ref: Very bad/somewhat bad)		
	Very good/somewhat good	1.32	1.25
Parent marital status	(Ref: Parents married)		
	No, divorced	1.12	1.00
	Single, never married	1.07	1.00
	No, one or both of them had died	1.08	1.00
Subjective financial status of family growing up	(Ref: Got by)		
	Lived comfortably	1.24	1.09
	Found it difficult	1.04	1.00
	Found it very difficult	1.20	1.00
Abuse	(Ref: No)		
	Yes	1.36	1.26
Outsider growing up	(Ref: No)		
	Yes	1.41	1.29
Self-rated health growing up	(Ref: Good)		
	Excellent	1.78	1.54
	Very good	1.45	1.30
	Fair	1.31	1.08
	Poor	1.38	1.08
Immigration status	(Ref: Born in this country)		
	Born in another country	1.20	1.00
Age 12 religious service attendance	(Ref: Never)		
	At least 1/week	1.57	1.47
	1–3/month	1.42	1.27
	< 1/month	1.28	1.15
Year of birth	(Ref: 1998–2005; age 18–24)		
	1993–1998; age 25–29	1.17	1.00
	1983–1993; age 30–39	1.16	1.00
	1973–1983; age 40–49	1.15	1.00
	1963–1973; age 50–59	1.25	1.00
	1953–1963; age 60–69	1.21	1.00
	1943 or earlier; age 80 +	1.30	1.00
	1943–1953; age 70–79	1.09	1.00
Gender	(Ref: Male)		
	Female	1.23	1.08
	Other	1.72	1.20

## Discussion

This cross-national analysis of childhood predictors of hope offers insights into how early-life conditions may shape future hope across diverse cross-national contexts. Key meta-analytic findings indicate that positive childhood experiences, such as excellent or very good health, supportive parental relationships, and regular religious attendance, are strongly associated with higher levels of hope in adulthood. Negative experiences like abuse and feeling like an outsider during childhood are linked to lower levels of hope. Substantial cross-country heterogeneity was also revealed in the associations between early-life conditions and hope in adulthood. For instance, self-rated childhood health showed a moderately positive effect on adult hope in Indonesia, while the impact was less pronounced in India. Similarly, the effects of parental marital status varied, with no significant differences in hope among children from divorced families in Germany, negative effects of childhood parental divorce in Poland, and positive effects in Sweden. Similar variations were seen in younger age groups generally showing higher levels of hope compared to older age groups, but with notable exceptions. For example, in Germany, younger individuals exhibited significantly higher levels of hope than older age groups. In contrast, in Indonesia, younger age was associated with lower levels of hope compared to older individuals. These findings suggest that the association between childhood predictors and hope is highly context-dependent, varying considerably across cultural and social environments. This variability across different countries highlights the importance of spatial—cultural and social—contexts in understanding hope (Counted & Newheiser, 2024). The significant role of childhood health and parental relationships in fostering hope highlights the foundational importance of these early experiences in developing the cognitive and emotional resources necessary for hope.

Taken together, the findings largely confirm our three guiding hypotheses. First, we found robust evidence that a range of childhood experiences—including excellent self-rated health, supportive parental relationships, religious attendance, and financial stability—were positively associated with higher levels of adult hope, while adverse experiences such as abuse and feeling like an outsider predicted lower hope, affirming the hypothesis that recalled childhood conditions associate with hopeful orientation in adulthood. Second, consistent with our expectations, the strength and direction of these associations varied considerably across countries, reflecting sociocultural, economic, and institutional differences in how childhood environments are experienced and interpreted. Third, sensitivity analyses indicated that many of these associations remained robust under potential unmeasured confounding, supporting the durability of these relationships. Collectively, these findings highlight both the universal and context-specific nature of hope’s developmental antecedents and highlight the need for culturally and contextually sensitive strategies in sustaining and strengthening hope across the lifespan.

## Parental Relationships

The quality of parental relationships emerged as a critical childhood predictor of hope. A very good relationship with one's mother and father was positively associated with higher levels of hope. This finding aligns with attachment theory, which posits that secure and supportive parental bonds provide a foundation for developing cognitive and emotional resilience (Bowlby, 2008; Shorey et al., 2003). However, the impact of parental marital status varied significantly across countries. For instance, in Germany, children from divorced families did not show significant differences in hope compared to those from married families, possibly due to a robust welfare system (Kreyenfeld et al., 2023; Sheehy-Skeffington, 2020). Conversely, children from divorced families (compared to married ones) in at least Sweden reported increased perceptions of hope, though this may be due to the robust welfare systems and low stigma surrounding divorce which may create supportive environments that mitigate conflict and promote resilience, thus fostering hope. However, in India, both children of divorced parents and having one or both parents deceased exhibited higher hope levels than married ones. Perhaps the low divorce rate and cultural stigma associated with it often mean that when divorce does occur, it tends to reflect the resolution of extreme family conflict rather than a common or casual decision (Masten, 2001; Rutter, 1987). For children, this could signify a transition to a more peaceful and stable environment that fosters a sense of relief and hope. Similarly, the loss of a parent through death might shield children from ongoing exposure to abusive or dysfunctional relationships. In such cases, the collectivist nature of Indian society, with its emphasis on extended family support, can provide emotional and material stability, compensating for the loss (Conger & Donnellan, 2007; Thadathil & Sriram, 2020). Such collectivist factors, combined with cultural narratives that emphasize resilience and adaptation in the face of adversity, may instill a sense of hope and possibility in children, helping them to view challenges as opportunities for growth and progress (Dommaraju, 2016). These contrasting results suggest a complex dynamic between family structure and hope that varies across contexts and warrants further studies.

## Financial Status

Childhood financial stability was another significant predictor, with those growing up in financially comfortable families generally exhibiting higher levels of hope. This positive association underscores the role of economic security in providing a stable environment conducive to developing hopeful outlooks (Anda et al., 2010; Conger & Donnellan, 2007). Conversely, the impact of financial difficulty during childhood also showed significant variability. In Indonesia, for example, financial difficulties had a substantial negative impact on hope, highlighting the acute effects of economic disparities (Hendriks et al., 2018). In contrast, Brazil showed a more moderate effect, suggesting that other cultural factors may mitigate the adverse effects of financial instability. These factors may include strong social networks that provide emotional and practical support, collective coping mechanisms that foster a sense of shared burden, and robust government and social programs aimed at reducing poverty and inequality (De Araújo et al., 2020). Besides, the Brazilian cultural spirit of resilience is often associated with 'jeitinho simpático', a socio-cultural strategy for navigating challenges. While commonly referred to as 'jeitinho brasileiro,' it’s important to distinguish between its two dimensions: 'Jeitinho Simpático'—marked by creativity, conflict avoidance, and positive social interaction—and 'Jeitinho Malandro', which involves deception and manipulation (Akira Miura et al., 2019). In the context of flourishing and adaptive coping, 'Jeitinho Simpático' better captures the hopeful, relational creativity that helps Brazilians persist through adversity (De Araújo et al., 2020; Long et al., 2024).

## Health

Self-rated health during childhood emerged as one of the most significant predictors of adult hope. The meta-analysis indicated that individuals who rated their health as "excellent" or "very good" during childhood showed notably higher levels of hope in adulthood. These findings align with the idea that physical well-being provides a foundation for cognitive and emotional resilience (Snyder et al., 1991). According to the biopsychosocial model, physical health impacts psychological states and social functioning, thereby facilitating a more hopeful outlook on life (Engel, 1977). However, this association varied across countries. In more developed countries like Germany (estimate: 0.63, 95% CI: 0.48, 0.78) and Japan (estimate: 1.32, 95% CI: 1.20, 1.43), the strong link between excellent self-rated health and hope highlights the potential importance of robust healthcare systems and cultural emphasis on health. In contrast, countries facing substantial socio-economic challenges, such as India (estimate: 0.13, 95% CI: -0.06, 0.32), showed weaker associations or no substantial associations (e.g., Nigeria, with an estimate: -0.03, 95% CI: -0.24, 0.18, p < 0.89), suggesting that broader socio-economic issues might overshadow the benefits of good health. Notably, the lack of significant associations in Egypt (estimate: -0.01, 95% CI: -0.33, 0.30) and Kenya (estimate: 0.10, 95% CI: -0.06, 0.26) points to the presence of other dominant factors influencing hope, such as socio-cultural environments and economic instability. These patterns suggest that while health is a fundamental predictor of hope, its impact may be shaped by the socio-economic and cultural environment (Counted & Newheiser, 2024). The strong positive association between excellent self-rated health in childhood and future hope in many countries may also reflect the importance placed on physical well-being as a critical foundation for a positive outlook on life (Engel, 1977; Long et al., 2024). Understanding these spatial interactions that sustain or inhibit hope can inform targeted interventions that consider both health and socio-economic and cultural contexts to foster hope and resilience in children worldwide.

## Religious Attendance

Regular attendance at religious services during childhood was positively associated with higher levels of hope, particularly when attending services at least once a week or 1–3 times a month. This association highlights the role of religious communities in providing social support and a framework for understanding life's challenges, thereby fostering a hopeful outlook (Koenig, 2013; Pargament, 1997; Pawlikoswki et al., 2019). However, the impact of religious attendance varied significantly across countries. In Egypt, attending religious services at least once a week was a strong predictor of hope. In contrast, in Germany, the association was also positive but less pronounced for weekly attendance. This may suggest that while religious participation contributes to hope in some countries (e.g., Egypt), other factors may play a more significant role in shaping future hope in more secularized societies. Similarly, in Poland, the positive effects of weekly religious attendance on hope indicates a strong link between religious engagement and hope, reflecting the deep-rooted cultural and historical importance of religion in Polish society (Borowik, 2016). However, in Sweden, the effect of attending religious services was less consistent. While the same positive association is seen at the highest levels of attendance and less frequent attendees < 1/month (compared to the ‘Never’ attended), the middle category of 1–3/month did not show the same positive association that the meta-analysis demonstrated. These variations emphasize the need to consider cultural and socio-economic contexts when examining the determinants of hope, as the impact of religious attendance is shaped by broader societal norms and values (Counted & Newheiser, 2024).

## Adverse Experiences

Adverse childhood experiences, such as abuse and feeling like an outsider, were associated with lower levels of hope, highlighting the potentially detrimental effects of negative early experiences on hope. This supports previous findings on the long-term impact of childhood trauma (Anda et al., 2010), where repeated exposure to uncontrollable negative events leads to a pervasive sense of hopelessness (Anda et al., 2010; Conger & Donnellan, 2007). However, the impact of these adverse experiences varied significantly across countries. In countries with a greater emphasis on individualism like the United States and the United Kingdom, the association between adverse experiences and diminished hope was particularly strong, indicating that a lack of social support may exacerbate the negative effects of trauma. Conversely, in countries like Brazil and Mexico, factors such as stronger family ties and community support systems may have mitigated the impact of adverse experiences on hope, providing a buffer against early trauma. In contrast, in regions with high levels of socio-economic instability, such as countries in sub-saharan Africa (e.g., Kenya, Nigeria), the impact of adverse experiences on hope was pronounced, highlighting the compounded effect of systemic challenges and personal trauma.

## Theoretical Considerations and Practical Implications

Theoretically, this study highlights the need to contextualize constructs like hope within specific cultural and socio-economic frameworks (see spatial hope (Counted & Newheiser, 2024). The observed mean values across countries and demographic factors suggests that universal theories of hope must be adapted and broadly conceptualized to account for deeper cross-cultural comparisons. This study also emphasizes the complex effects of individual, familial, and societal childhood factors that shape hope, advocating for integrative models that capture these dynamics (Gallagher & Lopez, 2017). For instance, the economic background of an individual during upbringing exhibited significant heterogeneity in many countries, highlighting the varied impact of childhood financial stability on hope. This finding hints at the importance of designing socio-economic interventions that are deeply rooted in local contexts in order to advance efficacious approaches (Sheehy-Skeffington, 2020). For instance, in India, where financial difficulty during childhood was linked to lower levels of hope, interventions such as promoting Self Help Groups (SHG’s) that encourage individuals to pool their resources, manage their finances, and reduce bad loans have been shown to reduce and prevent financial vulnerability (Biju, 2024). In contrast, in Brazil, where the impact of financial difficulty on hope was less pronounced, prioritizing initiatives that increase social connections and community, such as increasing cultural consonance through investment in leisure time, can serve as a way to foster hope (Dressler et al., 1997).

Cultural attitudes towards gender, family structure, and community engagement also play critical roles in shaping hope. In a number of countries being female was a strong positive predictor of hope, possibly reflecting cultural values that empower women in specific ways (Baron, 2005; Thomas et al., 2023). Such nuances highlight the need for culturally sensitive approaches in both research and practice, and warrant the need for country and context-specific intervention. These findings provide valuable insights for policymakers, educators, and mental health professionals. Interventions aimed at enhancing childhood health, fostering strong parental relationships, and encouraging and sustaining regular religious participation can be effective strategies for increasing hope and well-being in adulthood. However, these interventions must be culturally tailored to address the specific needs and contexts of different populations. For example, in countries with significant socio-economic disparities, promoting the aspirations of hope might simultaneously require addressing broader structural inequalities to be successful (Conger & Donnellan, 2007).

Study findings suggest that efforts to mitigate the adverse effects of childhood trauma must consider the availability and strength of social support systems. In principally individualistic societies (e.g., US and UK), where the association between adverse experiences and diminished hope is particularly strong, enhancing social support mechanisms could help buffer against the negative impacts of trauma on hope in adulthood. Conversely, in principally collectivist countries, such as Brazil and Mexico, leveraging existing family and community support structures can be a vital component of effective interventions. In regions with high levels of socioeconomic instability, such as parts of Kenya and Nigeria, comprehensive approaches that address both systemic challenges and personal trauma are likely necessary to foster hope.

## Limitations

This study has several limitations. One limitation of this paper is the reliance on a single-item outcome measure for hope, which sacrifices the depth and breadth of construct coverage. While the GFS offers a wide range of constructs and indicators with broad geographical coverage, many constructs are assessed using single-item measures. Although the large sample size partially mitigates concerns about statistical power, it does not address issues of reliability. The single-item assessments used in the study were mostly derived from existing scales and selected based on their strong correlation with other indicators to provide some basis for assessing reliability (please see Lomas et al., 2025 for how each item was extracted and developed). One notable limitation in the current study is the absence of a direct measure of emotional abuse, which, although it may be partially captured through the psychological sequelae of reported physical and sexual abuse, represents a distinct form of trauma with unique developmental consequences and thus warrants separate attention. Given the study’s goal of informing culturally sensitive policies and interventions to mitigate the effects of childhood trauma on hope, the exclusion of emotional abuse—a form of adversity with substantial psychological impact—represents a limitation that should be acknowledged. However, despite these considerations, concerns about the reliability of these measures remain a clear limitation of the GFS and are important to acknowledge. The reliance on self-reported data also introduces potential biases, including social desirability and recall biases. The childhood predictors analyses constitute a synthetic longitudinal study and thus may provide some evidence for causality, but should be interpreted with caution. While the data is essentially cross-sectional, the use of retrospectively reported childhood predictors still carries the potential bias of recall errors and retrospective reporting. However, for recall bias to completely explain away the observed associations would require that the effect of adult hope on biasing retrospective assessments of the childhood predictors would essentially have to be at least as strong as the observed associations themselves, and some of these were quite substantial (VanderWeele & Li, 2019). Looking ahead, future waves of data will provide further insights into potential causal relationships between hope and its potential determinants. A limitation of our modeling approach was the decision to enter all correlates simultaneously for each country. While this method ensured consistency across countries, aligned with the pre-registered analytical framework, and minimized potential biases from sequential entry, it may have restricted further context-specific insights. A systematic approach—prioritizing variable entry based on the strength of bivariate associations—could better align with the study’s emphasis on localized interpretations of flourishing (VanderWeele & Johnson, 2025a, b). Although the simultaneous entry approach served the study’s exploratory and comparative goals, future research could benefit from systematic modeling, especially in country-specific analyses.

The cultural and contextual diversity of the 22 countries studied is a key strength of this paper, offering rich insights into how hope is shaped across different contexts. However, this diversity also introduces variability that requires careful interpretation of the results. Future research should aim to address these limitations through longitudinal designs and more nuanced, culturally sensitive methodologies. Future research should also focus on longitudinal studies to understand the mechanisms by which childhood experiences affect hope in adulthood (and efforts are in place to collect additional waves of data through the Global Flourishing Study, thus allowing us to do just that). More in-depth, culturally and spatially specific analyses are needed to provide richer insights along these lines.

## Conclusions

This study provides a broad examination of the relation of childhood experiences and hope across a diverse set of countries. The findings show the importance of early-life conditions in fostering a hopeful outlook, while also highlighting the need for context-specific interpretations of data and interventions to promote hope. Integrating cultural and contextual considerations into the study of hope is essential for developing a more nuanced understanding of this psychological construct, which unfolds within the social and physical environments of one's country of origin (Counted & Newheiser, 2024). Richer conceptions of hope can improve cross-cultural comparisons and guide specific interventions. This is especially important in understanding how childhood experiences are associated with hope across different contexts.

## Supplementary Information

Below is the link to the electronic supplementary material.

Supplementary file1 (ZIP 7399 KB)

## Data Availability

Data are available for download at the Center for Open Science (COS) website (https://www.cos.io/gfs). All analyses were pre-registered with COS prior to data access (https://osf.io/y9kh6); all code to reproduce analyses are openly available in an online repository (Padgett et al., 2024).
