# Supplementary material for: Seeds of Hope: Cross-National Analysis of Childhood Predictors of Hope in 22 Countries
Source: Appl Res Qual Life. 2025 May 19;20(3):1111–37. doi: 10.1007/s11482-025-10450-0 (PMC12328542; doi:10.1007/s11482-025-10450-0)
Supplement: Supplementary file 1 — Supplementary file1 (ZIP 7399 KB) [file 11482_2025_10450_MOESM1_ESM.zip › Supplementary Tables and Plots - Hope _ childhood predictors .docx]

***Table S23. Population weighted meta-analysis of regression results.***

| Variable | Category | Est | 95% CI | SE |
| --- | --- | --- | --- | --- |
| Relationship with mother | (Ref: Very bad/somewhat bad) |  |  |  |
|  | Very good/somewhat good | 0.08 | (-0.13,0.28) | 0.107 |
| Relationship with father | (Ref: Very bad/somewhat bad) |  |  |  |
|  | Very good/somewhat good | 0.03 | (-0.13,0.18) | 0.079 |
| Parent marital status | (Ref: Parents married) |  |  |  |
|  | No, divorced | 0.09 | (-0.09,0.27) | 0.091 |
|  | Single, never married | 0.00 | (-0.12,0.13) | 0.063 |
|  | No, one or both of them had died | 0.06 | (-0.05,0.18) | 0.058 |
| Subjective financial status of family growing up | (Ref: Got by) |  |  |  |
|  | Lived comfortably | -0.03 | (-0.09,0.03) | 0.032 |
|  | Found it difficult | -0.07 | (-0.15,0.01) | 0.041 |
|  | Found it very difficult | -0.20 | (-0.30,-0.10) | 0.051 |
| Abuse | (Ref: No) |  |  |  |
|  | Yes | -0.18 | (-0.27,-0.09) | 0.047 |
| Outsider growing up | (Ref: No) |  |  |  |
|  | Yes | -0.17 | (-0.25,-0.08) | 0.043 |
| Self-rated health growing up | (Ref: Good) |  |  |  |
|  | Excellent | 0.34 | (0.25,0.42) | 0.042 |
|  | Very good | 0.18 | (0.11,0.25) | 0.034 |
|  | Fair | -0.13 | (-0.21,-0.04) | 0.045 |
|  | Poor | -0.23 | (-0.43,-0.04) | 0.099 |
| Immigration status | (Ref: Born in this country) |  |  |  |
|  | Born in another country | -0.21 | (-0.47,0.05) | 0.133 |
| Age 12 religious service attendance | (Ref: Never) |  |  |  |
|  | At least 1/week | 0.28 | (0.18,0.37) | 0.048 |
|  | 1-3/month | 0.16 | (0.05,0.26) | 0.053 |
|  | <1/month | 0.06 | (-0.04,0.16) | 0.052 |
| Year of birth | (Ref: 1998-2005; age 18-24) |  |  |  |
|  | 1993-1998; age 25-29 | 0.07 | (-0.03,0.16) | 0.049 |
|  | 1983-1993; age 30-39 | 0.04 | (-0.05,0.13) | 0.045 |
|  | 1973-1983; age 40-49 | 0.02 | (-0.07,0.12) | 0.048 |
|  | 1963-1973; age 50-59 | 0.05 | (-0.05,0.16) | 0.054 |
|  | 1953-1963; age 60-69 | -0.06 | (-0.18,0.06) | 0.061 |
|  | 1943 or earlier; age 80+ | 0.06 | (-0.36,0.47) | 0.211 |
|  | 1943-1953; age 70-79 | -0.23 | (-0.43,-0.04) | 0.100 |
| Gender | (Ref: Male) |  |  |  |
|  | Female | 0.05 | (0.00,0.10) | 0.025 |
|  | Other | -0.81 | (-1.19,-0.42) | 0.198 |

*Table S24. Population weighted meta-analysis of E-values.*

| Variable | Category | evalue | evalue.limit |
| --- | --- | --- | --- |
| Relationship with mother | (Ref: Very bad/somewhat bad) |  |  |
|  | Very good/somewhat good | 1.22 | 1.00 |
| Relationship with father | (Ref: Very bad/somewhat bad) |  |  |
|  | Very good/somewhat good | 1.12 | 1.00 |
| Parent marital status | (Ref: Parents married) |  |  |
|  | No, divorced | 1.25 | 1.00 |
|  | Single, never married | 1.04 | 1.00 |
|  | No, one or both of them had died | 1.20 | 1.00 |
| Subjective financial status of family growing up | (Ref: Got by) |  |  |
|  | Lived comfortably | 1.13 | 1.00 |
|  | Found it difficult | 1.21 | 1.00 |
|  | Found it very difficult | 1.41 | 1.27 |
| Abuse | (Ref: No) |  |  |
|  | Yes | 1.38 | 1.25 |
| Outsider growing up | (Ref: No) |  |  |
|  | Yes | 1.36 | 1.23 |
| Self-rated health growing up | (Ref: Good) |  |  |
|  | Excellent | 1.59 | 1.48 |
|  | Very good | 1.38 | 1.28 |
|  | Fair | 1.30 | 1.15 |
|  | Poor | 1.45 | 1.15 |
| Immigration status | (Ref: Born in this country) |  |  |
|  | Born in another country | 1.42 | 1.00 |
| Age 12 religious service attendance | (Ref: Never) |  |  |
|  | At least 1/week | 1.51 | 1.39 |
|  | 1-3/month | 1.35 | 1.17 |
|  | <1/month | 1.19 | 1.00 |
| Year of birth | (Ref: 1998-2005; age 18-24) |  |  |
|  | 1993-1998; age 25-29 | 1.20 | 1.00 |
|  | 1983-1993; age 30-39 | 1.15 | 1.00 |
|  | 1973-1983; age 40-49 | 1.11 | 1.00 |
|  | 1963-1973; age 50-59 | 1.18 | 1.00 |
|  | 1953-1963; age 60-69 | 1.19 | 1.00 |
|  | 1943 or earlier; age 80+ | 1.18 | 1.00 |
|  | 1943-1953; age 70-79 | 1.46 | 1.15 |
| Gender | (Ref: Male) |  |  |
|  | Female | 1.18 | 1.04 |
|  | Other | 2.20 | 1.69 |

# Forest Plots


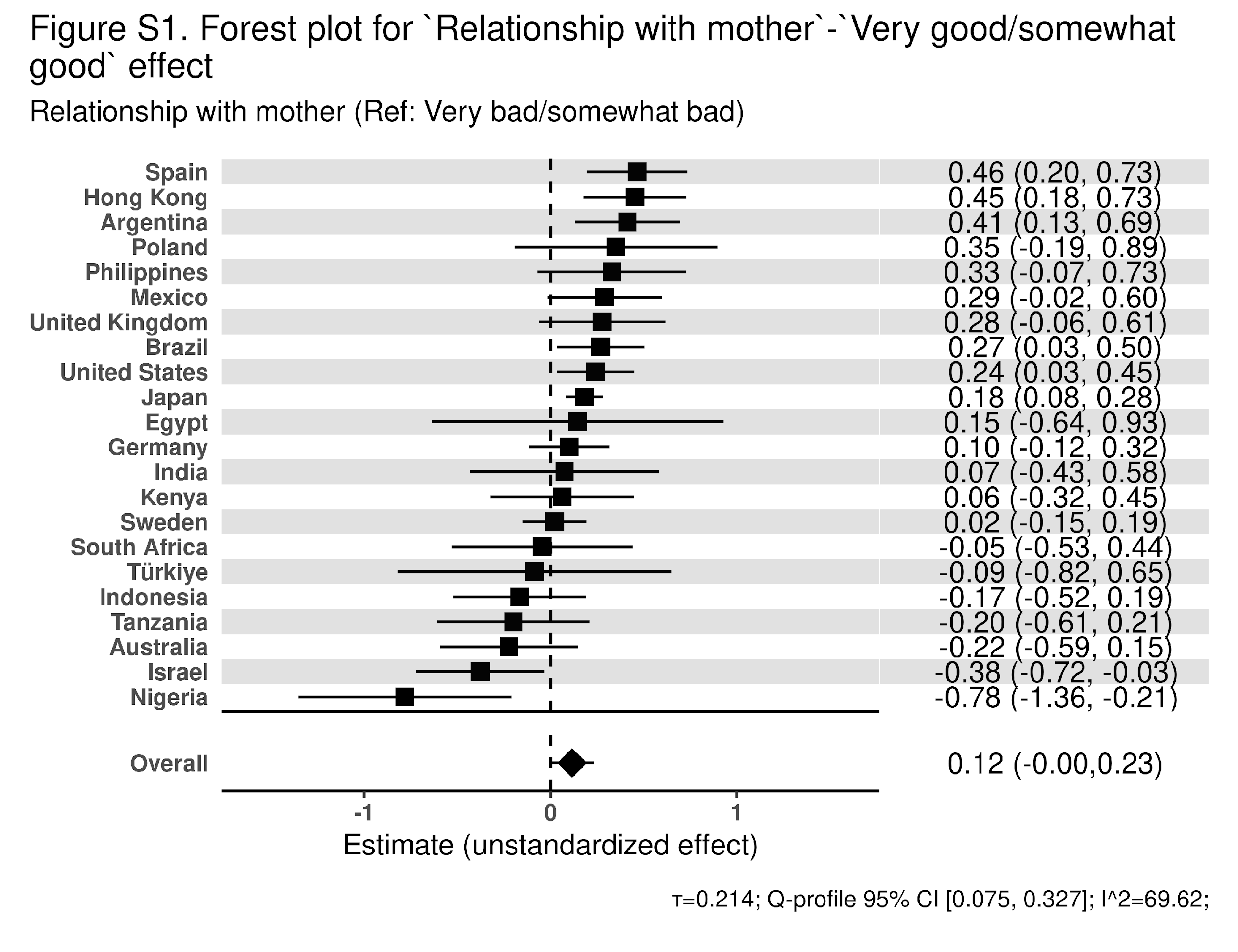

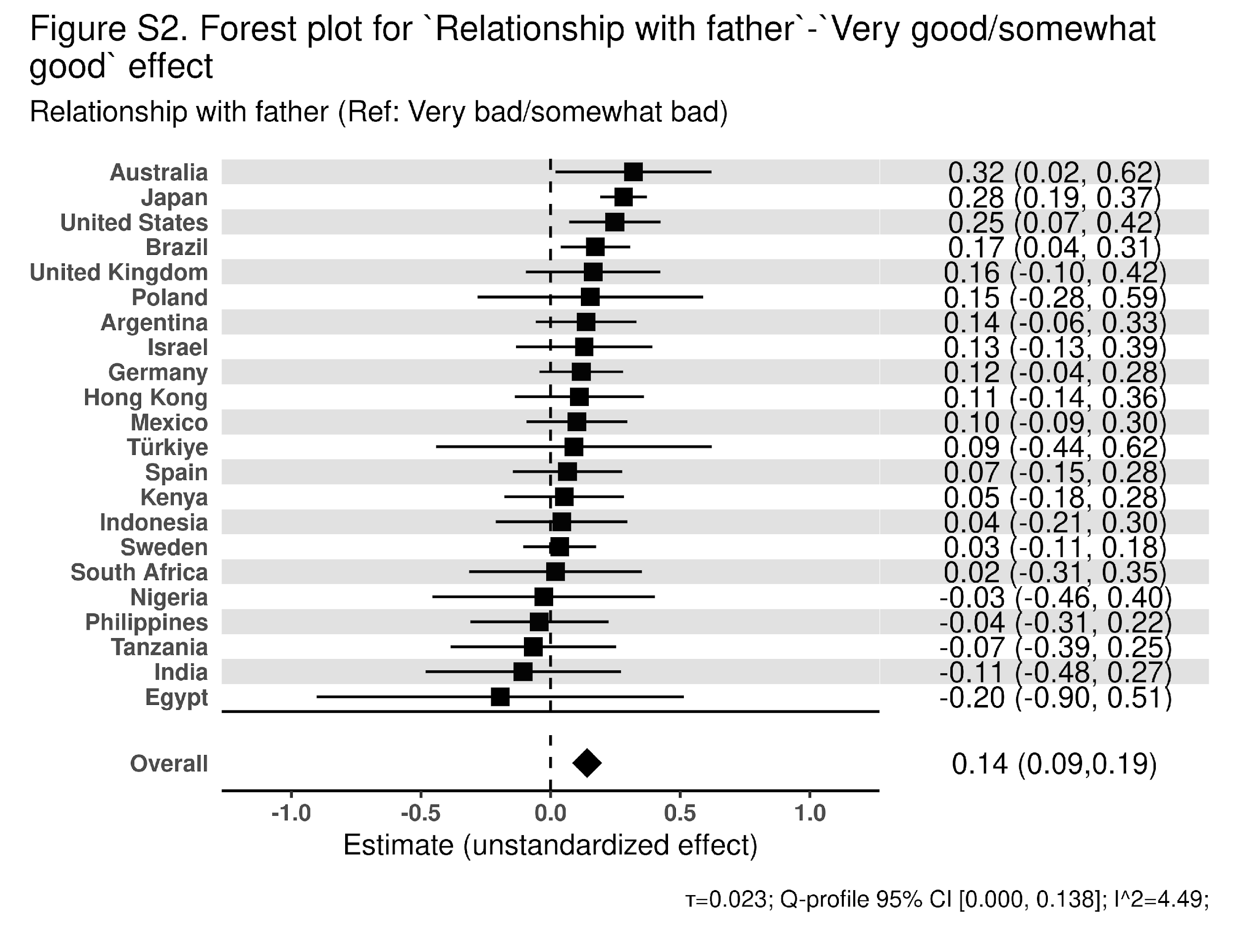

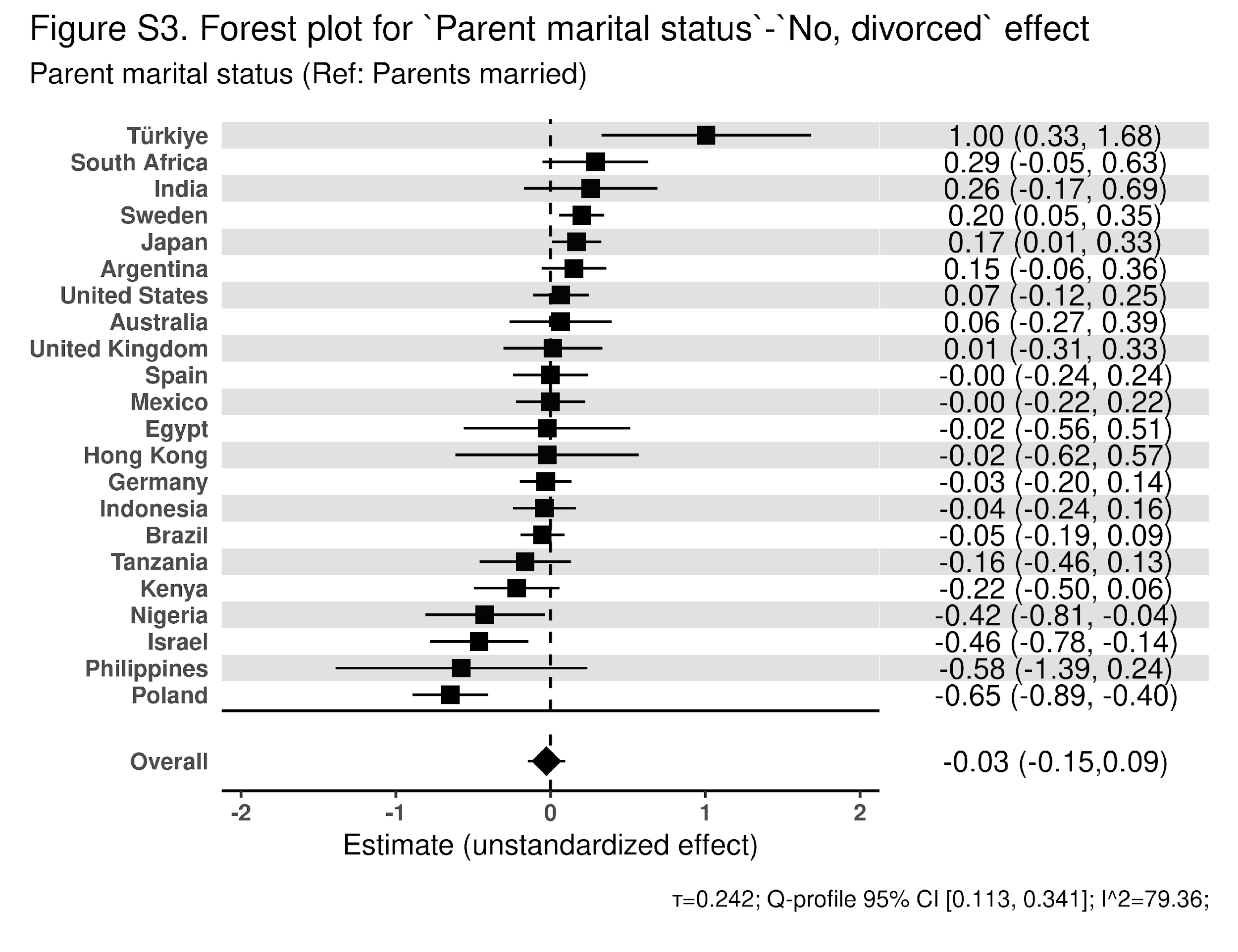

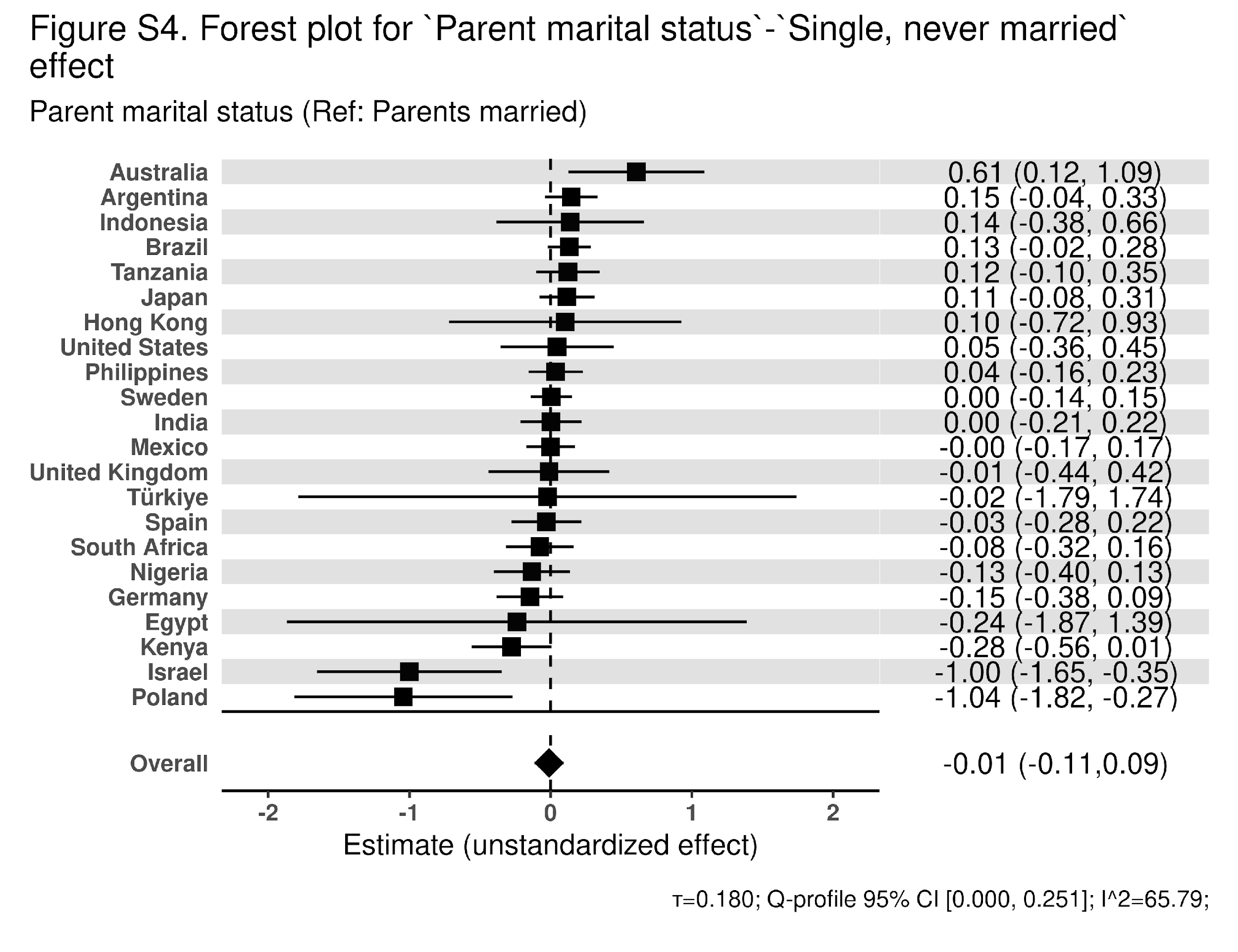

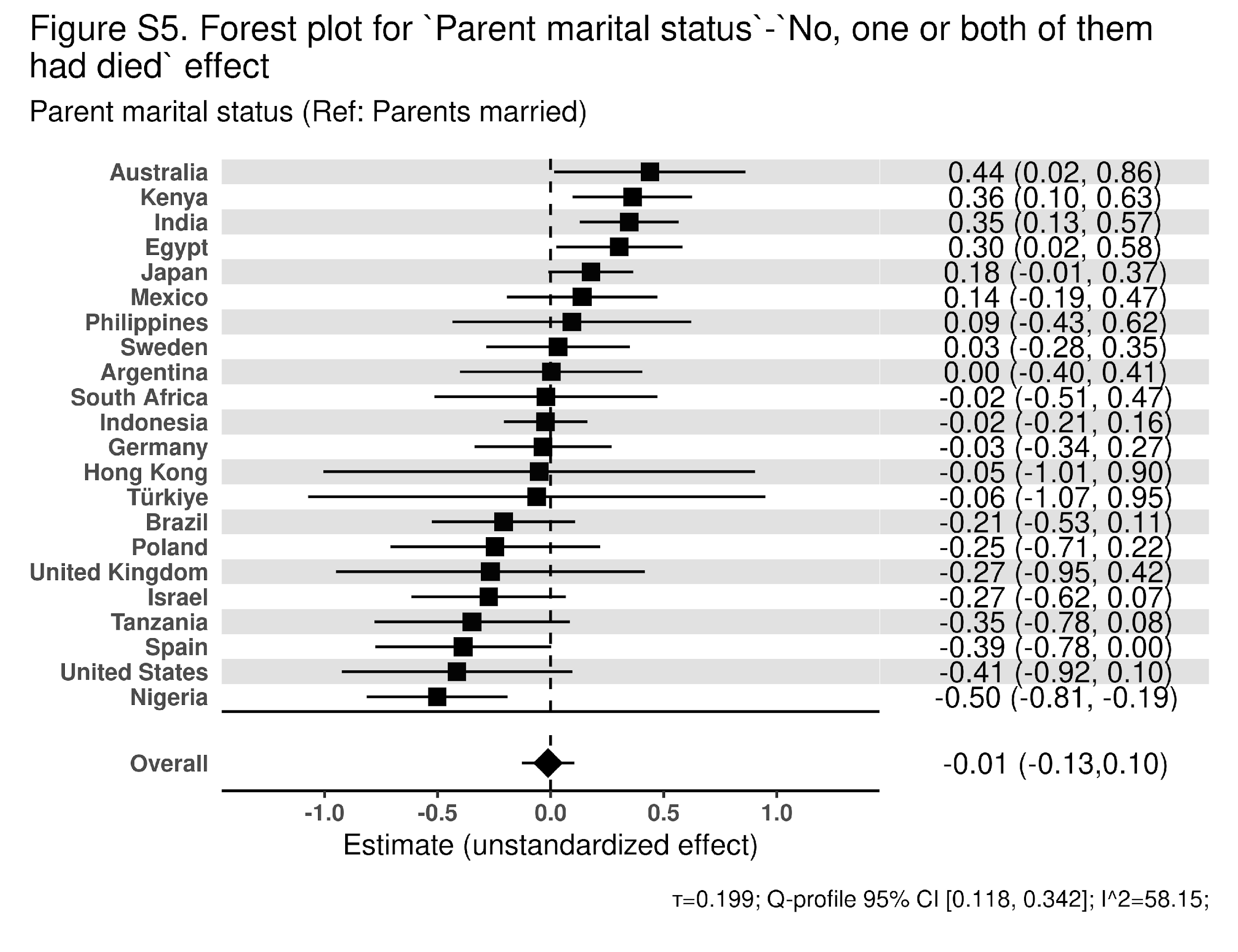

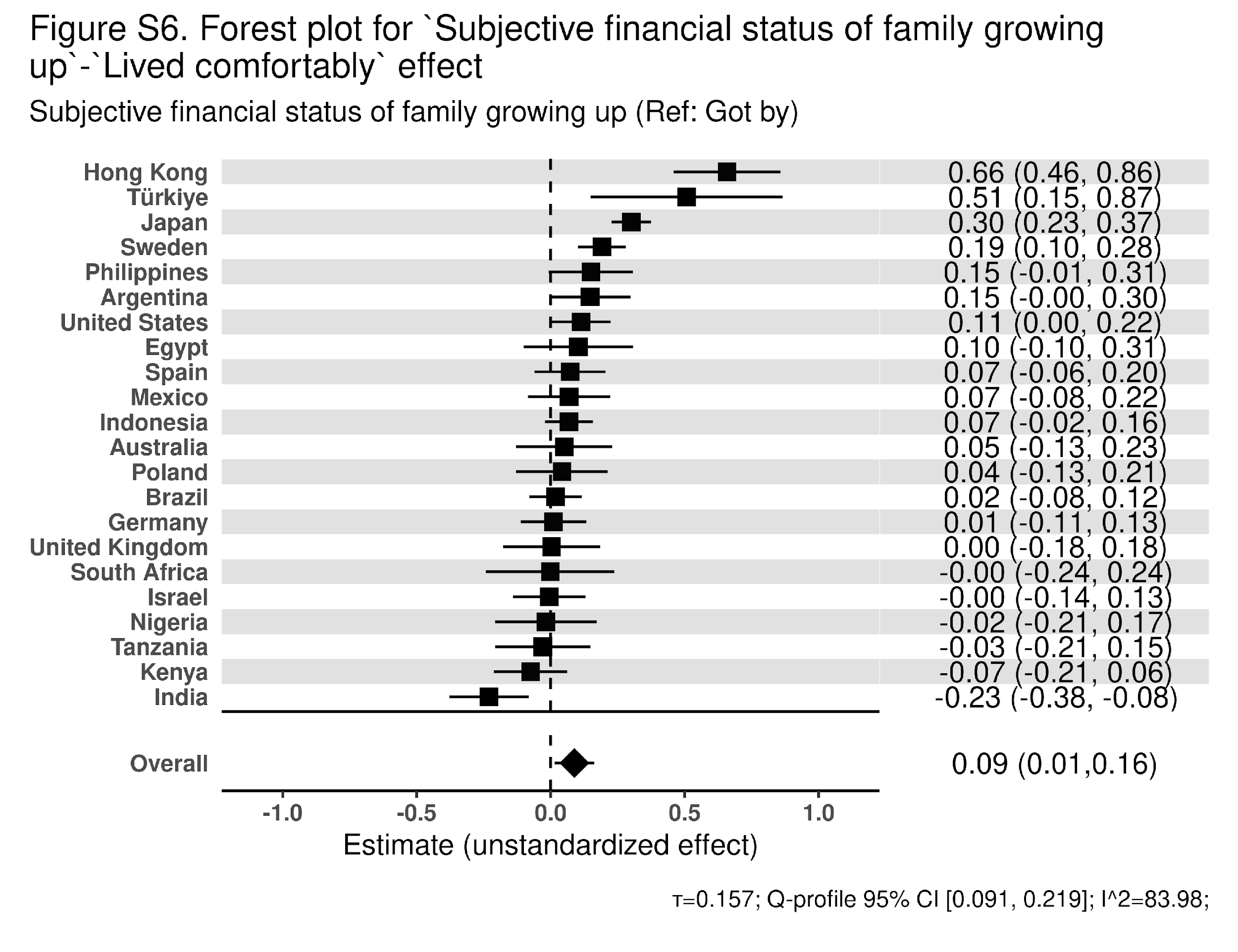

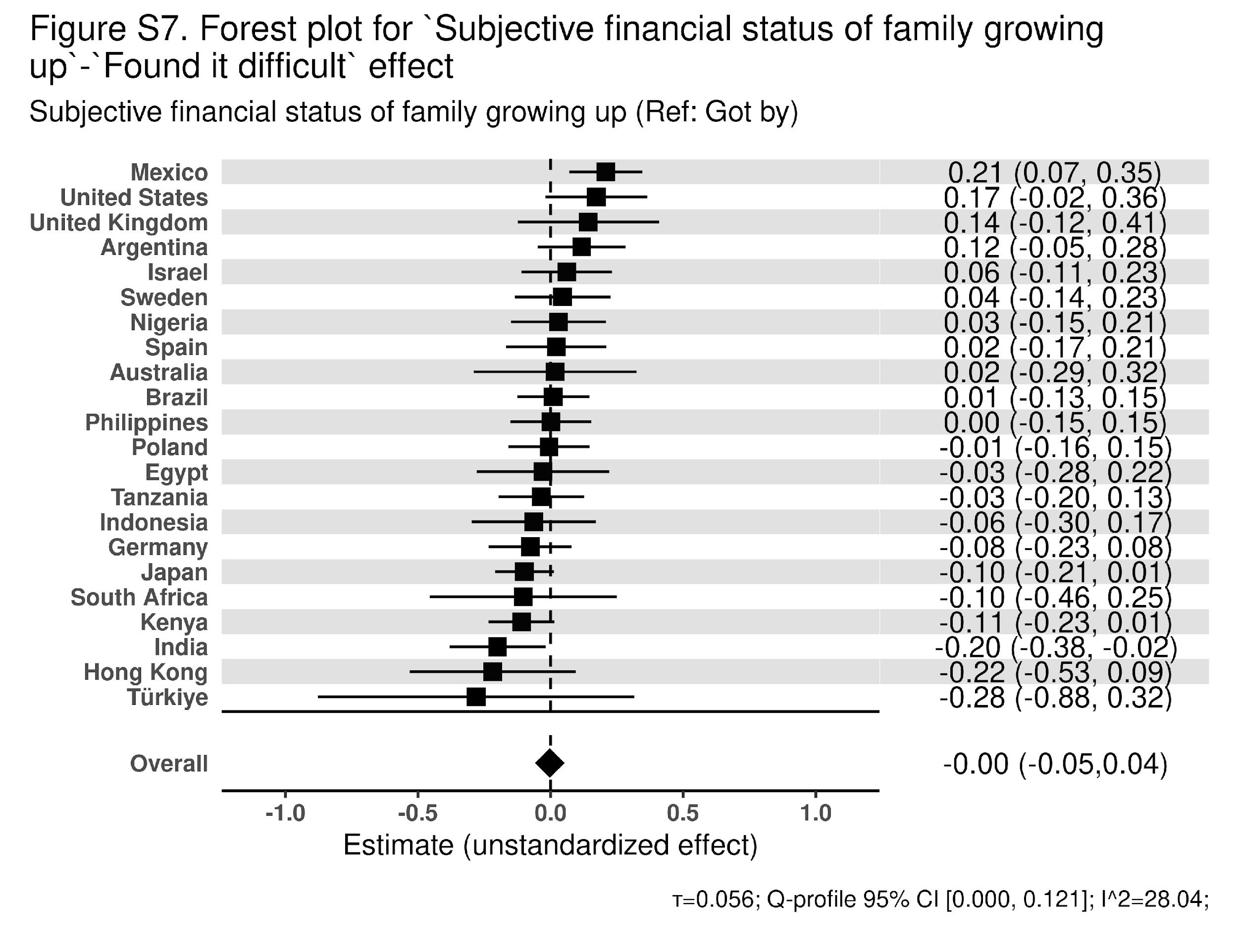

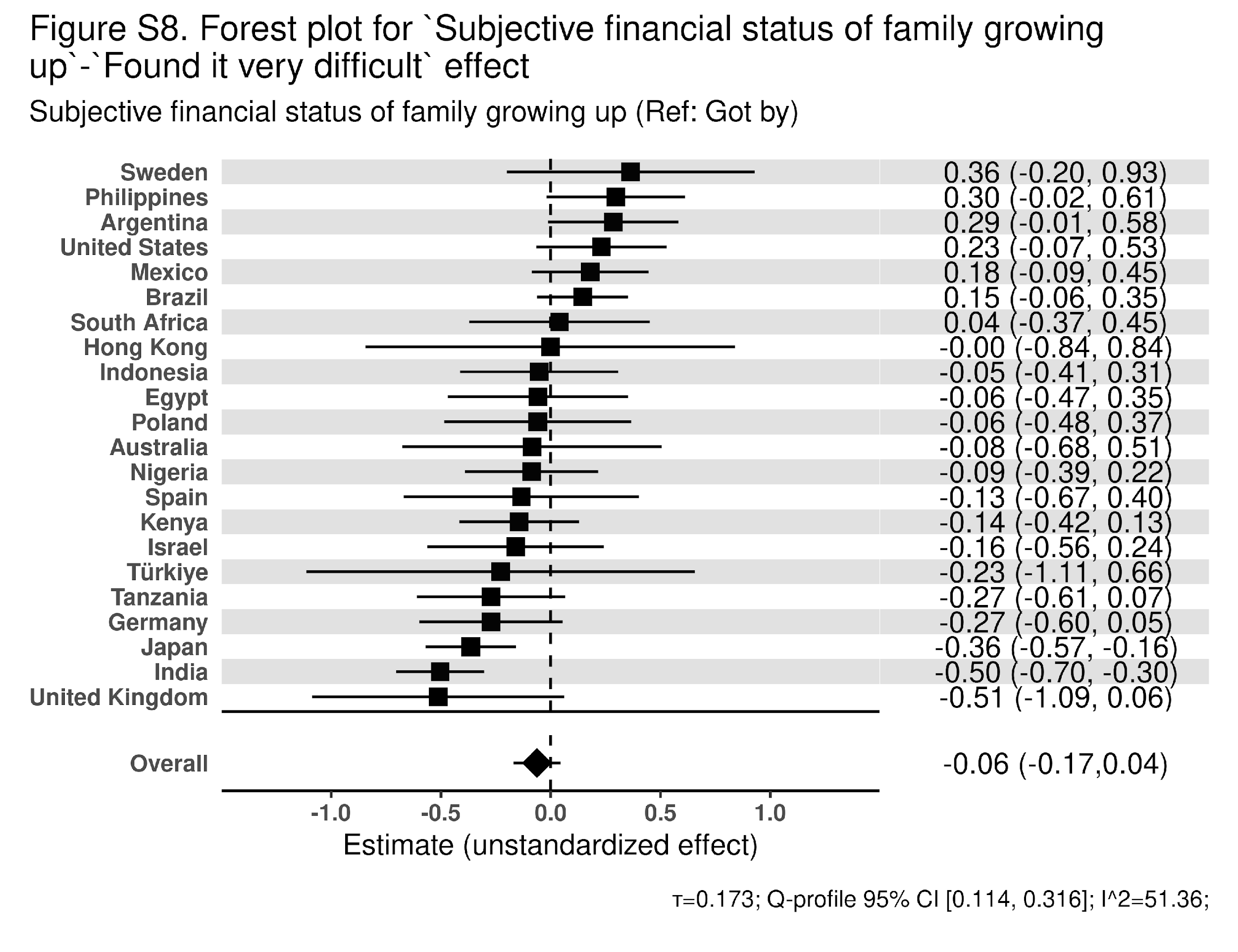

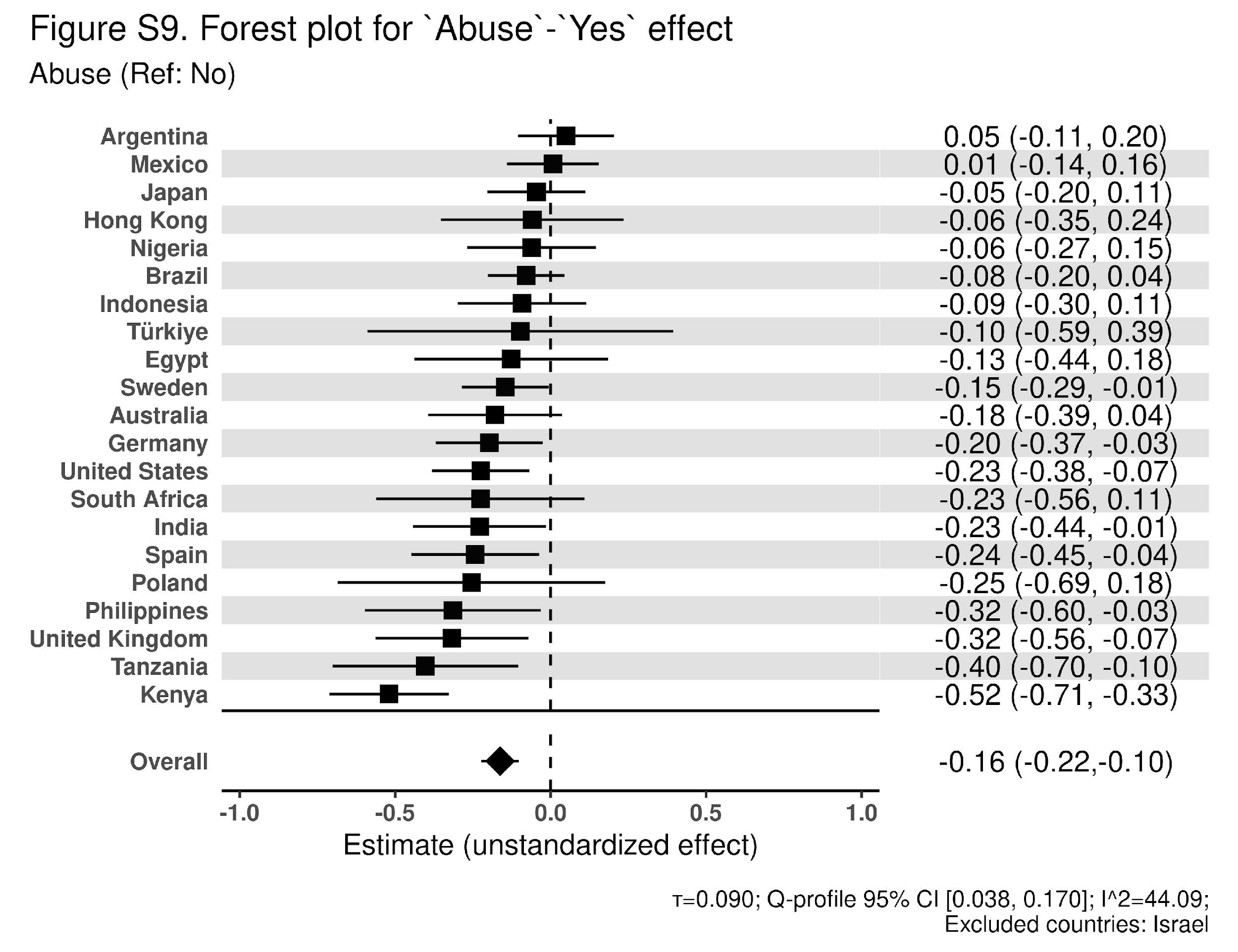

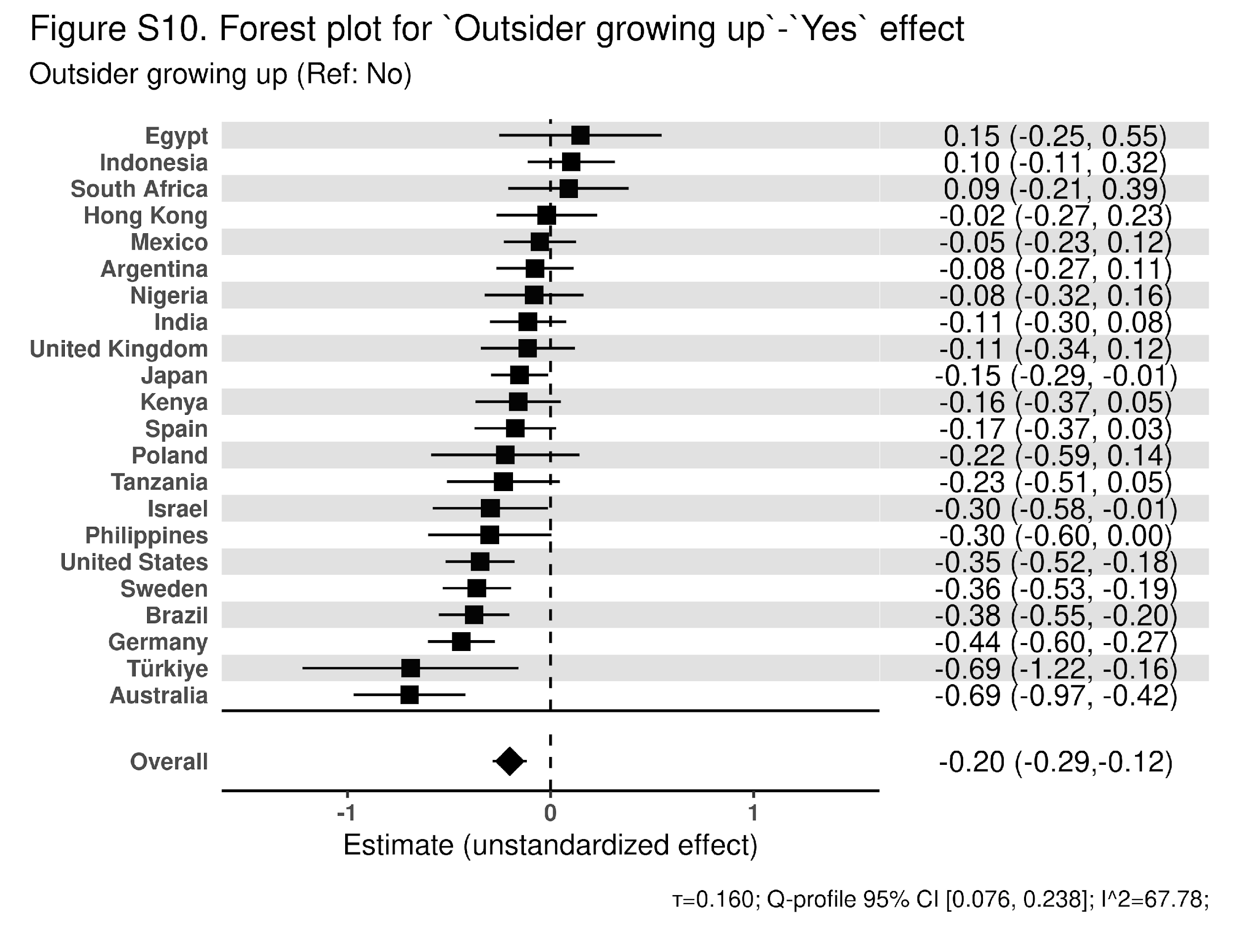

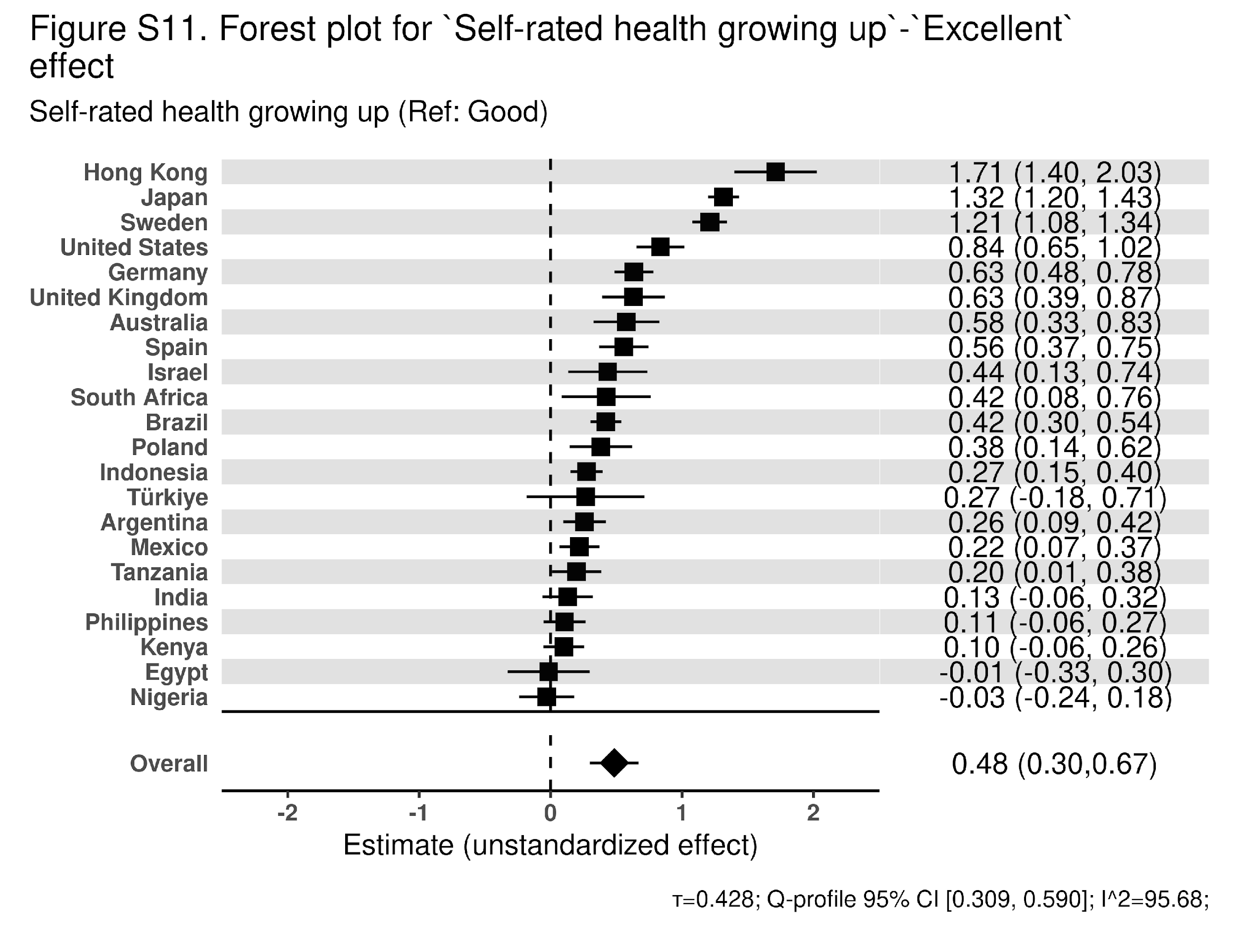

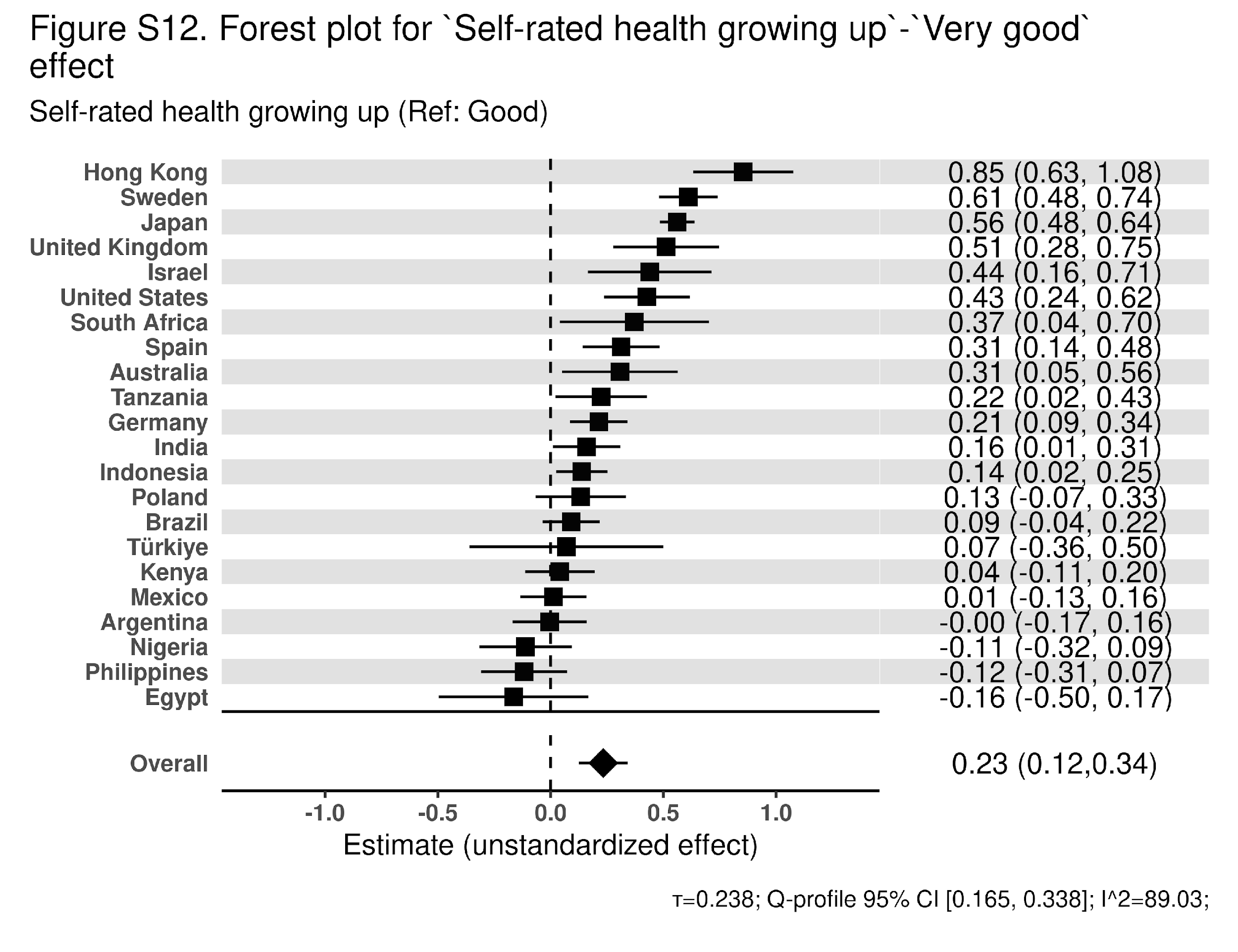

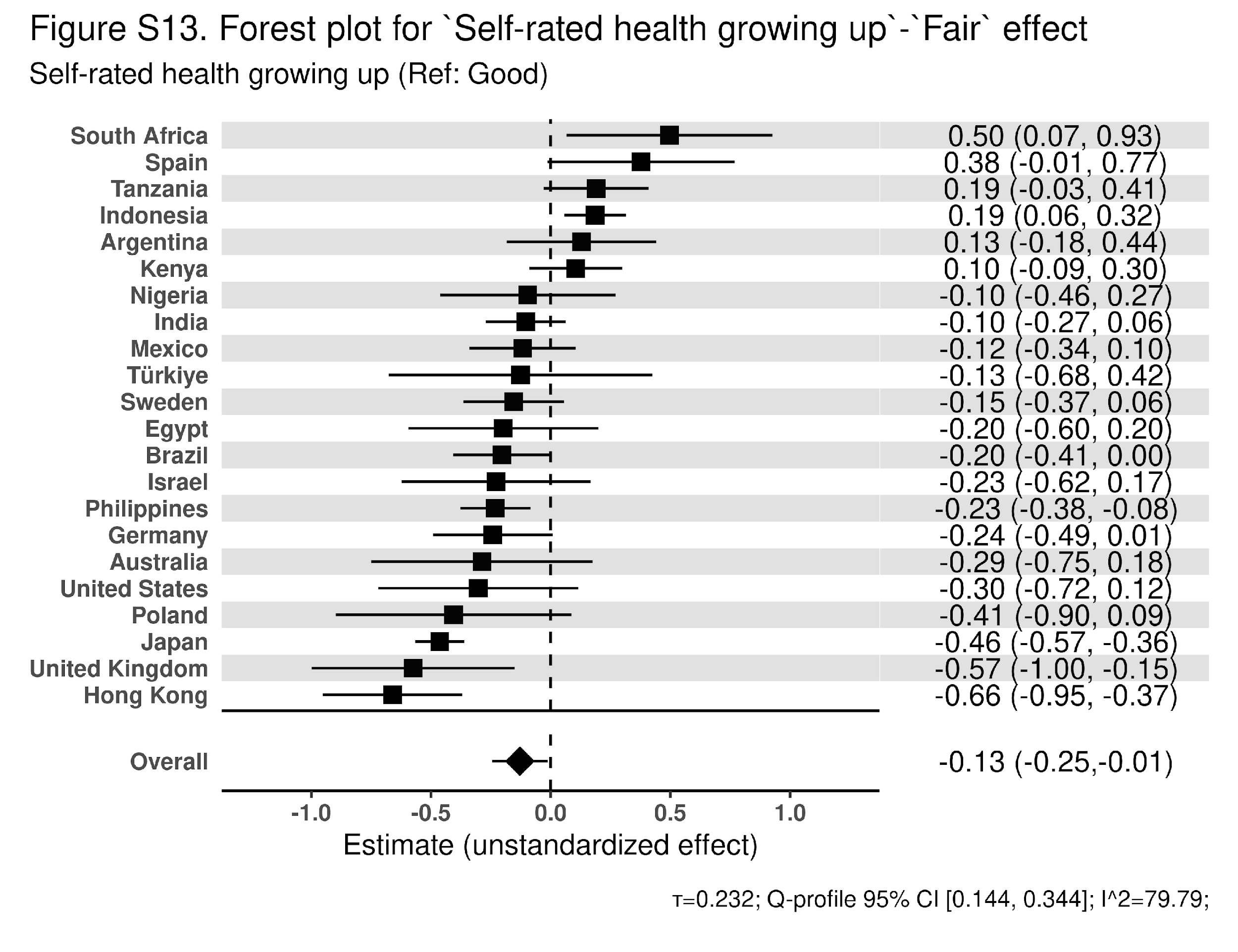

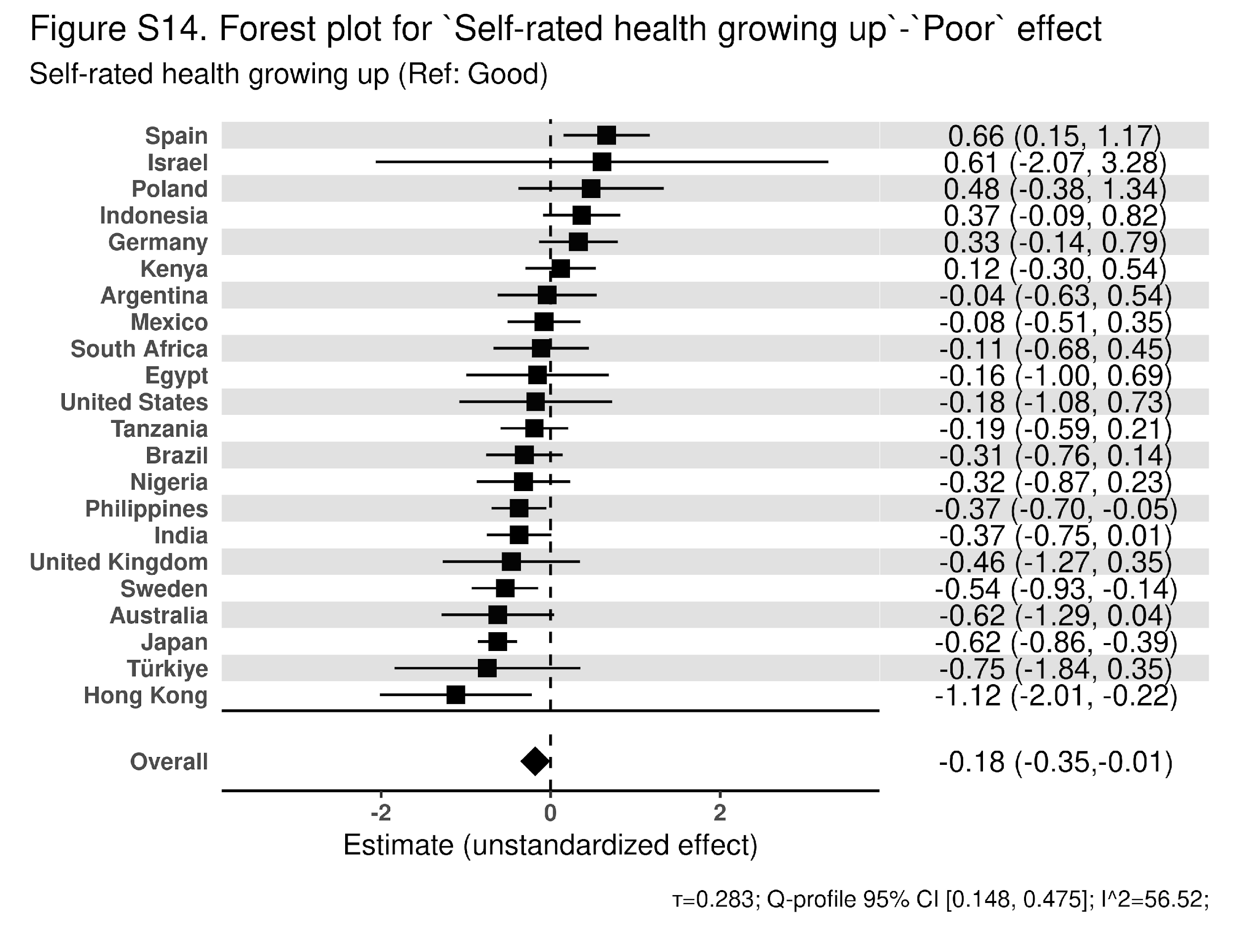

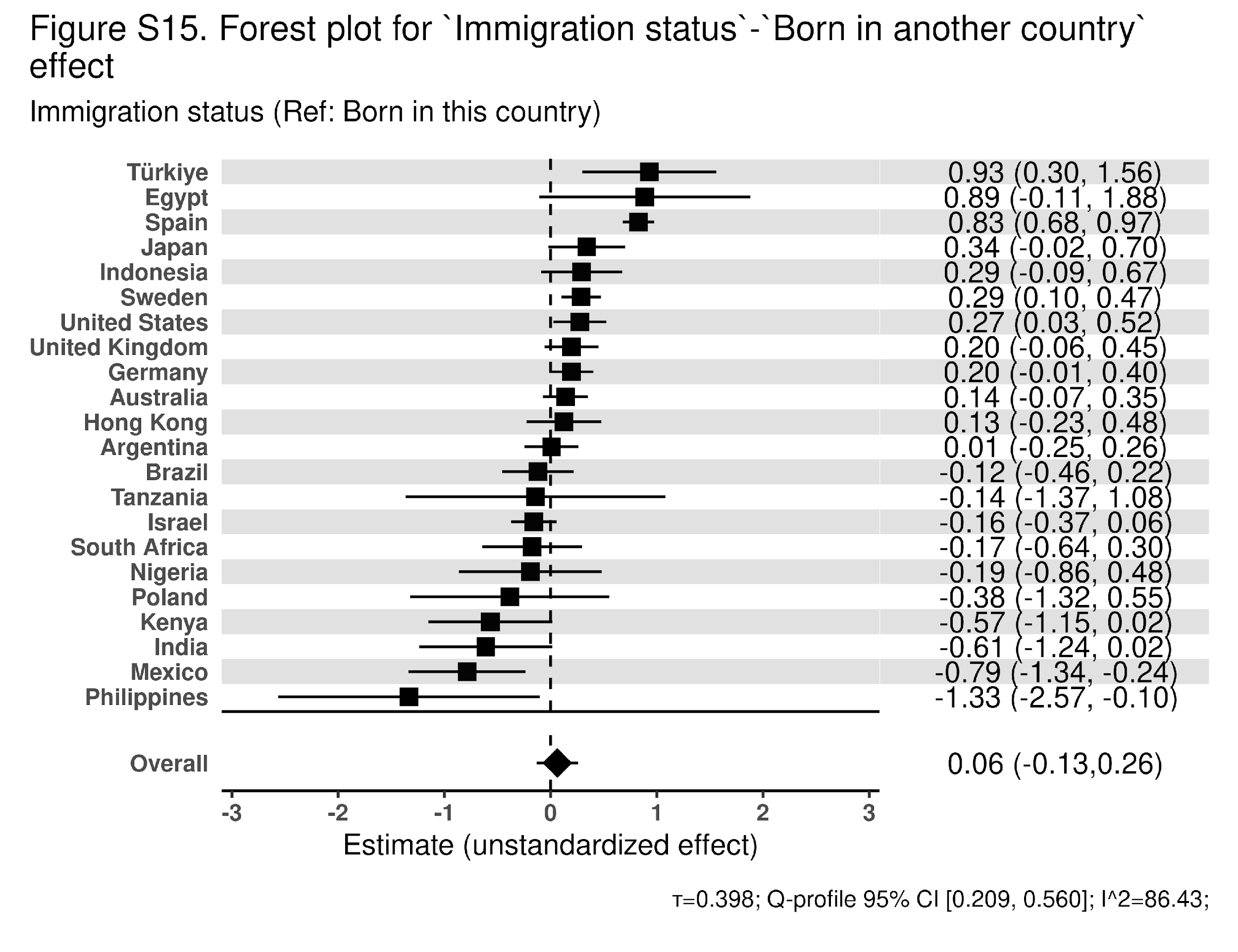

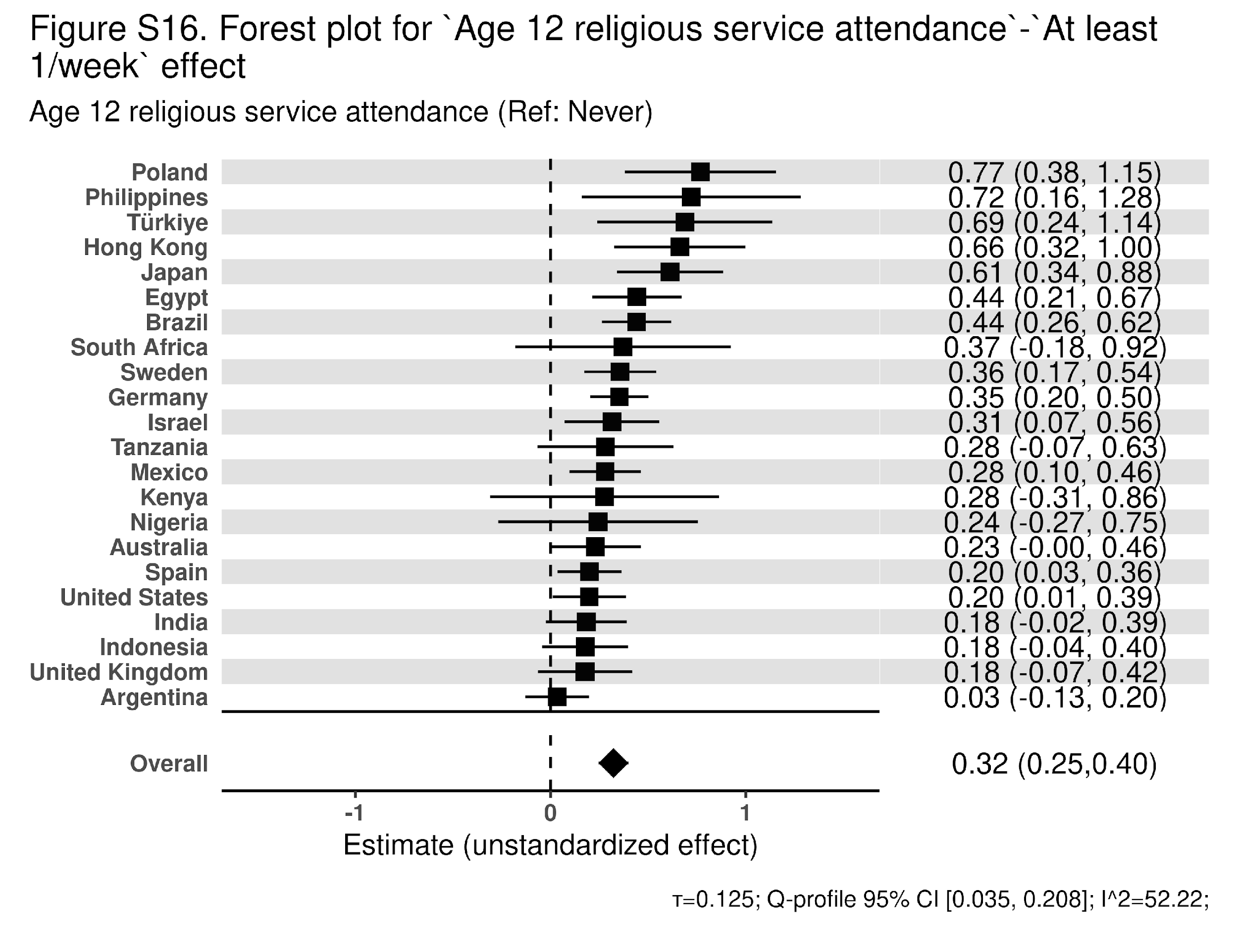

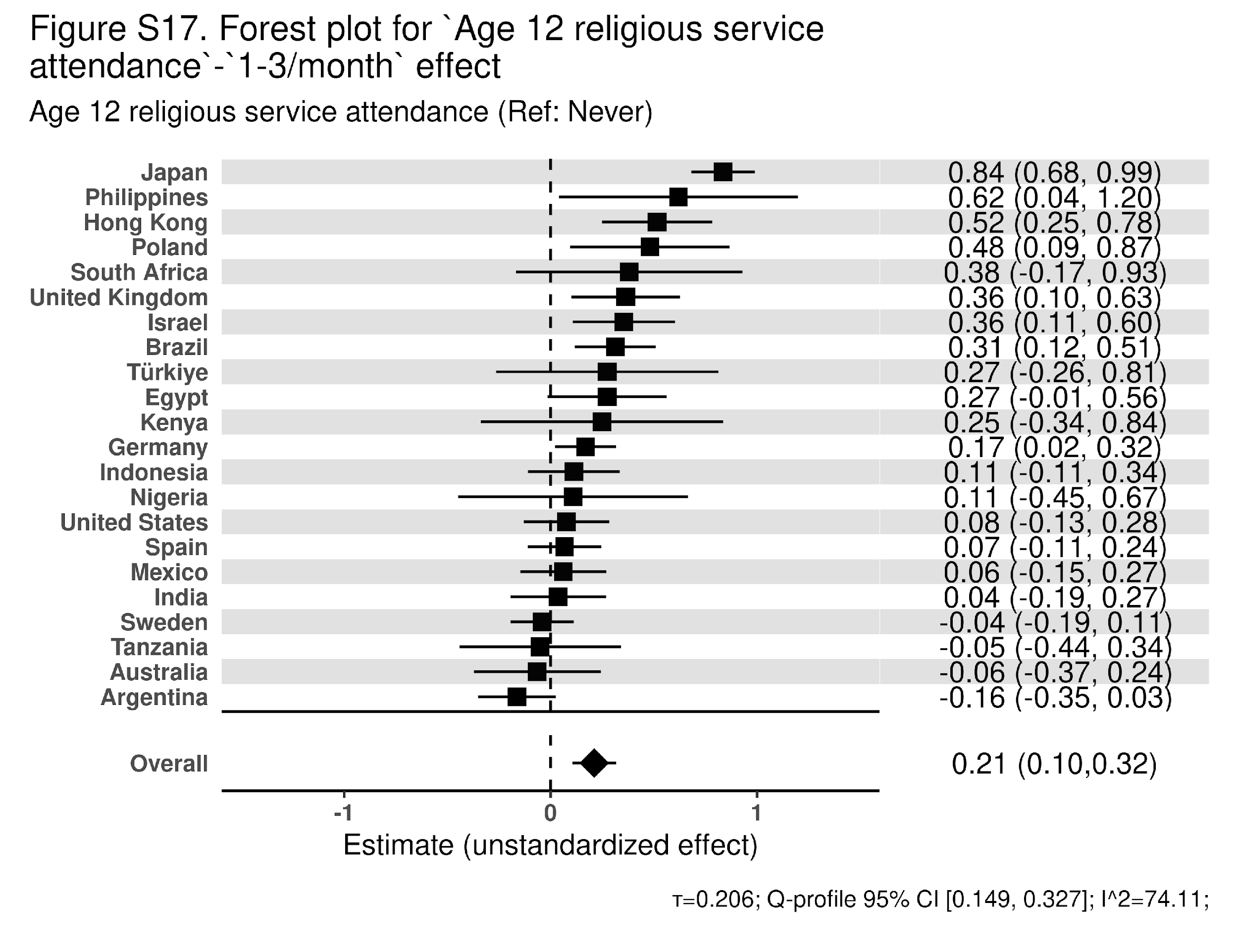

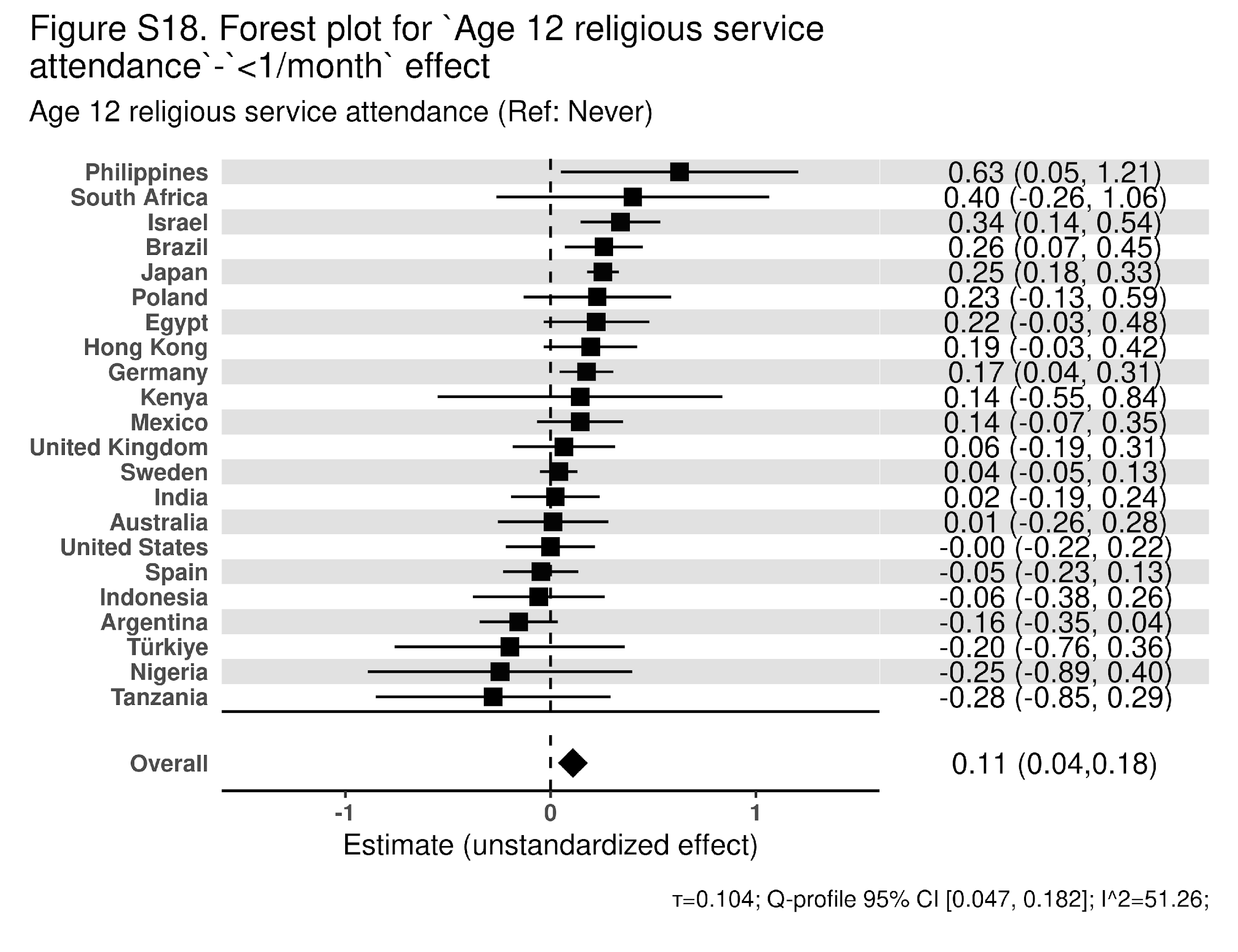

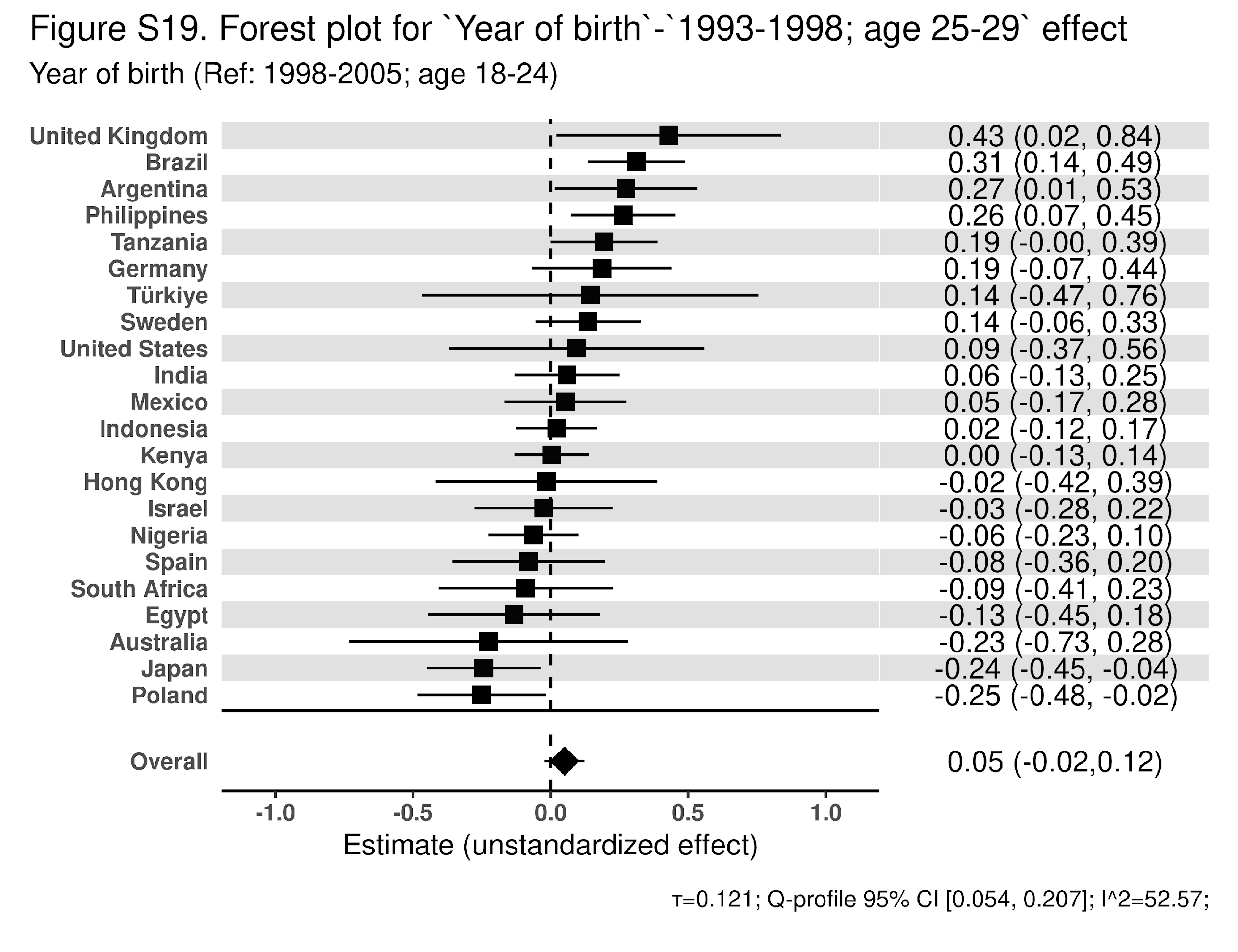

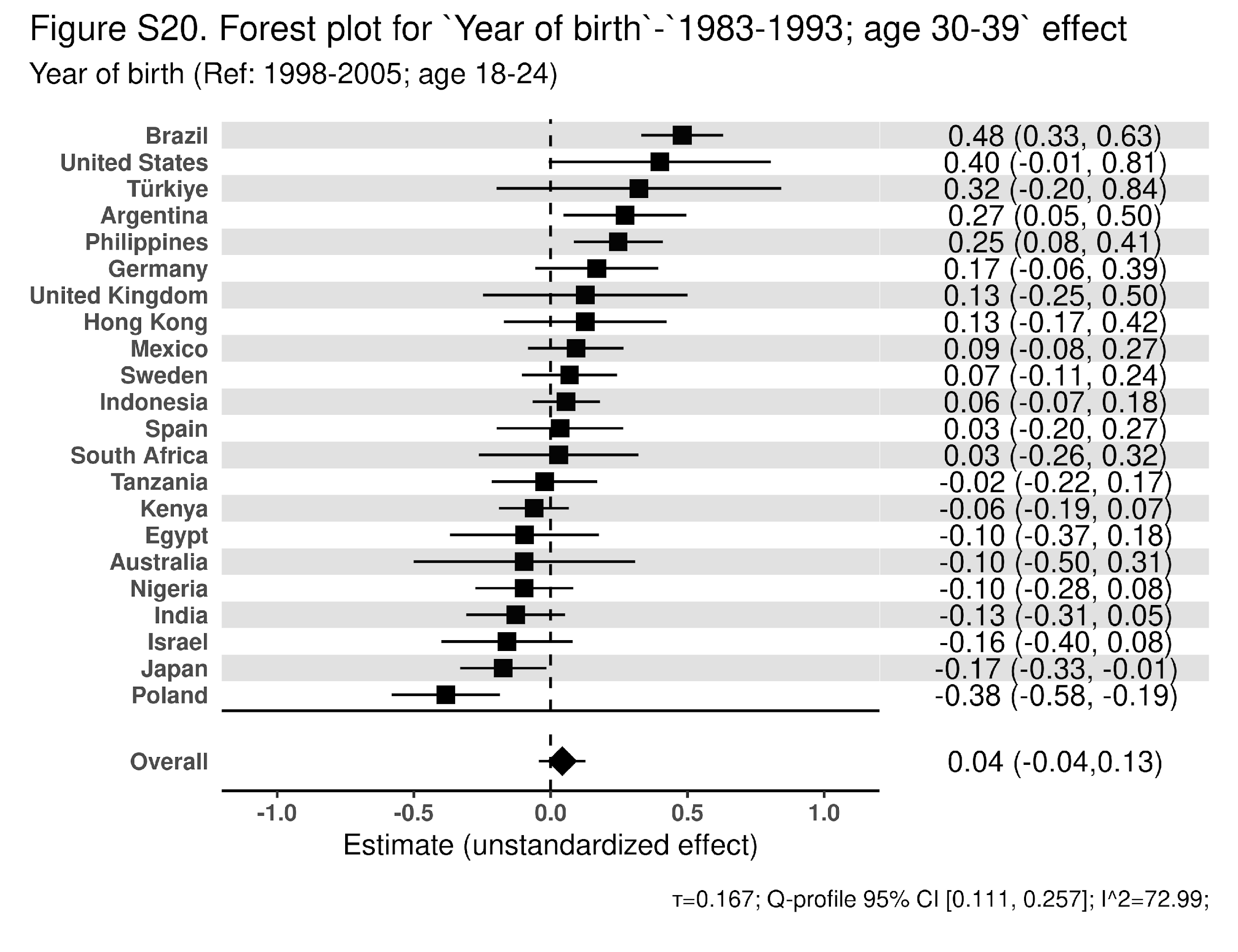

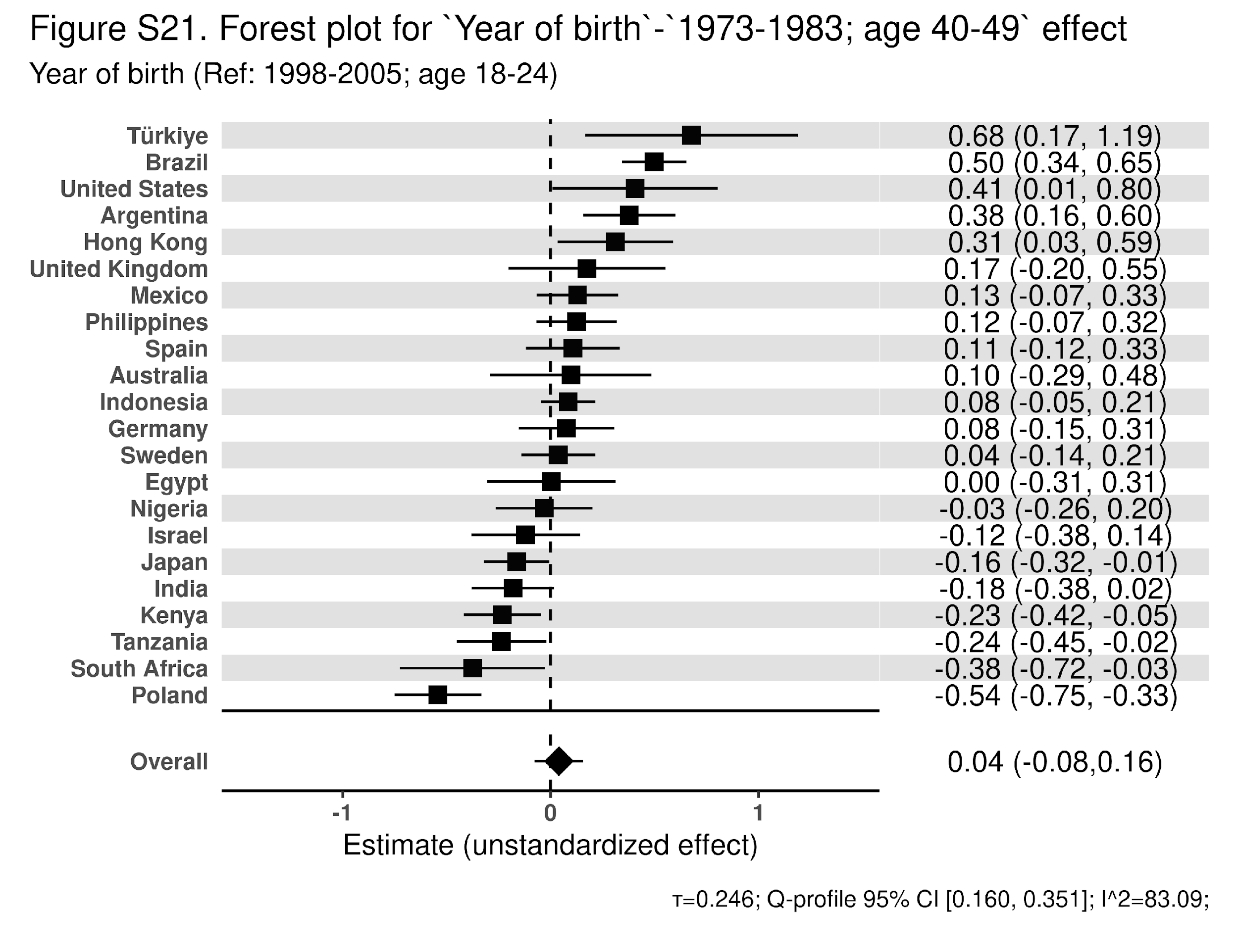

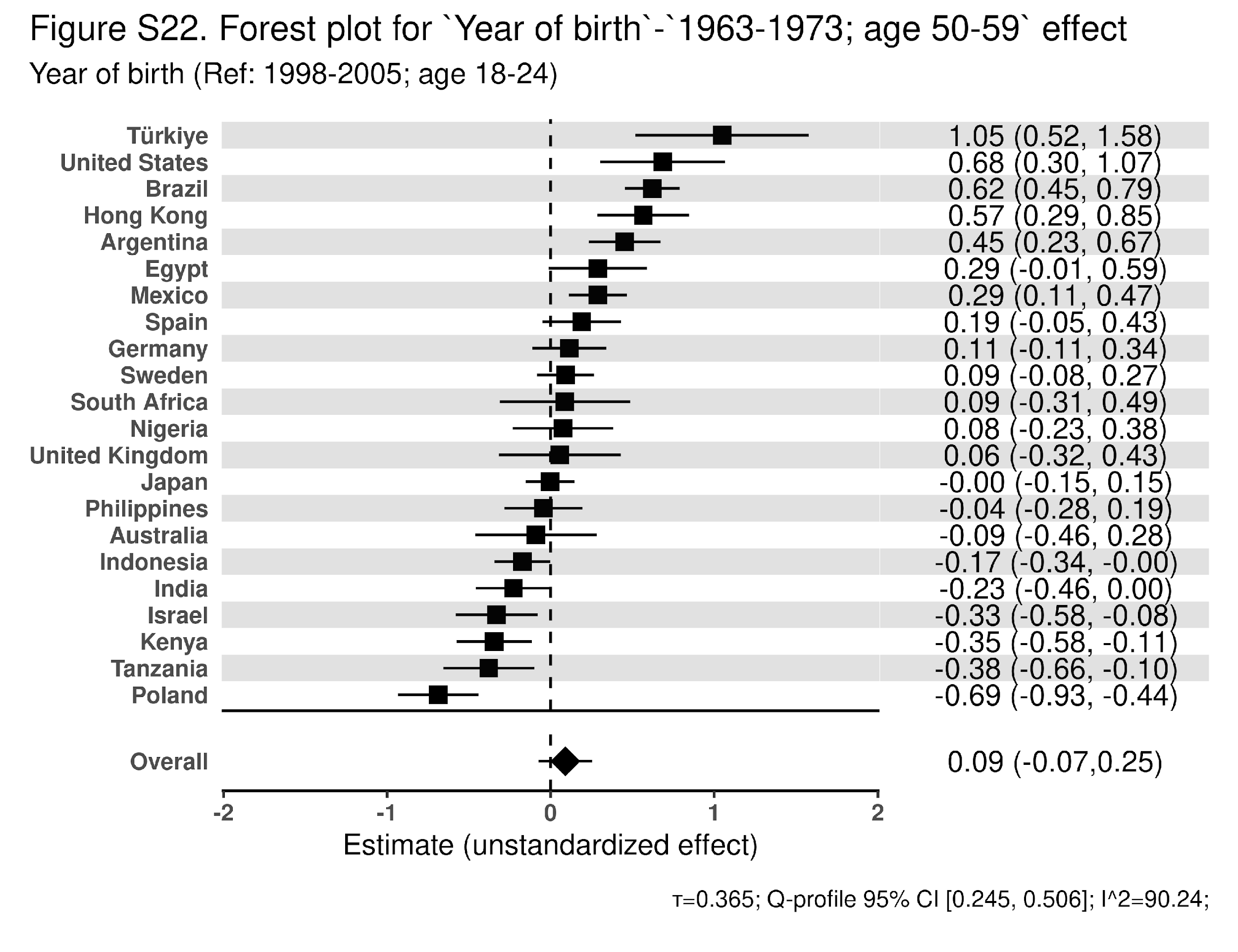

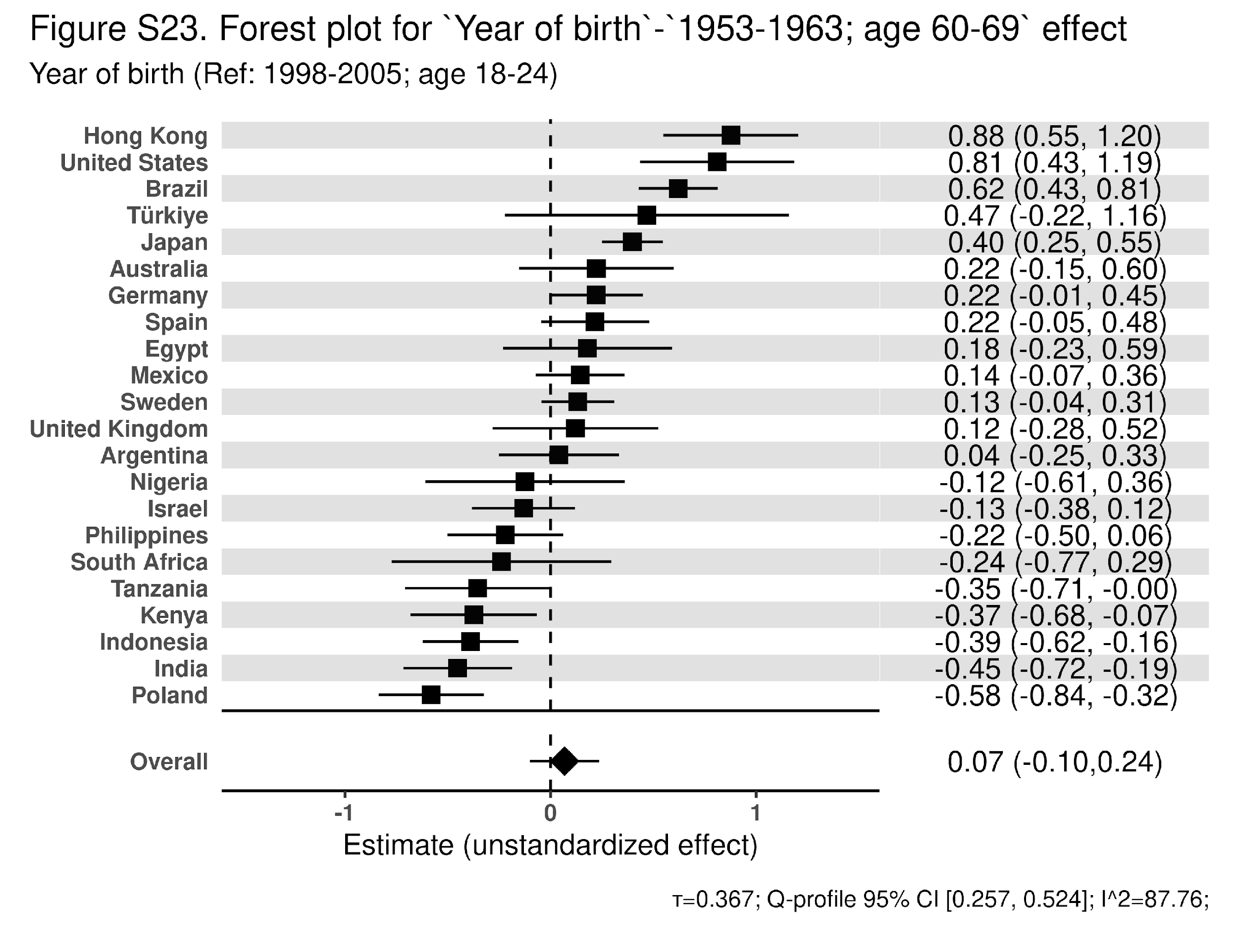

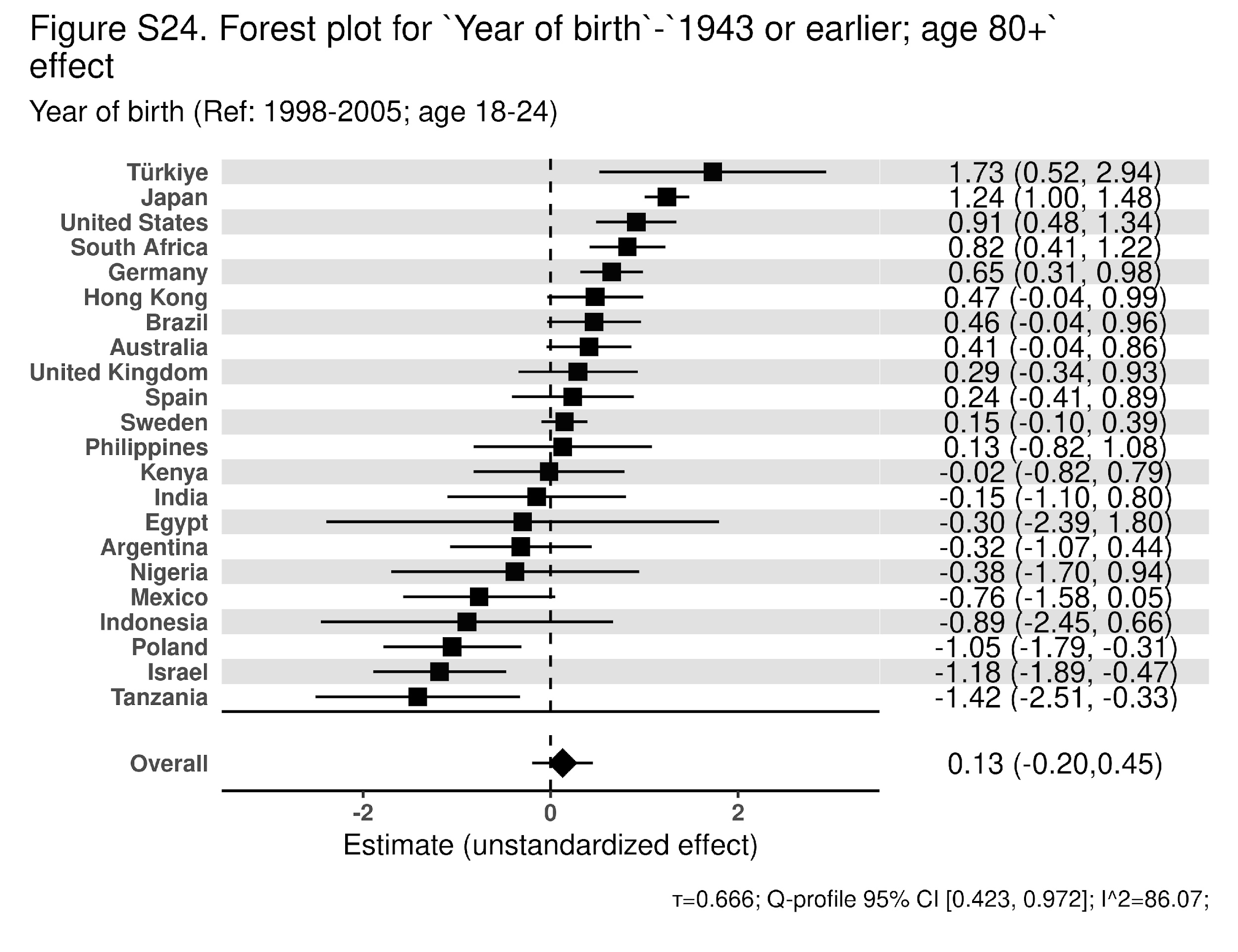

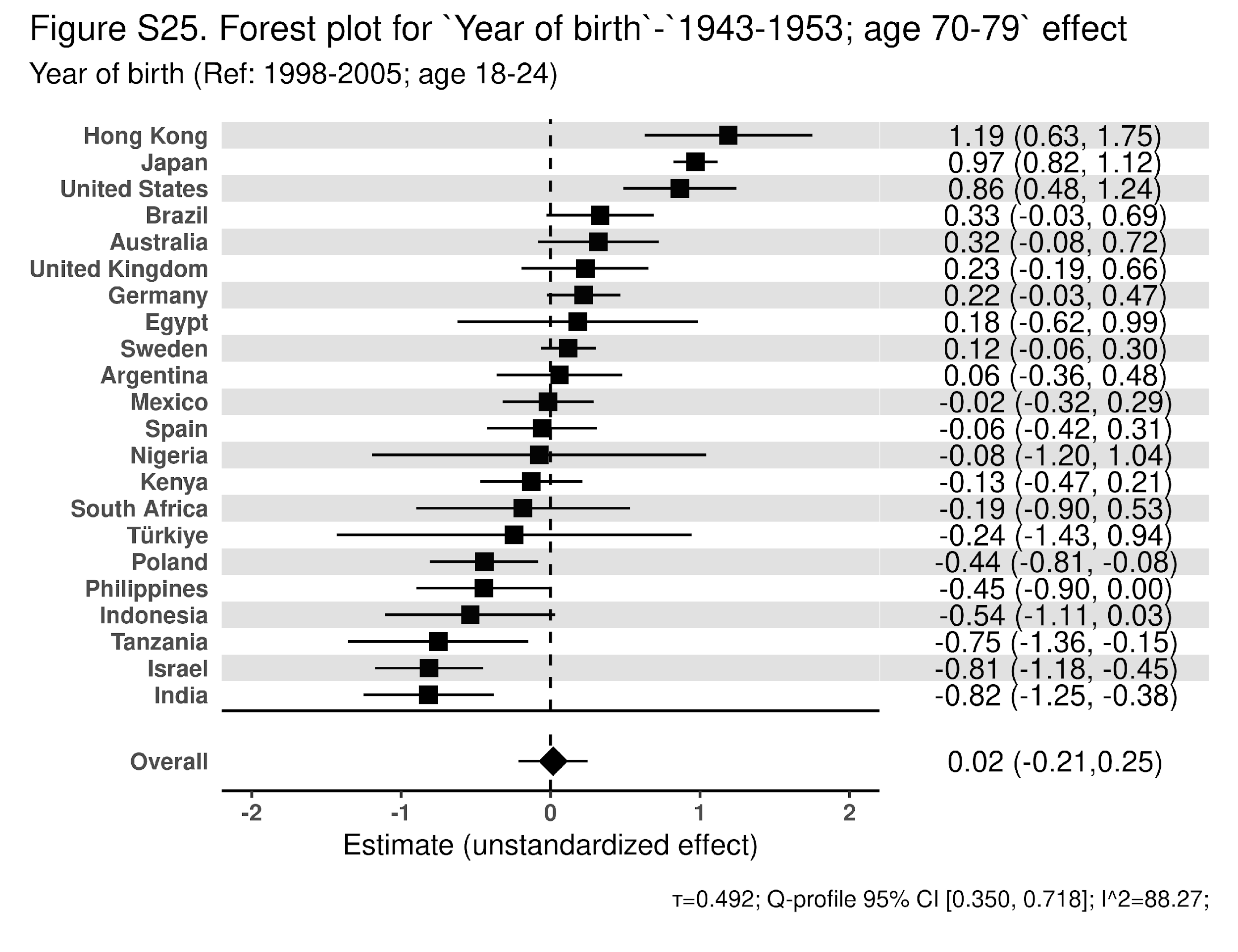

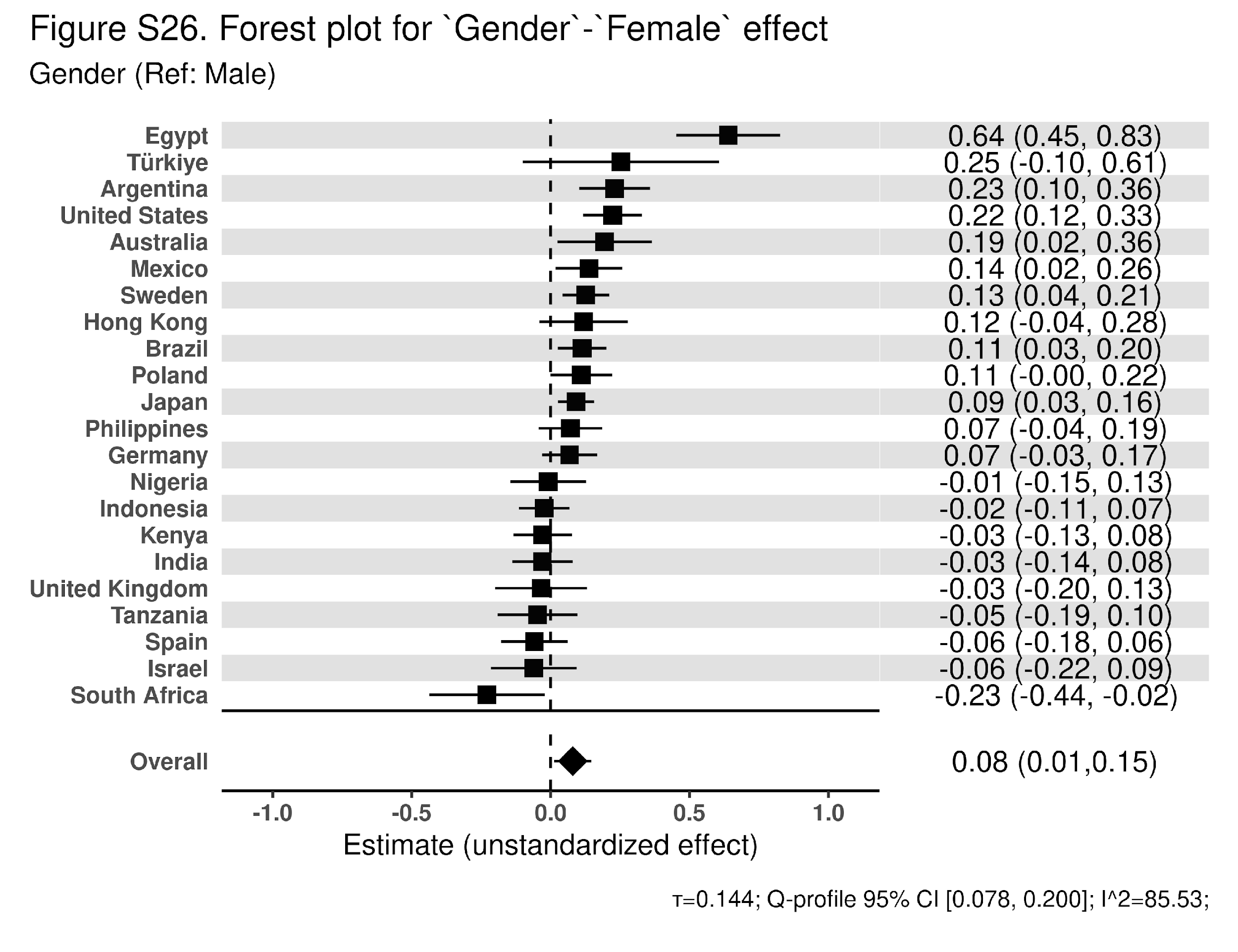

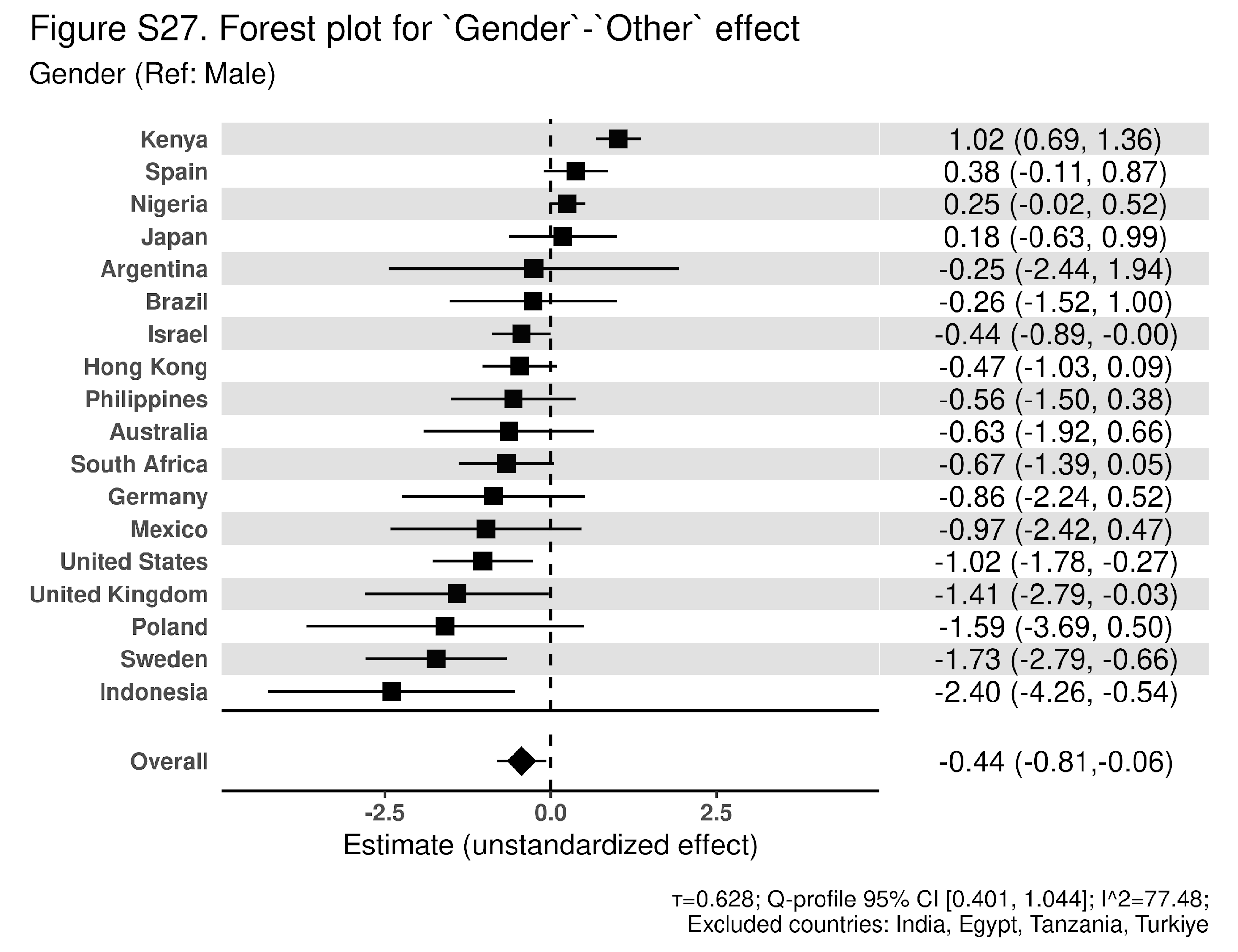


***Table S1a. Nationally representative descriptive statistics for Argentina***

| **Characteristic** | **N = 6,724**^1^ |
| --- | --- |
| **Relationship with mother** |  |
| Very good | 4,463 (66%) |
| Somewhat good | 1,436 (21%) |
| Somewhat bad | 299 (4.4%) |
| Very bad | 216 (3.2%) |
| Does not apply | 273 (4.1%) |
| Missing | 36 (0.5%) |
| **Relationship with father** |  |
| Very good | 3,612 (54%) |
| Somewhat good | 1,537 (23%) |
| Somewhat bad | 440 (6.5%) |
| Very bad | 401 (6.0%) |
| Does not apply | 694 (10%) |
| Missing | 39 (0.6%) |
| **Parent marital status** |  |
| Yes, married | 4,110 (61%) |
| No, divorced | 637 (9.5%) |
| Never married | 1,368 (20%) |
| No, one or both of them had died | 199 (3.0%) |
| Missing | 410 (6.1%) |
| **Subjective financial status of family growing up** |  |
| Lived comfortably | 2,042 (30%) |
| Got by | 2,305 (34%) |
| Found it difficult | 1,789 (27%) |
| Found it very difficult | 569 (8.5%) |
| Missing | 19 (0.3%) |
| **Abuse** |  |
| Yes | 1,302 (19%) |
| No | 5,271 (78%) |
| Missing | 151 (2.2%) |
| **Outsider growing up** |  |
| Yes | 1,165 (17%) |
| No | 5,458 (81%) |
| Missing | 101 (1.5%) |
| **Self-rated health growing up** |  |
| Excellent | 2,402 (36%) |
| Very good | 1,819 (27%) |
| Good | 1,830 (27%) |
| Fair | 505 (7.5%) |
| Poor | 156 (2.3%) |
| Missing | 12 (0.2%) |
| **Immigration status** |  |
| Born in this country | 6,346 (94%) |
| Born in another country | 348 (5.2%) |
| Missing | 29 (0.4%) |
| **Age 12 religious service attendance** |  |
| At least 1/week | 2,601 (39%) |
| 1-3/month | 1,204 (18%) |
| <1/month | 1,059 (16%) |
| Never | 1,808 (27%) |
| Missing | 53 (0.8%) |
| **Age group** |  |
| 1998-2005; current age: 18-24 | 1,108 (16%) |
| 1993-1998; age: 25-29 | 719 (11%) |
| 1983-1993; age: 30-39 | 1,432 (21%) |
| 1973-1983; age 40-49 | 1,254 (19%) |
| 1963-1973; age 50-59 | 1,014 (15%) |
| 1953-1963; age 60-69 | 730 (11%) |
| 1943 or earlier; age 80+ | 112 (1.7%) |
| 1943-1953; age 70-79 | 356 (5.3%) |
| Missing | 0 (0%) |
| **Gender** |  |
| Male | 3,143 (47%) |
| Female | 3,542 (53%) |
| Other | 21 (0.3%) |
| Missing | 18 (0.3%) |
| **Religious affiliation at age 12** |  |
| Buddhism | 3 (<0.1%) |
| Christianity | 5,805 (86%) |
| Hinduism | 2 (<0.1%) |
| Islam | 11 (0.2%) |
| Judaism | 51 (0.8%) |
| No religion/Atheist/Agnostic | 697 (10%) |
| Primal, Animist, or Folk religion | 17 (0.2%) |
| Sikhism | 5 (<0.1%) |
| Some other religion | 10 (0.2%) |
| Taoism | 1 (<0.1%) |
| Missing | 122 (1.8%) |
| **Race and ethnicity** |  |
| Asian | 43 (0.6%) |
| Black | 95 (1.4%) |
| Indigenous | 129 (1.9%) |
| Mestizo(a) | 1,801 (27%) |
| Mullato(a) | 75 (1.1%) |
| Other | 104 (1.5%) |
| White | 3,406 (51%) |
| Missing | 1,070 (16%) |
| ^1^n (%) | |

***Table S1b. Regression of self-rated hope on childhood predictors for Argentina***

| Variable | Category | Estimate | SE | 95% CI | Global p-value |
| --- | --- | --- | --- | --- | --- |
| Relationship with mother (Ref: Very bad/Somewhat bad) | Very good/Somewhat good | 0.41 | 0.14 | (0.13, 0.69) | <.001 |
| Relationship with father (Ref: Very bad/Somewhat bad) | Very good/Somewhat good | 0.14 | 0.10 | (-0.06, 0.33) | <.001 |
| Parent marital status (Ref: Parents married) | No, divorced | 0.15 | 0.11 | (-0.06, 0.36) | 0.164 |
|  | Never | 0.15 | 0.10 | (-0.04, 0.33) |  |
|  | No, one or both of them had died | 0.00 | 0.21 | (-0.40, 0.41) |  |
| Subjective financial status of family growing up (Ref: Got by) | Lived comfortably | 0.15 | 0.08 | (0.00, 0.30) | 0.028 |
|  | Found it difficult | 0.12 | 0.08 | (-0.05, 0.28) |  |
|  | Found it very difficult | 0.29 | 0.15 | (-0.01, 0.58) |  |
| Abuse (Ref: No) | Yes | 0.05 | 0.08 | (-0.11, 0.20) | 0.769 |
| Outsider growing up (Ref: No) | Yes | -0.08 | 0.10 | (-0.27, 0.11) | 0.544 |
| Self-rated health growing up (Ref: Good) | Excellent | 0.26 | 0.08 | (0.09, 0.42) | <.001 |
|  | Very good | 0.00 | 0.08 | (-0.17, 0.16) |  |
|  | Fair | 0.13 | 0.16 | (-0.18, 0.44) |  |
|  | Poor | -0.04 | 0.30 | (-0.63, 0.54) |  |
| Immigration status (Ref: Born in this country) | Born in another country | 0.01 | 0.13 | (-0.25, 0.26) | 1 |
| Age 12 religious service attendance (Ref: Never) | At least 1/week | 0.03 | 0.08 | (-0.13, 0.20) | 0.002 |
|  | 1-3/month | -0.16 | 0.10 | (-0.35, 0.03) |  |
|  | <1/month | -0.16 | 0.10 | (-0.35, 0.04) |  |
| Age group (Ref: 1998-2005; current age: 18-24) | 1993-1998; age: 25-29 | 0.27 | 0.13 | (0.01, 0.53) | <.001 |
|  | 1983-1993; age: 30-39 | 0.27 | 0.11 | (0.05, 0.50) |  |
|  | 1973-1983; age 40-49 | 0.38 | 0.11 | (0.16, 0.60) |  |
|  | 1963-1973; age 50-59 | 0.45 | 0.11 | (0.23, 0.67) |  |
|  | 1953-1963; age 60-69 | 0.04 | 0.15 | (-0.25, 0.33) |  |
|  | 1943 or earlier; age 80+ | -0.32 | 0.39 | (-1.07, 0.44) |  |
|  | 1943-1953; age 70-79 | 0.06 | 0.21 | (-0.36, 0.48) |  |
| Gender (Ref: Male) | Female | 0.23 | 0.07 | (0.10, 0.36) | <.001 |
|  | Other | -0.25 | 1.12 | (-2.45, 1.94) |  |
| Religious affiliation at age 12 (Ref: No religion/Atheist/Agnostic) | Christianity | 0.57 | 0.13 | (0.30, 0.83) | <.001 |
|  | Some other religion | 0.20 | 0.26 | (-0.31, 0.70) |  |
| Race and ethnicity plurality (Ref: Majority) | Minority | 0.17 | 0.06 | (0.04, 0.29) | <.001 |

***Table S1c. Sensitivity to unmeasured confounding of childhood predictors in Argentina***

| Variable | Category | E-value for Estimate | E-value for 95% CI |
| --- | --- | --- | --- |
| Relationship with mother (Ref: Very bad/Somewhat bad)) | Very good/Somewhat good | 1.82 | 1.36 |
| Relationship with father (Ref: Very bad/Somewhat bad)) | Very good/Somewhat good | 1.37 | 1.00 |
| Parent marital status (Ref: Parents married) | No, divorced | 1.39 | 1.00 |
|  | Never | 1.38 | 1.00 |
|  | No, one or both of them had died | 1.04 | 1.00 |
| Subjective financial status of family growing up (Ref: Got by) | Lived comfortably | 1.39 | 1.00 |
|  | Found it difficult | 1.33 | 1.00 |
|  | Found it very difficult | 1.61 | 1.00 |
| Abuse (Ref: No) | Yes | 1.19 | 1.00 |
| Outsider growing up (Ref: No) | Yes | 1.25 | 1.00 |
| Self-rated health growing up (Ref: Good) | Excellent | 1.57 | 1.29 |
|  | Very good | 1.05 | 1.00 |
|  | Fair | 1.35 | 1.00 |
|  | Poor | 1.18 | 1.00 |
| Immigration status (Ref: Born in this country) | Born in another country | 1.07 | 1.00 |
| Age 12 religious service attendance (Ref: Never) | At least 1/week | 1.16 | 1.00 |
|  | 1-3/month | 1.41 | 1.00 |
|  | <1/month | 1.40 | 1.00 |
| Age group (Ref: 1998-2005; current age: 18-24) | 1993-1998; age: 25-29 | 1.60 | 1.10 |
|  | 1983-1993; age: 30-39 | 1.59 | 1.19 |
|  | 1973-1983; age 40-49 | 1.76 | 1.40 |
|  | 1963-1973; age 50-59 | 1.88 | 1.53 |
|  | 1953-1963; age 60-69 | 1.17 | 1.00 |
|  | 1943 or earlier; age 80+ | 1.67 | 1.00 |
|  | 1943-1953; age 70-79 | 1.22 | 1.00 |
| Gender (Ref: Male) | Female | 1.53 | 1.31 |
|  | Other | 1.56 | 1.00 |
| Religious affiliation at age 12 (Ref: No religion/Atheist/Agnostic) | Christianity | 2.07 | 1.64 |
|  | Some other religion | 1.47 | 1.00 |
| Race and ethnicity plurality (Ref: Majority) | Minority | 1.42 | 1.17 |

***Table S2a. Nationally representative descriptive statistics for Australia***

| **Characteristic** | **N = 3,844**^1^ |
| --- | --- |
| **Relationship with mother** |  |
| Very good | 2,554 (66%) |
| Somewhat good | 925 (24%) |
| Somewhat bad | 218 (5.7%) |
| Very bad | 107 (2.8%) |
| Does not apply | 32 (0.8%) |
| Missing | 7 (0.2%) |
| **Relationship with father** |  |
| Very good | 2,032 (53%) |
| Somewhat good | 1,144 (30%) |
| Somewhat bad | 315 (8.2%) |
| Very bad | 196 (5.1%) |
| Does not apply | 148 (3.9%) |
| Missing | 9 (0.2%) |
| **Parent marital status** |  |
| Yes, married | 3,048 (79%) |
| No, divorced | 462 (12%) |
| Never married | 187 (4.9%) |
| No, one or both of them had died | 96 (2.5%) |
| Missing | 52 (1.4%) |
| **Subjective financial status of family growing up** |  |
| Lived comfortably | 1,756 (46%) |
| Got by | 1,496 (39%) |
| Found it difficult | 422 (11%) |
| Found it very difficult | 154 (4.0%) |
| Missing | 16 (0.4%) |
| **Abuse** |  |
| Yes | 995 (26%) |
| No | 2,790 (73%) |
| Missing | 59 (1.5%) |
| **Outsider growing up** |  |
| Yes | 756 (20%) |
| No | 3,062 (80%) |
| Missing | 26 (0.7%) |
| **Self-rated health growing up** |  |
| Excellent | 1,736 (45%) |
| Very good | 1,087 (28%) |
| Good | 603 (16%) |
| Fair | 308 (8.0%) |
| Poor | 106 (2.8%) |
| Missing | 4 (<0.1%) |
| **Immigration status** |  |
| Born in this country | 2,953 (77%) |
| Born in another country | 885 (23%) |
| Missing | 6 (0.2%) |
| **Age 12 religious service attendance** |  |
| At least 1/week | 1,362 (35%) |
| 1-3/month | 486 (13%) |
| <1/month | 600 (16%) |
| Never | 1,307 (34%) |
| Missing | 90 (2.3%) |
| **Age group** |  |
| 1998-2005; current age: 18-24 | 345 (9.0%) |
| 1993-1998; age: 25-29 | 282 (7.3%) |
| 1983-1993; age: 30-39 | 641 (17%) |
| 1973-1983; age 40-49 | 618 (16%) |
| 1963-1973; age 50-59 | 691 (18%) |
| 1953-1963; age 60-69 | 589 (15%) |
| 1943 or earlier; age 80+ | 178 (4.6%) |
| 1943-1953; age 70-79 | 498 (13%) |
| Missing | 2 (<0.1%) |
| **Gender** |  |
| Male | 1,861 (48%) |
| Female | 1,941 (50%) |
| Other | 36 (0.9%) |
| Missing | 6 (0.2%) |
| **Religious affiliation at age 12** |  |
| Baha’i | 5 (0.1%) |
| Buddhism | 16 (0.4%) |
| Christianity | 2,678 (70%) |
| Hinduism | 39 (1.0%) |
| Islam | 48 (1.2%) |
| Judaism | 29 (0.8%) |
| No religion/Atheist/Agnostic | 990 (26%) |
| Primal, Animist, or Folk religion | 4 (<0.1%) |
| Sikhism | 6 (0.2%) |
| Some other religion | 8 (0.2%) |
| Taoism | 1 (<0.1%) |
| Missing | 21 (0.5%) |
| **Race and ethnicity** |  |
| Aboriginal | 53 (1.4%) |
| Australian | 1,946 (51%) |
| Australian British/European | 1,047 (27%) |
| Chinese | 75 (1.9%) |
| Indian | 58 (1.5%) |
| Japanese | 1 (<0.1%) |
| Malay | 11 (0.3%) |
| New Zealander | 91 (2.4%) |
| Other | 163 (4.2%) |
| Other European | 357 (9.3%) |
| Russian | 7 (0.2%) |
| Samoan | 4 (0.1%) |
| Sinhalese | 1 (<0.1%) |
| Spanish | 2 (<0.1%) |
| Sri Lankan Moor | 1 (<0.1%) |
| Sri Lankan Tamil | 7 (0.2%) |
| Vietnamese | 7 (0.2%) |
| Missing | 14 (0.4%) |
| ^1^n (%) | |

***Table S2b. Regression of self-rated hope on childhood predictors for Australia***

| Variable | Category | Estimate | SE | 95% CI | Global p-value |
| --- | --- | --- | --- | --- | --- |
| Relationship with mother (Ref: Very bad/Somewhat bad) | Very good/Somewhat good | -0.22 | 0.19 | (-0.59, 0.15) | 0.001 |
| Relationship with father (Ref: Very bad/Somewhat bad) | Very good/Somewhat good | 0.32 | 0.15 | (0.02, 0.62) | <.001 |
| Parent marital status (Ref: Parents married) | No, divorced | 0.06 | 0.17 | (-0.27, 0.39) | <.001 |
|  | Never | 0.61 | 0.25 | (0.12, 1.09) |  |
|  | No, one or both of them had died | 0.44 | 0.22 | (0.01, 0.86) |  |
| Subjective financial status of family growing up (Ref: Got by) | Lived comfortably | 0.05 | 0.09 | (-0.13, 0.23) | 0.999 |
|  | Found it difficult | 0.02 | 0.16 | (-0.29, 0.33) |  |
|  | Found it very difficult | -0.08 | 0.30 | (-0.68, 0.51) |  |
| Abuse (Ref: No) | Yes | -0.18 | 0.11 | (-0.39, 0.04) | 0.009 |
| Outsider growing up (Ref: No) | Yes | -0.69 | 0.14 | (-0.97, -0.42) | <.001 |
| Self-rated health growing up (Ref: Good) | Excellent | 0.58 | 0.13 | (0.33, 0.83) | <.001 |
|  | Very good | 0.31 | 0.13 | (0.05, 0.56) |  |
|  | Fair | -0.29 | 0.24 | (-0.75, 0.18) |  |
|  | Poor | -0.62 | 0.34 | (-1.29, 0.04) |  |
| Immigration status (Ref: Born in this country) | Born in another country | 0.14 | 0.11 | (-0.07, 0.35) | 0.093 |
| Age 12 religious service attendance (Ref: Never) | At least 1/week | 0.23 | 0.12 | (-0.01, 0.46) | 0.002 |
|  | 1-3/month | -0.06 | 0.16 | (-0.37, 0.24) |  |
|  | <1/month | 0.01 | 0.14 | (-0.26, 0.28) |  |
| Age group (Ref: 1998-2005; current age: 18-24) | 1993-1998; age: 25-29 | -0.23 | 0.26 | (-0.73, 0.28) | <.001 |
|  | 1983-1993; age: 30-39 | -0.10 | 0.21 | (-0.50, 0.31) |  |
|  | 1973-1983; age 40-49 | 0.10 | 0.20 | (-0.29, 0.48) |  |
|  | 1963-1973; age 50-59 | -0.09 | 0.19 | (-0.46, 0.28) |  |
|  | 1953-1963; age 60-69 | 0.22 | 0.19 | (-0.15, 0.60) |  |
|  | 1943 or earlier; age 80+ | 0.41 | 0.23 | (-0.04, 0.86) |  |
|  | 1943-1953; age 70-79 | 0.32 | 0.21 | (-0.08, 0.72) |  |
| Gender (Ref: Male) | Female | 0.19 | 0.09 | (0.02, 0.36) | <.001 |
|  | Other | -0.63 | 0.66 | (-1.92, 0.66) |  |
| Religious affiliation at age 12 (Ref: No religion/Atheist/Agnostic) | Christianity | 0.13 | 0.12 | (-0.11, 0.37) | 0.812 |
|  | Some other religion | 0.14 | 0.22 | (-0.30, 0.58) |  |
| Race and ethnicity plurality (Ref: Majority) | Minority | -0.09 | 0.09 | (-0.28, 0.09) | 0.323 |

***Table S2c. Sensitivity to unmeasured confounding of childhood predictors in Australia***

| Variable | Category | E-value for Estimate | E-value for 95% CI |
| --- | --- | --- | --- |
| Relationship with mother (Ref: Very bad/Somewhat bad)) | Very good/Somewhat good | 1.47 | 1.00 |
| Relationship with father (Ref: Very bad/Somewhat bad)) | Very good/Somewhat good | 1.61 | 1.11 |
| Parent marital status (Ref: Parents married) | No, divorced | 1.21 | 1.00 |
|  | Never | 2.01 | 1.32 |
|  | No, one or both of them had died | 1.77 | 1.10 |
| Subjective financial status of family growing up (Ref: Got by) | Lived comfortably | 1.18 | 1.00 |
|  | Found it difficult | 1.10 | 1.00 |
|  | Found it very difficult | 1.25 | 1.00 |
| Abuse (Ref: No) | Yes | 1.40 | 1.00 |
| Outsider growing up (Ref: No) | Yes | 2.14 | 1.75 |
| Self-rated health growing up (Ref: Good) | Excellent | 1.97 | 1.62 |
|  | Very good | 1.59 | 1.18 |
|  | Fair | 1.56 | 1.00 |
|  | Poor | 2.04 | 1.00 |
| Immigration status (Ref: Born in this country) | Born in another country | 1.34 | 1.00 |
| Age 12 religious service attendance (Ref: Never) | At least 1/week | 1.48 | 1.00 |
|  | 1-3/month | 1.21 | 1.00 |
|  | <1/month | 1.08 | 1.00 |
| Age group (Ref: 1998-2005; current age: 18-24) | 1993-1998; age: 25-29 | 1.47 | 1.00 |
|  | 1983-1993; age: 30-39 | 1.27 | 1.00 |
|  | 1973-1983; age 40-49 | 1.27 | 1.00 |
|  | 1963-1973; age 50-59 | 1.26 | 1.00 |
|  | 1953-1963; age 60-69 | 1.47 | 1.00 |
|  | 1943 or earlier; age 80+ | 1.73 | 1.00 |
|  | 1943-1953; age 70-79 | 1.61 | 1.00 |
| Gender (Ref: Male) | Female | 1.43 | 1.12 |
|  | Other | 2.04 | 1.00 |
| Religious affiliation at age 12 (Ref: No religion/Atheist/Agnostic) | Christianity | 1.33 | 1.00 |
|  | Some other religion | 1.34 | 1.00 |
| Race and ethnicity plurality (Ref: Majority) | Minority | 1.26 | 1.00 |

***Table S3a. Nationally representative descriptive statistics for Brazil***

| **Characteristic** | **N = 13,204**^1^ |
| --- | --- |
| **Relationship with mother** |  |
| Very good | 8,369 (63%) |
| Somewhat good | 3,559 (27%) |
| Somewhat bad | 483 (3.7%) |
| Very bad | 214 (1.6%) |
| Does not apply | 507 (3.8%) |
| Missing | 73 (0.6%) |
| **Relationship with father** |  |
| Very good | 6,364 (48%) |
| Somewhat good | 3,654 (28%) |
| Somewhat bad | 1,035 (7.8%) |
| Very bad | 756 (5.7%) |
| Does not apply | 1,303 (9.9%) |
| Missing | 93 (0.7%) |
| **Parent marital status** |  |
| Yes, married | 8,546 (65%) |
| No, divorced | 1,384 (10%) |
| Never married | 1,985 (15%) |
| No, one or both of them had died | 508 (3.8%) |
| Missing | 781 (5.9%) |
| **Subjective financial status of family growing up** |  |
| Lived comfortably | 4,998 (38%) |
| Got by | 4,616 (35%) |
| Found it difficult | 2,484 (19%) |
| Found it very difficult | 1,027 (7.8%) |
| Missing | 79 (0.6%) |
| **Abuse** |  |
| Yes | 2,606 (20%) |
| No | 10,147 (77%) |
| Missing | 451 (3.4%) |
| **Outsider growing up** |  |
| Yes | 1,659 (13%) |
| No | 11,234 (85%) |
| Missing | 311 (2.4%) |
| **Self-rated health growing up** |  |
| Excellent | 5,312 (40%) |
| Very good | 3,392 (26%) |
| Good | 2,873 (22%) |
| Fair | 1,368 (10%) |
| Poor | 228 (1.7%) |
| Missing | 30 (0.2%) |
| **Immigration status** |  |
| Born in this country | 12,688 (96%) |
| Born in another country | 153 (1.2%) |
| Missing | 363 (2.7%) |
| **Age 12 religious service attendance** |  |
| At least 1/week | 6,306 (48%) |
| 1-3/month | 2,491 (19%) |
| <1/month | 2,629 (20%) |
| Never | 1,707 (13%) |
| Missing | 71 (0.5%) |
| **Age group** |  |
| 1998-2005; current age: 18-24 | 1,986 (15%) |
| 1993-1998; age: 25-29 | 1,468 (11%) |
| 1983-1993; age: 30-39 | 2,908 (22%) |
| 1973-1983; age 40-49 | 2,638 (20%) |
| 1963-1973; age 50-59 | 2,131 (16%) |
| 1953-1963; age 60-69 | 1,435 (11%) |
| 1943 or earlier; age 80+ | 126 (1.0%) |
| 1943-1953; age 70-79 | 510 (3.9%) |
| Missing | 0 (0%) |
| **Gender** |  |
| Male | 6,320 (48%) |
| Female | 6,820 (52%) |
| Other | 35 (0.3%) |
| Missing | 30 (0.2%) |
| **Religious affiliation at age 12** |  |
| Baha’i | 1 (<0.1%) |
| Buddhism | 27 (0.2%) |
| Christianity | 11,403 (86%) |
| Confucianism | 7 (<0.1%) |
| Hinduism | 1 (<0.1%) |
| Islam | 15 (0.1%) |
| Jainism | 4 (<0.1%) |
| Judaism | 40 (0.3%) |
| No religion/Atheist/Agnostic | 908 (6.9%) |
| Primal, Animist, or Folk religion | 17 (0.1%) |
| Shinto | 4 (<0.1%) |
| Some other religion | 87 (0.7%) |
| Spiritism | 336 (2.5%) |
| Taoism | 1 (<0.1%) |
| Umbanda, Candomblé, and other African-derived religions | 262 (2.0%) |
| Missing | 94 (0.7%) |
| **Race and ethnicity** |  |
| Amarela | 238 (1.8%) |
| Branca | 5,169 (39%) |
| Indígena | 131 (1.0%) |
| Other | 61 (0.5%) |
| Parda | 5,125 (39%) |
| Preta | 1,615 (12%) |
| Missing | 865 (6.6%) |
| ^1^n (%) | |

***Table S3b. Regression of self-rated hope on childhood predictors for Brazil***

| Variable | Category | Estimate | SE | 95% CI | Global p-value |
| --- | --- | --- | --- | --- | --- |
| Relationship with mother (Ref: Very bad/Somewhat bad) | Very good/Somewhat good | 0.27 | 0.12 | (0.03, 0.50) | <.001 |
| Relationship with father (Ref: Very bad/Somewhat bad) | Very good/Somewhat good | 0.17 | 0.07 | (0.04, 0.31) | <.001 |
| Parent marital status (Ref: Parents married) | No, divorced | -0.05 | 0.07 | (-0.20, 0.09) | 0.001 |
|  | Never | 0.13 | 0.08 | (-0.02, 0.29) |  |
|  | No, one or both of them had died | -0.21 | 0.16 | (-0.53, 0.11) |  |
| Subjective financial status of family growing up (Ref: Got by) | Lived comfortably | 0.02 | 0.05 | (-0.08, 0.12) | 0.858 |
|  | Found it difficult | 0.01 | 0.07 | (-0.13, 0.15) |  |
|  | Found it very difficult | 0.15 | 0.11 | (-0.06, 0.35) |  |
| Abuse (Ref: No) | Yes | -0.08 | 0.06 | (-0.20, 0.04) | 0.102 |
| Outsider growing up (Ref: No) | Yes | -0.38 | 0.09 | (-0.55, -0.20) | <.001 |
| Self-rated health growing up (Ref: Good) | Excellent | 0.42 | 0.06 | (0.30, 0.54) | <.001 |
|  | Very good | 0.09 | 0.06 | (-0.04, 0.22) |  |
|  | Fair | -0.20 | 0.10 | (-0.41, 0.00) |  |
|  | Poor | -0.31 | 0.23 | (-0.76, 0.14) |  |
| Immigration status (Ref: Born in this country) | Born in another country | -0.12 | 0.17 | (-0.46, 0.22) | 0.705 |
| Age 12 religious service attendance (Ref: Never) | At least 1/week | 0.44 | 0.09 | (0.26, 0.62) | <.001 |
|  | 1-3/month | 0.31 | 0.10 | (0.12, 0.51) |  |
|  | <1/month | 0.26 | 0.10 | (0.07, 0.45) |  |
| Age group (Ref: 1998-2005; current age: 18-24) | 1993-1998; age: 25-29 | 0.31 | 0.09 | (0.14, 0.49) | <.001 |
|  | 1983-1993; age: 30-39 | 0.48 | 0.08 | (0.33, 0.63) |  |
|  | 1973-1983; age 40-49 | 0.50 | 0.08 | (0.34, 0.65) |  |
|  | 1963-1973; age 50-59 | 0.62 | 0.09 | (0.45, 0.79) |  |
|  | 1953-1963; age 60-69 | 0.62 | 0.10 | (0.43, 0.81) |  |
|  | 1943 or earlier; age 80+ | 0.46 | 0.26 | (-0.04, 0.96) |  |
|  | 1943-1953; age 70-79 | 0.33 | 0.18 | (-0.03, 0.69) |  |
| Gender (Ref: Male) | Female | 0.11 | 0.04 | (0.03, 0.20) | <.001 |
|  | Other | -0.26 | 0.64 | (-1.52, 1.00) |  |
| Religious affiliation at age 12 (Ref: No religion/Atheist/Agnostic) | Christianity | 0.10 | 0.10 | (-0.10, 0.31) | 0.533 |
|  | Some other religion | 0.16 | 0.14 | (-0.10, 0.43) |  |
| Race and ethnicity plurality (Ref: Majority) | Minority | -0.19 | 0.05 | (-0.29, -0.10) | <.001 |

***Table S3c. Sensitivity to unmeasured confounding of childhood predictors in Brazil***

| Variable | Category | E-value for Estimate | E-value for 95% CI |
| --- | --- | --- | --- |
| Relationship with mother (Ref: Very bad/Somewhat bad)) | Very good/Somewhat good | 1.53 | 1.14 |
| Relationship with father (Ref: Very bad/Somewhat bad)) | Very good/Somewhat good | 1.39 | 1.16 |
| Parent marital status (Ref: Parents married) | No, divorced | 1.19 | 1.00 |
|  | Never | 1.33 | 1.00 |
|  | No, one or both of them had died | 1.45 | 1.00 |
| Subjective financial status of family growing up (Ref: Got by) | Lived comfortably | 1.10 | 1.00 |
|  | Found it difficult | 1.07 | 1.00 |
|  | Found it very difficult | 1.35 | 1.00 |
| Abuse (Ref: No) | Yes | 1.24 | 1.00 |
| Outsider growing up (Ref: No) | Yes | 1.68 | 1.44 |
| Self-rated health growing up (Ref: Good) | Excellent | 1.74 | 1.58 |
|  | Very good | 1.26 | 1.00 |
|  | Fair | 1.44 | 1.01 |
|  | Poor | 1.59 | 1.00 |
| Immigration status (Ref: Born in this country) | Born in another country | 1.31 | 1.00 |
| Age 12 religious service attendance (Ref: Never) | At least 1/week | 1.77 | 1.52 |
|  | 1-3/month | 1.59 | 1.30 |
|  | <1/month | 1.52 | 1.22 |
| Age group (Ref: 1998-2005; current age: 18-24) | 1993-1998; age: 25-29 | 1.59 | 1.34 |
|  | 1983-1993; age: 30-39 | 1.83 | 1.62 |
|  | 1973-1983; age 40-49 | 1.85 | 1.64 |
|  | 1963-1973; age 50-59 | 2.03 | 1.79 |
|  | 1953-1963; age 60-69 | 2.03 | 1.76 |
|  | 1943 or earlier; age 80+ | 1.80 | 1.00 |
|  | 1943-1953; age 70-79 | 1.62 | 1.00 |
| Gender (Ref: Male) | Female | 1.30 | 1.12 |
|  | Other | 1.52 | 1.00 |
| Religious affiliation at age 12 (Ref: No religion/Atheist/Agnostic) | Christianity | 1.28 | 1.00 |
|  | Some other religion | 1.38 | 1.00 |
| Race and ethnicity plurality (Ref: Majority) | Minority | 1.43 | 1.28 |

***Table S4a. Nationally representative descriptive statistics for Egypt***

| **Characteristic** | **N = 4,729**^1^ |
| --- | --- |
| **Relationship with mother** |  |
| Very good | 4,110 (87%) |
| Somewhat good | 505 (11%) |
| Somewhat bad | 21 (0.4%) |
| Very bad | 10 (0.2%) |
| Does not apply | 83 (1.8%) |
| Missing | 0 (0%) |
| **Relationship with father** |  |
| Very good | 3,713 (79%) |
| Somewhat good | 683 (14%) |
| Somewhat bad | 56 (1.2%) |
| Very bad | 30 (0.6%) |
| Does not apply | 233 (4.9%) |
| Missing | 14 (0.3%) |
| **Parent marital status** |  |
| Yes, married | 4,049 (86%) |
| No, divorced | 131 (2.8%) |
| Never married | 9 (0.2%) |
| No, one or both of them had died | 485 (10%) |
| Missing | 55 (1.2%) |
| **Subjective financial status of family growing up** |  |
| Lived comfortably | 1,251 (26%) |
| Got by | 2,352 (50%) |
| Found it difficult | 857 (18%) |
| Found it very difficult | 268 (5.7%) |
| Missing | 1 (<0.1%) |
| **Abuse** |  |
| Yes | 405 (8.6%) |
| No | 4,293 (91%) |
| Missing | 30 (0.6%) |
| **Outsider growing up** |  |
| Yes | 260 (5.5%) |
| No | 4,456 (94%) |
| Missing | 13 (0.3%) |
| **Self-rated health growing up** |  |
| Excellent | 2,687 (57%) |
| Very good | 1,174 (25%) |
| Good | 497 (11%) |
| Fair | 265 (5.6%) |
| Poor | 106 (2.2%) |
| Missing | 1 (<0.1%) |
| **Immigration status** |  |
| Born in this country | 4,713 (100%) |
| Born in another country | 16 (0.3%) |
| Missing | 1 (<0.1%) |
| **Age 12 religious service attendance** |  |
| At least 1/week | 2,307 (49%) |
| 1-3/month | 570 (12%) |
| <1/month | 629 (13%) |
| Never | 1,165 (25%) |
| Missing | 57 (1.2%) |
| **Age group** |  |
| 1998-2005; current age: 18-24 | 960 (20%) |
| 1993-1998; age: 25-29 | 607 (13%) |
| 1983-1993; age: 30-39 | 1,204 (25%) |
| 1973-1983; age 40-49 | 897 (19%) |
| 1963-1973; age 50-59 | 613 (13%) |
| 1953-1963; age 60-69 | 387 (8.2%) |
| 1943 or earlier; age 80+ | 7 (0.2%) |
| 1943-1953; age 70-79 | 54 (1.1%) |
| Missing | 0 (0%) |
| **Gender** |  |
| Male | 2,394 (51%) |
| Female | 2,334 (49%) |
| Other | 0 (0%) |
| Missing | 0 (<0.1%) |
| **Religious affiliation at age 12** |  |
| Christianity | 123 (2.6%) |
| Islam | 4,602 (97%) |
| Jainism | 1 (<0.1%) |
| Taoism | 0 (<0.1%) |
| Missing | 3 (<0.1%) |
| **Race and ethnicity** |  |
| Arab | 4,585 (97%) |
| Bedouin Arab | 4 (<0.1%) |
| Greek | 1 (<0.1%) |
| Nubian | 27 (0.6%) |
| Turkish | 9 (0.2%) |
| Missing | 102 (2.2%) |
| ^1^n (%) | |

***Table S4b. Regression of self-rated hope on childhood predictors for Egypt***

| Variable | Category | Estimate | SE | 95% CI | Global p-value |
| --- | --- | --- | --- | --- | --- |
| Relationship with mother (Ref: Very bad/Somewhat bad) | Very good/Somewhat good | 0.15 | 0.40 | (-0.64, 0.93) | 0.715 |
| Relationship with father (Ref: Very bad/Somewhat bad) | Very good/Somewhat good | -0.20 | 0.36 | (-0.90, 0.51) | 0.389 |
| Parent marital status (Ref: Parents married) | No, divorced | -0.02 | 0.27 | (-0.56, 0.51) | 0.089 |
|  | Never | -0.24 | 0.83 | (-1.87, 1.39) |  |
|  | No, one or both of them had died | 0.30 | 0.14 | (0.02, 0.58) |  |
| Subjective financial status of family growing up (Ref: Got by) | Lived comfortably | 0.10 | 0.10 | (-0.10, 0.31) | 0.958 |
|  | Found it difficult | -0.03 | 0.13 | (-0.28, 0.22) |  |
|  | Found it very difficult | -0.06 | 0.21 | (-0.47, 0.35) |  |
| Abuse (Ref: No) | Yes | -0.13 | 0.16 | (-0.44, 0.19) | 0.56 |
| Outsider growing up (Ref: No) | Yes | 0.15 | 0.20 | (-0.25, 0.55) | 0.679 |
| Self-rated health growing up (Ref: Good) | Excellent | -0.01 | 0.16 | (-0.33, 0.30) | 0.816 |
|  | Very good | -0.16 | 0.17 | (-0.50, 0.17) |  |
|  | Fair | -0.20 | 0.20 | (-0.60, 0.20) |  |
|  | Poor | -0.16 | 0.43 | (-1.00, 0.69) |  |
| Immigration status (Ref: Born in this country) | Born in another country | 0.89 | 0.51 | (-0.11, 1.88) | 0.006 |
| Age 12 religious service attendance (Ref: Never) | At least 1/week | 0.44 | 0.12 | (0.21, 0.67) | <.001 |
|  | 1-3/month | 0.27 | 0.15 | (-0.02, 0.56) |  |
|  | <1/month | 0.22 | 0.13 | (-0.04, 0.48) |  |
| Age group (Ref: 1998-2005; current age: 18-24) | 1993-1998; age: 25-29 | -0.13 | 0.16 | (-0.45, 0.18) | 0.035 |
|  | 1983-1993; age: 30-39 | -0.10 | 0.14 | (-0.37, 0.18) |  |
|  | 1973-1983; age 40-49 | 0.00 | 0.16 | (-0.31, 0.31) |  |
|  | 1963-1973; age 50-59 | 0.29 | 0.15 | (-0.01, 0.59) |  |
|  | 1953-1963; age 60-69 | 0.18 | 0.21 | (-0.23, 0.59) |  |
|  | 1943 or earlier; age 80+ | -0.30 | 1.07 | (-2.39, 1.80) |  |
|  | 1943-1953; age 70-79 | 0.18 | 0.41 | (-0.62, 0.99) |  |
| Gender (Ref: Male) | Female | 0.64 | 0.10 | (0.45, 0.83) | <.001 |
| Religious affiliation at age 12 (Ref: Islam) | Some other religion | -0.16 | 0.32 | (-0.78, 0.46) | 0.892 |
| Race and ethnicity plurality (Ref: Majority) | Minority | 1.15 | 0.31 | (0.54, 1.76) | <.001 |

***Table S4c. Sensitivity to unmeasured confounding of childhood predictors in Egypt***

| Variable | Category | E-value for Estimate | E-value for 95% CI |
| --- | --- | --- | --- |
| Relationship with mother (Ref: Very bad/Somewhat bad)) | Very good/Somewhat good | 1.31 | 1.00 |
| Relationship with father (Ref: Very bad/Somewhat bad)) | Very good/Somewhat good | 1.37 | 1.00 |
| Parent marital status (Ref: Parents married) | No, divorced | 1.11 | 1.00 |
|  | Never | 1.42 | 1.00 |
|  | No, one or both of them had died | 1.50 | 1.11 |
| Subjective financial status of family growing up (Ref: Got by) | Lived comfortably | 1.25 | 1.00 |
|  | Found it difficult | 1.12 | 1.00 |
|  | Found it very difficult | 1.17 | 1.00 |
| Abuse (Ref: No) | Yes | 1.28 | 1.00 |
| Outsider growing up (Ref: No) | Yes | 1.31 | 1.00 |
| Self-rated health growing up (Ref: Good) | Excellent | 1.08 | 1.00 |
|  | Very good | 1.33 | 1.00 |
|  | Fair | 1.37 | 1.00 |
|  | Poor | 1.32 | 1.00 |
| Immigration status (Ref: Born in this country) | Born in another country | 2.17 | 1.00 |
| Age 12 religious service attendance (Ref: Never) | At least 1/week | 1.66 | 1.39 |
|  | 1-3/month | 1.47 | 1.00 |
|  | <1/month | 1.40 | 1.00 |
| Age group (Ref: 1998-2005; current age: 18-24) | 1993-1998; age: 25-29 | 1.29 | 1.00 |
|  | 1983-1993; age: 30-39 | 1.24 | 1.00 |
|  | 1973-1983; age 40-49 | 1.04 | 1.00 |
|  | 1963-1973; age 50-59 | 1.48 | 1.00 |
|  | 1953-1963; age 60-69 | 1.35 | 1.00 |
|  | 1943 or earlier; age 80+ | 1.49 | 1.00 |
|  | 1943-1953; age 70-79 | 1.35 | 1.00 |
| Gender (Ref: Male) | Female | 1.88 | 1.67 |
| Religious affiliation at age 12 (Ref: Islam) | Some other religion | 1.33 | 1.00 |
| Race and ethnicity plurality (Ref: Majority) | Minority | 2.50 | 1.77 |

***Table S5a. Nationally representative descriptive statistics for Germany***

| **Characteristic** | **N = 9,506**^1^ |
| --- | --- |
| **Relationship with mother** |  |
| Very good | 5,497 (58%) |
| Somewhat good | 3,031 (32%) |
| Somewhat bad | 496 (5.2%) |
| Very bad | 187 (2.0%) |
| Does not apply | 241 (2.5%) |
| Missing | 54 (0.6%) |
| **Relationship with father** |  |
| Very good | 4,652 (49%) |
| Somewhat good | 3,012 (32%) |
| Somewhat bad | 846 (8.9%) |
| Very bad | 385 (4.0%) |
| Does not apply | 538 (5.7%) |
| Missing | 73 (0.8%) |
| **Parent marital status** |  |
| Yes, married | 7,620 (80%) |
| No, divorced | 927 (9.8%) |
| Never married | 578 (6.1%) |
| No, one or both of them had died | 245 (2.6%) |
| Missing | 136 (1.4%) |
| **Subjective financial status of family growing up** |  |
| Lived comfortably | 3,177 (33%) |
| Got by | 4,508 (47%) |
| Found it difficult | 1,481 (16%) |
| Found it very difficult | 314 (3.3%) |
| Missing | 26 (0.3%) |
| **Abuse** |  |
| Yes | 1,086 (11%) |
| No | 8,321 (88%) |
| Missing | 99 (1.0%) |
| **Outsider growing up** |  |
| Yes | 1,105 (12%) |
| No | 8,262 (87%) |
| Missing | 139 (1.5%) |
| **Self-rated health growing up** |  |
| Excellent | 2,633 (28%) |
| Very good | 3,518 (37%) |
| Good | 2,582 (27%) |
| Fair | 612 (6.4%) |
| Poor | 134 (1.4%) |
| Missing | 26 (0.3%) |
| **Immigration status** |  |
| Born in this country | 8,722 (92%) |
| Born in another country | 744 (7.8%) |
| Missing | 40 (0.4%) |
| **Age 12 religious service attendance** |  |
| At least 1/week | 1,943 (20%) |
| 1-3/month | 1,899 (20%) |
| <1/month | 2,887 (30%) |
| Never | 2,749 (29%) |
| Missing | 27 (0.3%) |
| **Age group** |  |
| 1998-2005; current age: 18-24 | 829 (8.7%) |
| 1993-1998; age: 25-29 | 774 (8.1%) |
| 1983-1993; age: 30-39 | 1,438 (15%) |
| 1973-1983; age 40-49 | 1,494 (16%) |
| 1963-1973; age 50-59 | 1,729 (18%) |
| 1953-1963; age 60-69 | 1,915 (20%) |
| 1943 or earlier; age 80+ | 190 (2.0%) |
| 1943-1953; age 70-79 | 1,137 (12%) |
| Missing | 0 (0%) |
| **Gender** |  |
| Male | 4,641 (49%) |
| Female | 4,843 (51%) |
| Other | 11 (0.1%) |
| Missing | 11 (0.1%) |
| **Religious affiliation at age 12** |  |
| Baha’i | 2 (<0.1%) |
| Buddhism | 25 (0.3%) |
| Christianity | 5,751 (61%) |
| Confucianism | 4 (<0.1%) |
| Hinduism | 15 (0.2%) |
| Islam | 350 (3.7%) |
| Jainism | 1 (<0.1%) |
| Judaism | 18 (0.2%) |
| No religion/Atheist/Agnostic | 3,163 (33%) |
| Primal, Animist, or Folk religion | 19 (0.2%) |
| Sikhism | 5 (<0.1%) |
| Some other religion | 67 (0.7%) |
| Missing | 85 (0.9%) |
| **Race and ethnicity** |  |
| Missing | 9,506 (100%) |
| ^1^n (%) | |

***Table S5b. Regression of self-rated hope on childhood predictors for Germany***

| Variable | Category | Estimate | SE | 95% CI | Global p-value |
| --- | --- | --- | --- | --- | --- |
| Relationship with mother (Ref: Very bad/Somewhat bad) | Very good/Somewhat good | 0.10 | 0.11 | (-0.12, 0.32) | 0.024 |
| Relationship with father (Ref: Very bad/Somewhat bad) | Very good/Somewhat good | 0.12 | 0.08 | (-0.04, 0.28) | <.001 |
| Parent marital status (Ref: Parents married) | No, divorced | -0.03 | 0.09 | (-0.20, 0.14) | 0.908 |
|  | Never | -0.15 | 0.12 | (-0.38, 0.09) |  |
|  | No, one or both of them had died | -0.03 | 0.15 | (-0.34, 0.27) |  |
| Subjective financial status of family growing up (Ref: Got by) | Lived comfortably | 0.01 | 0.06 | (-0.11, 0.13) | 0.351 |
|  | Found it difficult | -0.08 | 0.08 | (-0.23, 0.08) |  |
|  | Found it very difficult | -0.27 | 0.17 | (-0.60, 0.05) |  |
| Abuse (Ref: No) | Yes | -0.20 | 0.09 | (-0.37, -0.03) | <.001 |
| Outsider growing up (Ref: No) | Yes | -0.44 | 0.08 | (-0.60, -0.27) | <.001 |
| Self-rated health growing up (Ref: Good) | Excellent | 0.63 | 0.08 | (0.48, 0.78) | <.001 |
|  | Very good | 0.21 | 0.07 | (0.09, 0.34) |  |
|  | Fair | -0.24 | 0.13 | (-0.49, 0.01) |  |
|  | Poor | 0.33 | 0.24 | (-0.14, 0.79) |  |
| Immigration status (Ref: Born in this country) | Born in another country | 0.20 | 0.10 | (-0.01, 0.40) | 0.002 |
| Age 12 religious service attendance (Ref: Never) | At least 1/week | 0.35 | 0.08 | (0.20, 0.50) | <.001 |
|  | 1-3/month | 0.17 | 0.08 | (0.02, 0.32) |  |
|  | <1/month | 0.17 | 0.07 | (0.04, 0.31) |  |
| Age group (Ref: 1998-2005; current age: 18-24) | 1993-1998; age: 25-29 | 0.19 | 0.13 | (-0.07, 0.44) | <.001 |
|  | 1983-1993; age: 30-39 | 0.17 | 0.11 | (-0.06, 0.39) |  |
|  | 1973-1983; age 40-49 | 0.08 | 0.12 | (-0.15, 0.31) |  |
|  | 1963-1973; age 50-59 | 0.11 | 0.12 | (-0.11, 0.34) |  |
|  | 1953-1963; age 60-69 | 0.22 | 0.12 | (-0.01, 0.45) |  |
|  | 1943 or earlier; age 80+ | 0.65 | 0.17 | (0.31, 0.98) |  |
|  | 1943-1953; age 70-79 | 0.22 | 0.13 | (-0.03, 0.47) |  |
| Gender (Ref: Male) | Female | 0.07 | 0.05 | (-0.03, 0.17) | 0.062 |
|  | Other | -0.86 | 0.70 | (-2.24, 0.52) |  |
| Religious affiliation at age 12 (Ref: No religion/Atheist/Agnostic) | Christianity | 0.06 | 0.06 | (-0.06, 0.18) | 0.34 |
|  | Some other religion | -0.11 | 0.15 | (-0.40, 0.19) |  |

***Table S5c. Sensitivity to unmeasured confounding of childhood predictors in Germany***

| Variable | Category | E-value for Estimate | E-value for 95% CI |
| --- | --- | --- | --- |
| Relationship with mother (Ref: Very bad/Somewhat bad)) | Very good/Somewhat good | 1.26 | 1.00 |
| Relationship with father (Ref: Very bad/Somewhat bad)) | Very good/Somewhat good | 1.29 | 1.00 |
| Parent marital status (Ref: Parents married) | No, divorced | 1.13 | 1.00 |
|  | Never | 1.33 | 1.00 |
|  | No, one or both of them had died | 1.14 | 1.00 |
| Subjective financial status of family growing up (Ref: Got by) | Lived comfortably | 1.07 | 1.00 |
|  | Found it difficult | 1.22 | 1.00 |
|  | Found it very difficult | 1.50 | 1.00 |
| Abuse (Ref: No) | Yes | 1.40 | 1.12 |
| Outsider growing up (Ref: No) | Yes | 1.72 | 1.51 |
| Self-rated health growing up (Ref: Good) | Excellent | 1.97 | 1.78 |
|  | Very good | 1.43 | 1.24 |
|  | Fair | 1.46 | 1.00 |
|  | Poor | 1.58 | 1.00 |
| Immigration status (Ref: Born in this country) | Born in another country | 1.40 | 1.00 |
| Age 12 religious service attendance (Ref: Never) | At least 1/week | 1.61 | 1.41 |
|  | 1-3/month | 1.36 | 1.11 |
|  | <1/month | 1.37 | 1.16 |
| Age group (Ref: 1998-2005; current age: 18-24) | 1993-1998; age: 25-29 | 1.39 | 1.00 |
|  | 1983-1993; age: 30-39 | 1.36 | 1.00 |
|  | 1973-1983; age 40-49 | 1.22 | 1.00 |
|  | 1963-1973; age 50-59 | 1.28 | 1.00 |
|  | 1953-1963; age 60-69 | 1.44 | 1.00 |
|  | 1943 or earlier; age 80+ | 1.99 | 1.56 |
|  | 1943-1953; age 70-79 | 1.44 | 1.00 |
| Gender (Ref: Male) | Female | 1.21 | 1.00 |
|  | Other | 2.28 | 1.00 |
| Religious affiliation at age 12 (Ref: No religion/Atheist/Agnostic) | Christianity | 1.19 | 1.00 |
|  | Some other religion | 1.27 | 1.00 |

***Table S6a. Nationally representative descriptive statistics for India***

| **Characteristic** | **N = 12,765**^1^ |
| --- | --- |
| **Relationship with mother** |  |
| Very good | 11,465 (90%) |
| Somewhat good | 788 (6.2%) |
| Somewhat bad | 88 (0.7%) |
| Very bad | 73 (0.6%) |
| Does not apply | 269 (2.1%) |
| Missing | 82 (0.6%) |
| **Relationship with father** |  |
| Very good | 10,923 (86%) |
| Somewhat good | 995 (7.8%) |
| Somewhat bad | 126 (1.0%) |
| Very bad | 100 (0.8%) |
| Does not apply | 481 (3.8%) |
| Missing | 141 (1.1%) |
| **Parent marital status** |  |
| Yes, married | 5,578 (44%) |
| No, divorced | 236 (1.8%) |
| Never married | 1,055 (8.3%) |
| No, one or both of them had died | 940 (7.4%) |
| Missing | 4,956 (39%) |
| **Subjective financial status of family growing up** |  |
| Lived comfortably | 4,946 (39%) |
| Got by | 3,010 (24%) |
| Found it difficult | 2,703 (21%) |
| Found it very difficult | 2,035 (16%) |
| Missing | 70 (0.5%) |
| **Abuse** |  |
| Yes | 1,468 (11%) |
| No | 10,526 (82%) |
| Missing | 771 (6.0%) |
| **Outsider growing up** |  |
| Yes | 1,926 (15%) |
| No | 10,780 (84%) |
| Missing | 59 (0.5%) |
| **Self-rated health growing up** |  |
| Excellent | 2,182 (17%) |
| Very good | 3,882 (30%) |
| Good | 4,028 (32%) |
| Fair | 2,202 (17%) |
| Poor | 424 (3.3%) |
| Missing | 47 (0.4%) |
| **Immigration status** |  |
| Born in this country | 12,629 (99%) |
| Born in another country | 110 (0.9%) |
| Missing | 26 (0.2%) |
| **Age 12 religious service attendance** |  |
| At least 1/week | 5,288 (41%) |
| 1-3/month | 2,959 (23%) |
| <1/month | 2,719 (21%) |
| Never | 1,478 (12%) |
| Missing | 321 (2.5%) |
| **Age group** |  |
| 1998-2005; current age: 18-24 | 2,543 (20%) |
| 1993-1998; age: 25-29 | 1,640 (13%) |
| 1983-1993; age: 30-39 | 3,109 (24%) |
| 1973-1983; age 40-49 | 2,275 (18%) |
| 1963-1973; age 50-59 | 1,574 (12%) |
| 1953-1963; age 60-69 | 1,188 (9.3%) |
| 1943 or earlier; age 80+ | 67 (0.5%) |
| 1943-1953; age 70-79 | 370 (2.9%) |
| Missing | 0 (0%) |
| **Gender** |  |
| Male | 6,473 (51%) |
| Female | 6,292 (49%) |
| Other | 0 (0%) |
| Missing | 0 (0%) |
| **Religious affiliation at age 12** |  |
| Buddhism | 180 (1.4%) |
| Christianity | 254 (2.0%) |
| Hinduism | 10,417 (82%) |
| Islam | 1,550 (12%) |
| Jainism | 9 (<0.1%) |
| No religion/Atheist/Agnostic | 7 (<0.1%) |
| Primal, Animist, or Folk religion | 27 (0.2%) |
| Shinto | 4 (<0.1%) |
| Sikhism | 126 (1.0%) |
| Some other religion | 59 (0.5%) |
| Missing | 131 (1.0%) |
| **Race and ethnicity** |  |
| General | 3,538 (28%) |
| Other backward caste | 4,177 (33%) |
| Schedule caste | 3,599 (28%) |
| Schedule tribe | 1,185 (9.3%) |
| Missing | 267 (2.1%) |
| ^1^n (%) | |

***Table S6b. Regression of self-rated hope on childhood predictors for India***

| Variable | Category | Estimate | SE | 95% CI | Global p-value |
| --- | --- | --- | --- | --- | --- |
| Relationship with mother (Ref: Very bad/Somewhat bad) | Very good/Somewhat good | 0.07 | 0.26 | (-0.43, 0.58) | 0.757 |
| Relationship with father (Ref: Very bad/Somewhat bad) | Very good/Somewhat good | -0.11 | 0.19 | (-0.48, 0.27) | 0.363 |
| Parent marital status (Ref: Parents married) | No, divorced | 0.26 | 0.22 | (-0.21, 0.72) | <.001 |
|  | Never | 0.00 | 0.11 | (-0.22, 0.22) |  |
|  | No, one or both of them had died | 0.35 | 0.11 | (0.13, 0.57) |  |
| Subjective financial status of family growing up (Ref: Got by) | Lived comfortably | -0.23 | 0.08 | (-0.38, -0.08) | <.001 |
|  | Found it difficult | -0.20 | 0.09 | (-0.38, -0.02) |  |
|  | Found it very difficult | -0.50 | 0.10 | (-0.70, -0.30) |  |
| Abuse (Ref: No) | Yes | -0.23 | 0.11 | (-0.44, -0.01) | <.001 |
| Outsider growing up (Ref: No) | Yes | -0.11 | 0.10 | (-0.30, 0.08) | 0.163 |
| Self-rated health growing up (Ref: Good) | Excellent | 0.13 | 0.10 | (-0.06, 0.32) | <.001 |
|  | Very good | 0.16 | 0.08 | (0.01, 0.31) |  |
|  | Fair | -0.10 | 0.09 | (-0.27, 0.06) |  |
|  | Poor | -0.37 | 0.19 | (-0.75, 0.01) |  |
| Immigration status (Ref: Born in this country) | Born in another country | -0.61 | 0.32 | (-1.24, 0.02) | 0.001 |
| Age 12 religious service attendance (Ref: Never) | At least 1/week | 0.18 | 0.11 | (-0.02, 0.39) | 0.014 |
|  | 1-3/month | 0.04 | 0.12 | (-0.19, 0.27) |  |
|  | <1/month | 0.02 | 0.11 | (-0.19, 0.24) |  |
| Age group (Ref: 1998-2005; current age: 18-24) | 1993-1998; age: 25-29 | 0.06 | 0.10 | (-0.13, 0.25) | <.001 |
|  | 1983-1993; age: 30-39 | -0.13 | 0.09 | (-0.31, 0.05) |  |
|  | 1973-1983; age 40-49 | -0.18 | 0.10 | (-0.38, 0.02) |  |
|  | 1963-1973; age 50-59 | -0.23 | 0.12 | (-0.46, 0.00) |  |
|  | 1953-1963; age 60-69 | -0.45 | 0.14 | (-0.72, -0.19) |  |
|  | 1943 or earlier; age 80+ | -0.15 | 0.49 | (-1.10, 0.80) |  |
|  | 1943-1953; age 70-79 | -0.82 | 0.22 | (-1.25, -0.38) |  |
| Gender (Ref: Male) | Female | -0.03 | 0.06 | (-0.14, 0.08) | 0.869 |
| Religious affiliation at age 12 (Ref: Hinduism) | Islam | -0.13 | 0.14 | (-0.41, 0.14) | 0.866 |
|  | Some other religion | 0.04 | 0.13 | (-0.22, 0.29) |  |
| Race and ethnicity plurality (Ref: Majority) | Minority | 0.02 | 0.07 | (-0.11, 0.16) | 0.982 |

***Table S6c. Sensitivity to unmeasured confounding of childhood predictors in India***

| Variable | Category | E-value for Estimate | E-value for 95% CI |
| --- | --- | --- | --- |
| Relationship with mother (Ref: Very bad/Somewhat bad)) | Very good/Somewhat good | 1.18 | 1.00 |
| Relationship with father (Ref: Very bad/Somewhat bad)) | Very good/Somewhat good | 1.23 | 1.00 |
| Parent marital status (Ref: Parents married) | No, divorced | 1.40 | 1.00 |
|  | Never | 1.03 | 1.00 |
|  | No, one or both of them had died | 1.48 | 1.25 |
| Subjective financial status of family growing up (Ref: Got by) | Lived comfortably | 1.37 | 1.19 |
|  | Found it difficult | 1.33 | 1.09 |
|  | Found it very difficult | 1.63 | 1.44 |
| Abuse (Ref: No) | Yes | 1.36 | 1.07 |
| Outsider growing up (Ref: No) | Yes | 1.23 | 1.00 |
| Self-rated health growing up (Ref: Good) | Excellent | 1.25 | 1.00 |
|  | Very good | 1.29 | 1.06 |
|  | Fair | 1.22 | 1.00 |
|  | Poor | 1.51 | 1.00 |
| Immigration status (Ref: Born in this country) | Born in another country | 1.74 | 1.00 |
| Age 12 religious service attendance (Ref: Never) | At least 1/week | 1.32 | 1.00 |
|  | 1-3/month | 1.12 | 1.00 |
|  | <1/month | 1.09 | 1.00 |
| Age group (Ref: 1998-2005; current age: 18-24) | 1993-1998; age: 25-29 | 1.16 | 1.00 |
|  | 1983-1993; age: 30-39 | 1.25 | 1.00 |
|  | 1973-1983; age 40-49 | 1.31 | 1.00 |
|  | 1963-1973; age 50-59 | 1.36 | 1.00 |
|  | 1953-1963; age 60-69 | 1.59 | 1.32 |
|  | 1943 or earlier; age 80+ | 1.28 | 1.00 |
|  | 1943-1953; age 70-79 | 1.93 | 1.52 |
| Gender (Ref: Male) | Female | 1.11 | 1.00 |
| Religious affiliation at age 12 (Ref: Hinduism) | Some other religion | 1.12 | 1.00 |
| Race and ethnicity plurality (Ref: Majority) | Minority | 1.09 | 1.00 |
|  | Islam | 1.26 | 1.00 |

***Table S7a. Nationally representative descriptive statistics for Indonesia***

| **Characteristic** | **N = 6,992**^1^ |
| --- | --- |
| **Relationship with mother** |  |
| Very good | 6,238 (89%) |
| Somewhat good | 583 (8.3%) |
| Somewhat bad | 50 (0.7%) |
| Very bad | 26 (0.4%) |
| Does not apply | 68 (1.0%) |
| Missing | 27 (0.4%) |
| **Relationship with father** |  |
| Very good | 6,067 (87%) |
| Somewhat good | 628 (9.0%) |
| Somewhat bad | 68 (1.0%) |
| Very bad | 52 (0.7%) |
| Does not apply | 115 (1.6%) |
| Missing | 61 (0.9%) |
| **Parent marital status** |  |
| Yes, married | 5,557 (79%) |
| No, divorced | 448 (6.4%) |
| Never married | 47 (0.7%) |
| No, one or both of them had died | 735 (11%) |
| Missing | 205 (2.9%) |
| **Subjective financial status of family growing up** |  |
| Lived comfortably | 3,408 (49%) |
| Got by | 2,955 (42%) |
| Found it difficult | 439 (6.3%) |
| Found it very difficult | 181 (2.6%) |
| Missing | 9 (0.1%) |
| **Abuse** |  |
| Yes | 486 (6.9%) |
| No | 6,427 (92%) |
| Missing | 79 (1.1%) |
| **Outsider growing up** |  |
| Yes | 343 (4.9%) |
| No | 6,639 (95%) |
| Missing | 10 (0.1%) |
| **Self-rated health growing up** |  |
| Excellent | 1,246 (18%) |
| Very good | 1,968 (28%) |
| Good | 2,490 (36%) |
| Fair | 1,233 (18%) |
| Poor | 55 (0.8%) |
| Missing | 1 (<0.1%) |
| **Immigration status** |  |
| Born in this country | 6,958 (100%) |
| Born in another country | 34 (0.5%) |
| Missing | 0 (0%) |
| **Age 12 religious service attendance** |  |
| At least 1/week | 5,363 (77%) |
| 1-3/month | 973 (14%) |
| <1/month | 329 (4.7%) |
| Never | 275 (3.9%) |
| Missing | 51 (0.7%) |
| **Age group** |  |
| 1998-2005; current age: 18-24 | 1,216 (17%) |
| 1993-1998; age: 25-29 | 849 (12%) |
| 1983-1993; age: 30-39 | 1,591 (23%) |
| 1973-1983; age 40-49 | 1,576 (23%) |
| 1963-1973; age 50-59 | 1,169 (17%) |
| 1953-1963; age 60-69 | 490 (7.0%) |
| 1943 or earlier; age 80+ | 17 (0.2%) |
| 1943-1953; age 70-79 | 83 (1.2%) |
| Missing | 0 (0%) |
| **Gender** |  |
| Male | 3,461 (50%) |
| Female | 3,513 (50%) |
| Other | 7 (<0.1%) |
| Missing | 11 (0.2%) |
| **Religious affiliation at age 12** |  |
| Buddhism | 5 (<0.1%) |
| Christianity | 528 (7.6%) |
| Confucianism | 1 (<0.1%) |
| Hinduism | 75 (1.1%) |
| Islam | 6,373 (91%) |
| Jainism | 1 (<0.1%) |
| No religion/Atheist/Agnostic | 2 (<0.1%) |
| Primal, Animist, or Folk religion | 1 (<0.1%) |
| Taoism | 0 (<0.1%) |
| Missing | 8 (0.1%) |
| **Race and ethnicity** |  |
| Bali | 69 (1.0%) |
| Banjar/Melayu Banjar | 320 (4.6%) |
| Batak | 165 (2.4%) |
| Betawi | 251 (3.6%) |
| Bugis | 243 (3.5%) |
| Jawa | 2,846 (41%) |
| Madura | 262 (3.7%) |
| Makasar | 91 (1.3%) |
| Minangkabau | 273 (3.9%) |
| Other | 1,262 (18%) |
| Sunda/Parahyangan | 1,172 (17%) |
| Missing | 38 (0.5%) |
| ^1^n (%) | |

***Table S7b. Regression of self-rated hope on childhood predictors for Indonesia***

| Variable | Category | Estimate | SE | 95% CI | Global p-value |
| --- | --- | --- | --- | --- | --- |
| Relationship with mother (Ref: Very bad/Somewhat bad) | Very good/Somewhat good | -0.17 | 0.18 | (-0.52, 0.19) | 0.02 |
| Relationship with father (Ref: Very bad/Somewhat bad) | Very good/Somewhat good | 0.04 | 0.13 | (-0.21, 0.30) | 0.81 |
| Parent marital status (Ref: Parents married) | No, divorced | -0.04 | 0.10 | (-0.24, 0.16) | 1 |
|  | Never | 0.14 | 0.27 | (-0.38, 0.66) |  |
|  | No, one or both of them had died | -0.02 | 0.09 | (-0.21, 0.16) |  |
| Subjective financial status of family growing up (Ref: Got by) | Lived comfortably | 0.07 | 0.05 | (-0.02, 0.16) | 0.466 |
|  | Found it difficult | -0.06 | 0.12 | (-0.30, 0.17) |  |
|  | Found it very difficult | -0.05 | 0.18 | (-0.41, 0.31) |  |
| Abuse (Ref: No) | Yes | -0.09 | 0.11 | (-0.30, 0.11) | 0.462 |
| Outsider growing up (Ref: No) | Yes | 0.10 | 0.11 | (-0.11, 0.32) | 0.412 |
| Self-rated health growing up (Ref: Good) | Excellent | 0.27 | 0.06 | (0.15, 0.40) | <.001 |
|  | Very good | 0.14 | 0.06 | (0.02, 0.25) |  |
|  | Fair | 0.19 | 0.07 | (0.06, 0.32) |  |
|  | Poor | 0.37 | 0.23 | (-0.09, 0.82) |  |
| Immigration status (Ref: Born in this country) | Born in another country | 0.29 | 0.20 | (-0.09, 0.67) | 0.03 |
| Age 12 religious service attendance (Ref: Never) | At least 1/week | 0.18 | 0.11 | (-0.04, 0.40) | 0.01 |
|  | 1-3/month | 0.11 | 0.11 | (-0.11, 0.34) |  |
|  | <1/month | -0.06 | 0.16 | (-0.38, 0.26) |  |
| Age group (Ref: 1998-2005; current age: 18-24) | 1993-1998; age: 25-29 | 0.02 | 0.07 | (-0.12, 0.17) | <.001 |
|  | 1983-1993; age: 30-39 | 0.06 | 0.06 | (-0.07, 0.18) |  |
|  | 1973-1983; age 40-49 | 0.08 | 0.07 | (-0.05, 0.21) |  |
|  | 1963-1973; age 50-59 | -0.17 | 0.09 | (-0.34, 0.00) |  |
|  | 1953-1963; age 60-69 | -0.39 | 0.12 | (-0.62, -0.16) |  |
|  | 1943 or earlier; age 80+ | -0.89 | 0.79 | (-2.45, 0.66) |  |
|  | 1943-1953; age 70-79 | -0.54 | 0.29 | (-1.11, 0.03) |  |
| Gender (Ref: Male) | Female | -0.02 | 0.05 | (-0.11, 0.07) | <.001 |
|  | Other | -2.40 | 0.95 | (-4.26, -0.54) |  |
| Religious affiliation at age 12 (Ref: Islam) | Christianity | 0.12 | 0.08 | (-0.05, 0.28) | 0.294 |
|  | Some other religion | 0.11 | 0.15 | (-0.19, 0.41) |  |
| Race and ethnicity plurality (Ref: Majority) | Minority | 0.04 | 0.05 | (-0.06, 0.14) | 0.597 |

***Table S7c. Sensitivity to unmeasured confounding of childhood predictors in Indonesia***

| Variable | Category | E-value for Estimate | E-value for 95% CI |
| --- | --- | --- | --- |
| Relationship with mother (Ref: Very bad/Somewhat bad)) | Very good/Somewhat good | 1.47 | 1.00 |
| Relationship with father (Ref: Very bad/Somewhat bad)) | Very good/Somewhat good | 1.19 | 1.00 |
| Parent marital status (Ref: Parents married) | No, divorced | 1.19 | 1.00 |
|  | Never | 1.41 | 1.00 |
|  | No, one or both of them had died | 1.14 | 1.00 |
| Subjective financial status of family growing up (Ref: Got by) | Lived comfortably | 1.26 | 1.00 |
|  | Found it difficult | 1.25 | 1.00 |
|  | Found it very difficult | 1.22 | 1.00 |
| Abuse (Ref: No) | Yes | 1.31 | 1.00 |
| Outsider growing up (Ref: No) | Yes | 1.33 | 1.00 |
| Self-rated health growing up (Ref: Good) | Excellent | 1.67 | 1.43 |
|  | Very good | 1.41 | 1.14 |
|  | Fair | 1.50 | 1.23 |
|  | Poor | 1.84 | 1.00 |
| Immigration status (Ref: Born in this country) | Born in another country | 1.70 | 1.00 |
| Age 12 religious service attendance (Ref: Never) | At least 1/week | 1.48 | 1.00 |
|  | 1-3/month | 1.36 | 1.00 |
|  | <1/month | 1.23 | 1.00 |
| Age group (Ref: 1998-2005; current age: 18-24) | 1993-1998; age: 25-29 | 1.13 | 1.00 |
|  | 1983-1993; age: 30-39 | 1.23 | 1.00 |
|  | 1973-1983; age 40-49 | 1.30 | 1.00 |
|  | 1963-1973; age 50-59 | 1.48 | 1.05 |
|  | 1953-1963; age 60-69 | 1.88 | 1.45 |
|  | 1943 or earlier; age 80+ | 2.93 | 1.00 |
|  | 1943-1953; age 70-79 | 2.17 | 1.00 |
| Gender (Ref: Male) | Female | 1.14 | 1.00 |
|  | Other | 8.69 | 2.18 |
| Religious affiliation at age 12 (Ref: Islam) | Christianity | 1.37 | 1.00 |
|  | Some other religion | 1.36 | 1.00 |
| Race and ethnicity plurality (Ref: Majority) | Minority | 1.19 | 1.00 |

***Table S8a. Nationally representative descriptive statistics for Israel***

| **Characteristic** | **N = 3,669**^1^ |
| --- | --- |
| **Relationship with mother** |  |
| Very good | 2,686 (73%) |
| Somewhat good | 793 (22%) |
| Somewhat bad | 110 (3.0%) |
| Very bad | 18 (0.5%) |
| Does not apply | 45 (1.2%) |
| Missing | 17 (0.5%) |
| **Relationship with father** |  |
| Very good | 2,290 (62%) |
| Somewhat good | 912 (25%) |
| Somewhat bad | 234 (6.4%) |
| Very bad | 37 (1.0%) |
| Does not apply | 171 (4.7%) |
| Missing | 25 (0.7%) |
| **Parent marital status** |  |
| Yes, married | 3,172 (86%) |
| No, divorced | 284 (7.8%) |
| Never married | 36 (1.0%) |
| No, one or both of them had died | 130 (3.5%) |
| Missing | 47 (1.3%) |
| **Subjective financial status of family growing up** |  |
| Lived comfortably | 923 (25%) |
| Got by | 1,822 (50%) |
| Found it difficult | 667 (18%) |
| Found it very difficult | 239 (6.5%) |
| Missing | 17 (0.5%) |
| **Abuse** |  |
| Yes | 0 (0%) |
| No | 0 (0%) |
| Missing | 3,669 (100%) |
| **Outsider growing up** |  |
| Yes | 371 (10%) |
| No | 3,228 (88%) |
| Missing | 70 (1.9%) |
| **Self-rated health growing up** |  |
| Excellent | 1,785 (49%) |
| Very good | 1,284 (35%) |
| Good | 480 (13%) |
| Fair | 105 (2.9%) |
| Poor | 6 (0.2%) |
| Missing | 8 (0.2%) |
| **Immigration status** |  |
| Born in this country | 2,796 (76%) |
| Born in another country | 868 (24%) |
| Missing | 5 (0.1%) |
| **Age 12 religious service attendance** |  |
| At least 1/week | 867 (24%) |
| 1-3/month | 435 (12%) |
| <1/month | 810 (22%) |
| Never | 1,539 (42%) |
| Missing | 17 (0.5%) |
| **Age group** |  |
| 1998-2005; current age: 18-24 | 553 (15%) |
| 1993-1998; age: 25-29 | 407 (11%) |
| 1983-1993; age: 30-39 | 666 (18%) |
| 1973-1983; age 40-49 | 616 (17%) |
| 1963-1973; age 50-59 | 542 (15%) |
| 1953-1963; age 60-69 | 469 (13%) |
| 1943 or earlier; age 80+ | 79 (2.2%) |
| 1943-1953; age 70-79 | 336 (9.2%) |
| Missing | 0 (0%) |
| **Gender** |  |
| Male | 1,791 (49%) |
| Female | 1,872 (51%) |
| Other | 0 (<0.1%) |
| Missing | 6 (0.2%) |
| **Religious affiliation at age 12** |  |
| Baha’i | 1 (<0.1%) |
| Christianity | 60 (1.6%) |
| Islam | 647 (18%) |
| Judaism | 2,873 (78%) |
| No religion/Atheist/Agnostic | 69 (1.9%) |
| Primal, Animist, or Folk religion | 3 (<0.1%) |
| Sikhism | 1 (<0.1%) |
| Some other religion | 5 (0.1%) |
| Missing | 10 (0.3%) |
| **Race and ethnicity** |  |
| Arab | 674 (18%) |
| Jewish | 2,926 (80%) |
| Other | 39 (1.1%) |
| Missing | 30 (0.8%) |
| ^1^n (%) | |

***Table S8b. Regression of self-rated hope on childhood predictors for Israel***

| Variable | Category | Estimate | SE | 95% CI | Global p-value |
| --- | --- | --- | --- | --- | --- |
| Relationship with mother (Ref: Very bad/Somewhat bad) | Very good/Somewhat good | -0.38 | 0.18 | (-0.72, -0.03) | <.001 |
| Relationship with father (Ref: Very bad/Somewhat bad) | Very good/Somewhat good | 0.13 | 0.13 | (-0.13, 0.39) | 0.015 |
| Parent marital status (Ref: Parents married) | No, divorced | -0.46 | 0.16 | (-0.78, -0.14) | <.001 |
|  | Never | -1.00 | 0.33 | (-1.65, -0.35) |  |
|  | No, one or both of them had died | -0.27 | 0.17 | (-0.62, 0.07) |  |
| Subjective financial status of family growing up (Ref: Got by) | Lived comfortably | 0.00 | 0.07 | (-0.14, 0.13) | 0.974 |
|  | Found it difficult | 0.06 | 0.09 | (-0.11, 0.23) |  |
|  | Found it very difficult | -0.16 | 0.21 | (-0.56, 0.24) |  |
| Outsider growing up (Ref: No) | Yes | -0.30 | 0.14 | (-0.58, -0.01) | <.001 |
| Self-rated health growing up (Ref: Good) | Excellent | 0.44 | 0.15 | (0.13, 0.74) | <.001 |
|  | Very good | 0.44 | 0.14 | (0.16, 0.71) |  |
|  | Fair | -0.23 | 0.20 | (-0.62, 0.17) |  |
|  | Poor | 0.61 | 1.36 | (-2.07, 3.28) |  |
| Immigration status (Ref: Born in this country) | Born in another country | -0.16 | 0.11 | (-0.37, 0.06) | 0.028 |
| Age 12 religious service attendance (Ref: Never) | At least 1/week | 0.31 | 0.12 | (0.07, 0.56) | <.001 |
|  | 1-3/month | 0.36 | 0.13 | (0.11, 0.60) |  |
|  | <1/month | 0.34 | 0.10 | (0.14, 0.54) |  |
| Age group (Ref: 1998-2005; current age: 18-24) | 1993-1998; age: 25-29 | -0.03 | 0.13 | (-0.28, 0.22) | <.001 |
|  | 1983-1993; age: 30-39 | -0.16 | 0.12 | (-0.40, 0.08) |  |
|  | 1973-1983; age 40-49 | -0.12 | 0.13 | (-0.38, 0.14) |  |
|  | 1963-1973; age 50-59 | -0.33 | 0.13 | (-0.58, -0.08) |  |
|  | 1953-1963; age 60-69 | -0.13 | 0.13 | (-0.38, 0.12) |  |
|  | 1943 or earlier; age 80+ | -1.18 | 0.36 | (-1.90, -0.47) |  |
|  | 1943-1953; age 70-79 | -0.81 | 0.18 | (-1.18, -0.45) |  |
| Gender (Ref: Male) | Female | -0.06 | 0.08 | (-0.22, 0.09) | 0.028 |
|  | Other | -0.44 | 0.23 | (-0.89, 0.00) |  |
| Religious affiliation at age 12 (Ref: Judaism) | Islam | -1.02 | 0.47 | (-1.93, -0.10) | 0.007 |
|  | Some other religion | -0.18 | 0.28 | (-0.72, 0.37) |  |
| Race and ethnicity plurality (Ref: Majority) | Minority | -0.02 | 0.41 | (-0.84, 0.79) | 1 |

***Table S8c. Sensitivity to unmeasured confounding of childhood predictors in Israel***

| Variable | Category | E-value for Estimate | E-value for 95% CI |
| --- | --- | --- | --- |
| Relationship with mother (Ref: Very bad/Somewhat bad)) | Very good/Somewhat good | 1.76 | 1.16 |
| Relationship with father (Ref: Very bad/Somewhat bad)) | Very good/Somewhat good | 1.35 | 1.00 |
| Parent marital status (Ref: Parents married) | No, divorced | 1.89 | 1.38 |
|  | Never | 2.84 | 1.71 |
|  | No, one or both of them had died | 1.59 | 1.00 |
| Subjective financial status of family growing up (Ref: Got by) | Lived comfortably | 1.05 | 1.00 |
|  | Found it difficult | 1.22 | 1.00 |
|  | Found it very difficult | 1.40 | 1.00 |
| Outsider growing up (Ref: No) | Yes | 1.63 | 1.09 |
| Self-rated health growing up (Ref: Good) | Excellent | 1.85 | 1.36 |
|  | Very good | 1.85 | 1.41 |
|  | Fair | 1.52 | 1.00 |
|  | Poor | 2.13 | 1.00 |
| Immigration status (Ref: Born in this country) | Born in another country | 1.40 | 1.00 |
| Age 12 religious service attendance (Ref: Never) | At least 1/week | 1.66 | 1.24 |
|  | 1-3/month | 1.72 | 1.31 |
|  | <1/month | 1.70 | 1.38 |
| Age group (Ref: 1998-2005; current age: 18-24) | 1993-1998; age: 25-29 | 1.13 | 1.00 |
|  | 1983-1993; age: 30-39 | 1.41 | 1.00 |
|  | 1973-1983; age 40-49 | 1.34 | 1.00 |
|  | 1963-1973; age 50-59 | 1.68 | 1.26 |
|  | 1953-1963; age 60-69 | 1.36 | 1.00 |
|  | 1943 or earlier; age 80+ | 3.21 | 1.91 |
|  | 1943-1953; age 70-79 | 2.49 | 1.88 |
| Gender (Ref: Male) | Female | 1.22 | 1.00 |
|  | Other | 1.86 | 1.03 |
| Religious affiliation at age 12 (Ref: Judaism) | Some other religion | 1.43 | 1.00 |
| Race and ethnicity plurality (Ref: Majority) | Minority | 1.13 | 1.00 |
|  | Islam | 2.87 | 1.30 |

***Table S9a. Nationally representative descriptive statistics for Japan***

| **Characteristic** | **N = 20,543**^1^ |
| --- | --- |
| **Relationship with mother** |  |
| Very good | 5,630 (27%) |
| Somewhat good | 9,461 (46%) |
| Somewhat bad | 2,750 (13%) |
| Very bad | 799 (3.9%) |
| Does not apply | 1,838 (8.9%) |
| Missing | 66 (0.3%) |
| **Relationship with father** |  |
| Very good | 4,156 (20%) |
| Somewhat good | 9,081 (44%) |
| Somewhat bad | 3,446 (17%) |
| Very bad | 1,223 (6.0%) |
| Does not apply | 2,580 (13%) |
| Missing | 57 (0.3%) |
| **Parent marital status** |  |
| Yes, married | 17,713 (86%) |
| No, divorced | 1,127 (5.5%) |
| Never married | 591 (2.9%) |
| No, one or both of them had died | 754 (3.7%) |
| Missing | 359 (1.7%) |
| **Subjective financial status of family growing up** |  |
| Lived comfortably | 8,320 (41%) |
| Got by | 8,799 (43%) |
| Found it difficult | 2,398 (12%) |
| Found it very difficult | 973 (4.7%) |
| Missing | 52 (0.3%) |
| **Abuse** |  |
| Yes | 1,482 (7.2%) |
| No | 18,964 (92%) |
| Missing | 96 (0.5%) |
| **Outsider growing up** |  |
| Yes | 1,963 (9.6%) |
| No | 17,136 (83%) |
| Missing | 1,444 (7.0%) |
| **Self-rated health growing up** |  |
| Excellent | 2,711 (13%) |
| Very good | 7,106 (35%) |
| Good | 6,689 (33%) |
| Fair | 3,199 (16%) |
| Poor | 758 (3.7%) |
| Missing | 80 (0.4%) |
| **Immigration status** |  |
| Born in this country | 19,548 (95%) |
| Born in another country | 158 (0.8%) |
| Missing | 837 (4.1%) |
| **Age 12 religious service attendance** |  |
| At least 1/week | 398 (1.9%) |
| 1-3/month | 883 (4.3%) |
| <1/month | 5,023 (24%) |
| Never | 14,117 (69%) |
| Missing | 123 (0.6%) |
| **Age group** |  |
| 1998-2005; current age: 18-24 | 1,589 (7.7%) |
| 1993-1998; age: 25-29 | 806 (3.9%) |
| 1983-1993; age: 30-39 | 2,851 (14%) |
| 1973-1983; age 40-49 | 3,363 (16%) |
| 1963-1973; age 50-59 | 3,770 (18%) |
| 1953-1963; age 60-69 | 4,118 (20%) |
| 1943 or earlier; age 80+ | 493 (2.4%) |
| 1943-1953; age 70-79 | 3,554 (17%) |
| Missing | 0 (0%) |
| **Gender** |  |
| Male | 9,847 (48%) |
| Female | 10,602 (52%) |
| Other | 28 (0.1%) |
| Missing | 66 (0.3%) |
| **Religious affiliation at age 12** |  |
| Baha’i | 7 (<0.1%) |
| Buddhism | 6,536 (32%) |
| Christianity | 343 (1.7%) |
| Confucianism | 25 (0.1%) |
| Hinduism | 4 (<0.1%) |
| Islam | 7 (<0.1%) |
| Jainism | 1 (<0.1%) |
| No religion/Atheist/Agnostic | 12,950 (63%) |
| Primal, Animist, or Folk religion | 13 (<0.1%) |
| Shinto | 382 (1.9%) |
| Some other religion | 46 (0.2%) |
| Taoism | 14 (<0.1%) |
| Missing | 215 (1.0%) |
| **Race and ethnicity** |  |
| Missing | 20,543 (100%) |
| ^1^n (%) | |

***Table S9b. Regression of self-rated hope on childhood predictors for Japan***

| Variable | Category | Estimate | SE | 95% CI | Global p-value |
| --- | --- | --- | --- | --- | --- |
| Relationship with mother (Ref: Very bad/Somewhat bad) | Very good/Somewhat good | 0.18 | 0.05 | (0.08, 0.28) | <.001 |
| Relationship with father (Ref: Very bad/Somewhat bad) | Very good/Somewhat good | 0.28 | 0.05 | (0.19, 0.37) | <.001 |
| Parent marital status (Ref: Parents married) | No, divorced | 0.17 | 0.08 | (0.01, 0.33) | 0.001 |
|  | Never | 0.11 | 0.10 | (-0.08, 0.31) |  |
|  | No, one or both of them had died | 0.18 | 0.10 | (-0.01, 0.37) |  |
| Subjective financial status of family growing up (Ref: Got by) | Lived comfortably | 0.30 | 0.04 | (0.23, 0.37) | <.001 |
|  | Found it difficult | -0.10 | 0.06 | (-0.21, 0.01) |  |
|  | Found it very difficult | -0.36 | 0.11 | (-0.57, -0.16) |  |
| Abuse (Ref: No) | Yes | -0.05 | 0.08 | (-0.20, 0.11) | 0.839 |
| Outsider growing up (Ref: No) | Yes | -0.15 | 0.07 | (-0.30, -0.01) | <.001 |
| Self-rated health growing up (Ref: Good) | Excellent | 1.32 | 0.06 | (1.20, 1.43) | <.001 |
|  | Very good | 0.56 | 0.04 | (0.48, 0.64) |  |
|  | Fair | -0.46 | 0.05 | (-0.57, -0.36) |  |
|  | Poor | -0.62 | 0.12 | (-0.86, -0.39) |  |
| Immigration status (Ref: Born in this country) | Born in another country | 0.34 | 0.18 | (-0.02, 0.70) | 0.002 |
| Age 12 religious service attendance (Ref: Never) | At least 1/week | 0.61 | 0.14 | (0.34, 0.88) | <.001 |
|  | 1-3/month | 0.84 | 0.08 | (0.68, 0.99) |  |
|  | <1/month | 0.25 | 0.04 | (0.18, 0.33) |  |
| Age group (Ref: 1998-2005; current age: 18-24) | 1993-1998; age: 25-29 | -0.24 | 0.11 | (-0.45, -0.04) |  |
|  | 1983-1993; age: 30-39 | -0.17 | 0.08 | (-0.33, -0.01) |  |
|  | 1973-1983; age 40-49 | -0.16 | 0.08 | (-0.32, -0.01) |  |
|  | 1963-1973; age 50-59 | 0.00 | 0.08 | (-0.15, 0.15) |  |
|  | 1953-1963; age 60-69 | 0.40 | 0.08 | (0.25, 0.55) |  |
|  | 1943 or earlier; age 80+ | 1.24 | 0.12 | (1.00, 1.48) |  |
|  | 1943-1953; age 70-79 | 0.97 | 0.08 | (0.82, 1.12) |  |
| Gender (Ref: Male) | Female | 0.09 | 0.03 | (0.03, 0.16) | <.001 |
|  | Other | 0.18 | 0.41 | (-0.63, 0.99) |  |
| Religious affiliation at age 12 (Ref: No religion/Atheist/Agnostic) | Buddhism | 0.17 | 0.04 | (0.10, 0.25) | <.001 |
|  | Some other religion | 0.25 | 0.09 | (0.06, 0.43) |  |

***Table S9c. Sensitivity to unmeasured confounding of childhood predictors in Japan***

| Variable | Category | E-value for Estimate | E-value for 95% CI |
| --- | --- | --- | --- |
| Relationship with mother (Ref: Very bad/Somewhat bad)) | Very good/Somewhat good | 1.36 | 1.22 |
| Relationship with father (Ref: Very bad/Somewhat bad)) | Very good/Somewhat good | 1.48 | 1.37 |
| Parent marital status (Ref: Parents married) | No, divorced | 1.34 | 1.06 |
|  | Never | 1.27 | 1.00 |
|  | No, one or both of them had died | 1.35 | 1.00 |
| Subjective financial status of family growing up (Ref: Got by) | Lived comfortably | 1.50 | 1.41 |
|  | Found it difficult | 1.24 | 1.00 |
|  | Found it very difficult | 1.58 | 1.33 |
| Abuse (Ref: No) | Yes | 1.15 | 1.00 |
| Outsider growing up (Ref: No) | Yes | 1.32 | 1.07 |
| Self-rated health growing up (Ref: Good) | Excellent | 2.75 | 2.59 |
|  | Very good | 1.80 | 1.71 |
|  | Fair | 1.69 | 1.57 |
|  | Poor | 1.88 | 1.61 |
| Immigration status (Ref: Born in this country) | Born in another country | 1.55 | 1.00 |
| Age 12 religious service attendance (Ref: Never) | At least 1/week | 1.86 | 1.55 |
|  | 1-3/month | 2.13 | 1.94 |
|  | <1/month | 1.45 | 1.35 |
| Age group (Ref: 1998-2005; current age: 18-24) | 1993-1998; age: 25-29 | 1.43 | 1.14 |
|  | 1983-1993; age: 30-39 | 1.35 | 1.08 |
|  | 1973-1983; age 40-49 | 1.33 | 1.06 |
|  | 1963-1973; age 50-59 | 1.04 | 1.00 |
|  | 1953-1963; age 60-69 | 1.61 | 1.44 |
|  | 1943 or earlier; age 80+ | 2.65 | 2.34 |
|  | 1943-1953; age 70-79 | 2.29 | 2.11 |
| Gender (Ref: Male) | Female | 1.23 | 1.11 |
|  | Other | 1.36 | 1.00 |
| Religious affiliation at age 12 (Ref: No religion/Atheist/Agnostic) | Some other religion | 1.44 | 1.19 |
|  | Buddhism | 1.34 | 1.24 |

***Table S10a. Nationally representative descriptive statistics for Kenya***

| **Characteristic** | **N = 11,389**^1^ |
| --- | --- |
| **Relationship with mother** |  |
| Very good | 9,418 (83%) |
| Somewhat good | 1,435 (13%) |
| Somewhat bad | 130 (1.1%) |
| Very bad | 100 (0.9%) |
| Does not apply | 240 (2.1%) |
| Missing | 66 (0.6%) |
| **Relationship with father** |  |
| Very good | 7,958 (70%) |
| Somewhat good | 1,896 (17%) |
| Somewhat bad | 216 (1.9%) |
| Very bad | 220 (1.9%) |
| Does not apply | 967 (8.5%) |
| Missing | 132 (1.2%) |
| **Parent marital status** |  |
| Yes, married | 9,238 (81%) |
| No, divorced | 697 (6.1%) |
| Never married | 681 (6.0%) |
| No, one or both of them had died | 471 (4.1%) |
| Missing | 301 (2.6%) |
| **Subjective financial status of family growing up** |  |
| Lived comfortably | 3,026 (27%) |
| Got by | 3,279 (29%) |
| Found it difficult | 4,071 (36%) |
| Found it very difficult | 994 (8.7%) |
| Missing | 19 (0.2%) |
| **Abuse** |  |
| Yes | 1,300 (11%) |
| No | 10,039 (88%) |
| Missing | 49 (0.4%) |
| **Outsider growing up** |  |
| Yes | 1,223 (11%) |
| No | 10,114 (89%) |
| Missing | 52 (0.5%) |
| **Self-rated health growing up** |  |
| Excellent | 4,449 (39%) |
| Very good | 2,598 (23%) |
| Good | 2,582 (23%) |
| Fair | 1,384 (12%) |
| Poor | 349 (3.1%) |
| Missing | 26 (0.2%) |
| **Immigration status** |  |
| Born in this country | 11,270 (99%) |
| Born in another country | 117 (1.0%) |
| Missing | 2 (<0.1%) |
| **Age 12 religious service attendance** |  |
| At least 1/week | 9,189 (81%) |
| 1-3/month | 1,687 (15%) |
| <1/month | 236 (2.1%) |
| Never | 198 (1.7%) |
| Missing | 79 (0.7%) |
| **Age group** |  |
| 1998-2005; current age: 18-24 | 2,868 (25%) |
| 1993-1998; age: 25-29 | 2,035 (18%) |
| 1983-1993; age: 30-39 | 2,564 (23%) |
| 1973-1983; age 40-49 | 1,708 (15%) |
| 1963-1973; age 50-59 | 1,072 (9.4%) |
| 1953-1963; age 60-69 | 710 (6.2%) |
| 1943 or earlier; age 80+ | 67 (0.6%) |
| 1943-1953; age 70-79 | 360 (3.2%) |
| Missing | 5 (<0.1%) |
| **Gender** |  |
| Male | 5,567 (49%) |
| Female | 5,813 (51%) |
| Other | 2 (<0.1%) |
| Missing | 7 (<0.1%) |
| **Religious affiliation at age 12** |  |
| Baha’i | 3 (<0.1%) |
| Buddhism | 5 (<0.1%) |
| Christianity | 10,369 (91%) |
| Islam | 916 (8.0%) |
| Jainism | 1 (<0.1%) |
| Judaism | 6 (<0.1%) |
| No religion/Atheist/Agnostic | 67 (0.6%) |
| Primal, Animist, or Folk religion | 13 (0.1%) |
| Sikhism | 0 (<0.1%) |
| Some other religion | 0 (<0.1%) |
| Missing | 9 (<0.1%) |
| **Race and ethnicity** |  |
| Embu | 197 (1.7%) |
| Kalenjin | 1,377 (12%) |
| Kamba | 1,299 (11%) |
| Kenyan Somali/Somali | 396 (3.5%) |
| Kikuyu | 2,119 (19%) |
| Kisii | 789 (6.9%) |
| Luhya | 1,943 (17%) |
| Luo | 1,120 (9.8%) |
| Maasai | 237 (2.1%) |
| Meru | 630 (5.5%) |
| Miji Kenda tribes | 708 (6.2%) |
| Other | 548 (4.8%) |
| Missing | 27 (0.2%) |
| ^1^n (%) | |

***Table S10b. Regression of self-rated hope on childhood predictors for Kenya***

| Variable | Category | Estimate | SE | 95% CI | Global p-value |
| --- | --- | --- | --- | --- | --- |
| Relationship with mother (Ref: Very bad/Somewhat bad) | Very good/Somewhat good | 0.06 | 0.20 | (-0.33, 0.45) | 0.709 |
| Relationship with father (Ref: Very bad/Somewhat bad) | Very good/Somewhat good | 0.05 | 0.12 | (-0.18, 0.28) | 0.569 |
| Parent marital status (Ref: Parents married) | No, divorced | -0.22 | 0.14 | (-0.50, 0.06) | <.001 |
|  | Never | -0.28 | 0.14 | (-0.56, 0.01) |  |
|  | No, one or both of them had died | 0.36 | 0.13 | (0.10, 0.63) |  |
| Subjective financial status of family growing up (Ref: Got by) | Lived comfortably | -0.07 | 0.07 | (-0.21, 0.06) | 0.34 |
|  | Found it difficult | -0.11 | 0.06 | (-0.23, 0.01) |  |
|  | Found it very difficult | -0.14 | 0.14 | (-0.42, 0.13) |  |
| Abuse (Ref: No) | Yes | -0.52 | 0.10 | (-0.71, -0.33) | <.001 |
| Outsider growing up (Ref: No) | Yes | -0.16 | 0.11 | (-0.37, 0.05) | 0.028 |
| Self-rated health growing up (Ref: Good) | Excellent | 0.10 | 0.08 | (-0.06, 0.26) | 0.988 |
|  | Very good | 0.04 | 0.08 | (-0.11, 0.20) |  |
|  | Fair | 0.10 | 0.10 | (-0.09, 0.30) |  |
|  | Poor | 0.12 | 0.21 | (-0.30, 0.54) |  |
| Immigration status (Ref: Born in this country) | Born in another country | -0.57 | 0.30 | (-1.15, 0.02) | 0.002 |
| Age 12 religious service attendance (Ref: Never) | At least 1/week | 0.28 | 0.30 | (-0.31, 0.86) | 0.992 |
|  | 1-3/month | 0.25 | 0.30 | (-0.34, 0.84) |  |
|  | <1/month | 0.14 | 0.35 | (-0.55, 0.84) |  |
| Age group (Ref: 1998-2005; current age: 18-24) | 1993-1998; age: 25-29 | 0.00 | 0.07 | (-0.13, 0.14) | <.001 |
|  | 1983-1993; age: 30-39 | -0.06 | 0.07 | (-0.19, 0.07) |  |
|  | 1973-1983; age 40-49 | -0.23 | 0.09 | (-0.42, -0.05) |  |
|  | 1963-1973; age 50-59 | -0.35 | 0.12 | (-0.58, -0.11) |  |
|  | 1953-1963; age 60-69 | -0.37 | 0.16 | (-0.68, -0.07) |  |
|  | 1943 or earlier; age 80+ | -0.02 | 0.41 | (-0.82, 0.79) |  |
|  | 1943-1953; age 70-79 | -0.13 | 0.17 | (-0.47, 0.21) |  |
| Gender (Ref: Male) | Female | -0.03 | 0.05 | (-0.13, 0.08) | <.001 |
|  | Other | 1.02 | 0.17 | (0.69, 1.36) |  |
| Religious affiliation at age 12 (Ref: Christianity) | Islam | -0.35 | 0.13 | (-0.60, -0.10) | <.001 |
|  | Some other religion | -0.21 | 0.46 | (-1.12, 0.69) |  |
| Race and ethnicity plurality (Ref: Majority) | Minority | -0.22 | 0.07 | (-0.37, -0.08) | <.001 |

***Table S10c. Sensitivity to unmeasured confounding of childhood predictors in Kenya***

| Variable | Category | E-value for Estimate | E-value for 95% CI |
| --- | --- | --- | --- |
| Relationship with mother (Ref: Very bad/Somewhat bad)) | Very good/Somewhat good | 1.18 | 1.00 |
| Relationship with father (Ref: Very bad/Somewhat bad)) | Very good/Somewhat good | 1.16 | 1.00 |
| Parent marital status (Ref: Parents married) | No, divorced | 1.40 | 1.00 |
|  | Never | 1.47 | 1.00 |
|  | No, one or both of them had died | 1.57 | 1.24 |
| Subjective financial status of family growing up (Ref: Got by) | Lived comfortably | 1.20 | 1.00 |
|  | Found it difficult | 1.26 | 1.00 |
|  | Found it very difficult | 1.30 | 1.00 |
| Abuse (Ref: No) | Yes | 1.75 | 1.53 |
| Outsider growing up (Ref: No) | Yes | 1.32 | 1.00 |
| Self-rated health growing up (Ref: Good) | Excellent | 1.24 | 1.00 |
|  | Very good | 1.14 | 1.00 |
|  | Fair | 1.25 | 1.00 |
|  | Poor | 1.27 | 1.00 |
| Immigration status (Ref: Born in this country) | Born in another country | 1.80 | 1.00 |
| Age 12 religious service attendance (Ref: Never) | At least 1/week | 1.47 | 1.00 |
|  | 1-3/month | 1.44 | 1.00 |
|  | <1/month | 1.30 | 1.00 |
| Age group (Ref: 1998-2005; current age: 18-24) | 1993-1998; age: 25-29 | 1.04 | 1.00 |
|  | 1983-1993; age: 30-39 | 1.18 | 1.00 |
|  | 1973-1983; age 40-49 | 1.42 | 1.16 |
|  | 1963-1973; age 50-59 | 1.55 | 1.26 |
|  | 1953-1963; age 60-69 | 1.58 | 1.19 |
|  | 1943 or earlier; age 80+ | 1.09 | 1.00 |
|  | 1943-1953; age 70-79 | 1.28 | 1.00 |
| Gender (Ref: Male) | Female | 1.12 | 1.00 |
|  | Other | 2.34 | 1.94 |
| Religious affiliation at age 12 (Ref: Christianity) | Some other religion | 1.39 | 1.00 |
| Race and ethnicity plurality (Ref: Majority) | Minority | 1.40 | 1.21 |
|  | Islam | 1.55 | 1.24 |

***Table S11a. Nationally representative descriptive statistics for Mexico***

| **Characteristic** | **N = 5,776**^1^ |
| --- | --- |
| **Relationship with mother** |  |
| Very good | 3,912 (68%) |
| Somewhat good | 1,340 (23%) |
| Somewhat bad | 177 (3.1%) |
| Very bad | 90 (1.6%) |
| Does not apply | 177 (3.1%) |
| Missing | 80 (1.4%) |
| **Relationship with father** |  |
| Very good | 3,089 (53%) |
| Somewhat good | 1,556 (27%) |
| Somewhat bad | 335 (5.8%) |
| Very bad | 267 (4.6%) |
| Does not apply | 470 (8.1%) |
| Missing | 60 (1.0%) |
| **Parent marital status** |  |
| Yes, married | 3,999 (69%) |
| No, divorced | 341 (5.9%) |
| Never married | 827 (14%) |
| No, one or both of them had died | 176 (3.0%) |
| Missing | 432 (7.5%) |
| **Subjective financial status of family growing up** |  |
| Lived comfortably | 1,775 (31%) |
| Got by | 1,872 (32%) |
| Found it difficult | 1,712 (30%) |
| Found it very difficult | 369 (6.4%) |
| Missing | 48 (0.8%) |
| **Abuse** |  |
| Yes | 905 (16%) |
| No | 4,604 (80%) |
| Missing | 267 (4.6%) |
| **Outsider growing up** |  |
| Yes | 772 (13%) |
| No | 4,897 (85%) |
| Missing | 107 (1.9%) |
| **Self-rated health growing up** |  |
| Excellent | 1,860 (32%) |
| Very good | 1,350 (23%) |
| Good | 1,677 (29%) |
| Fair | 743 (13%) |
| Poor | 133 (2.3%) |
| Missing | 14 (0.2%) |
| **Immigration status** |  |
| Born in this country | 5,517 (96%) |
| Born in another country | 108 (1.9%) |
| Missing | 151 (2.6%) |
| **Age 12 religious service attendance** |  |
| At least 1/week | 2,514 (44%) |
| 1-3/month | 1,162 (20%) |
| <1/month | 1,087 (19%) |
| Never | 944 (16%) |
| Missing | 69 (1.2%) |
| **Age group** |  |
| 1998-2005; current age: 18-24 | 986 (17%) |
| 1993-1998; age: 25-29 | 623 (11%) |
| 1983-1993; age: 30-39 | 1,312 (23%) |
| 1973-1983; age 40-49 | 1,027 (18%) |
| 1963-1973; age 50-59 | 873 (15%) |
| 1953-1963; age 60-69 | 611 (11%) |
| 1943 or earlier; age 80+ | 68 (1.2%) |
| 1943-1953; age 70-79 | 277 (4.8%) |
| Missing | 0 (0%) |
| **Gender** |  |
| Male | 2,755 (48%) |
| Female | 2,997 (52%) |
| Other | 3 (<0.1%) |
| Missing | 21 (0.4%) |
| **Religious affiliation at age 12** |  |
| Baha’i | 1 (<0.1%) |
| Buddhism | 1 (<0.1%) |
| Christianity | 5,337 (92%) |
| Hinduism | 1 (<0.1%) |
| Islam | 6 (<0.1%) |
| Judaism | 8 (0.1%) |
| No religion/Atheist/Agnostic | 328 (5.7%) |
| Primal, Animist, or Folk religion | 2 (<0.1%) |
| Shinto | 2 (<0.1%) |
| Sikhism | 4 (<0.1%) |
| Some other religion | 7 (0.1%) |
| Taoism | 5 (<0.1%) |
| Missing | 74 (1.3%) |
| **Race and ethnicity** |  |
| Black | 108 (1.9%) |
| Indigenous | 594 (10%) |
| Mestizo | 2,762 (48%) |
| Mulatto | 63 (1.1%) |
| Other | 339 (5.9%) |
| White | 1,116 (19%) |
| Missing | 794 (14%) |
| ^1^n (%) | |

***Table S11b. Regression of self-rated hope on childhood predictors for Mexico***

| Variable | Category | Estimate | SE | 95% CI | Global p-value |
| --- | --- | --- | --- | --- | --- |
| Relationship with mother (Ref: Very bad/Somewhat bad) | Very good/Somewhat good | 0.29 | 0.16 | (-0.02, 0.60) | <.001 |
| Relationship with father (Ref: Very bad/Somewhat bad) | Very good/Somewhat good | 0.10 | 0.10 | (-0.09, 0.30) | 0.007 |
| Parent marital status (Ref: Parents married) | No, divorced | 0.00 | 0.11 | (-0.22, 0.22) | 0.998 |
|  | Never | 0.00 | 0.09 | (-0.17, 0.17) |  |
|  | No, one or both of them had died | 0.14 | 0.17 | (-0.19, 0.47) |  |
| Subjective financial status of family growing up (Ref: Got by) | Lived comfortably | 0.07 | 0.08 | (-0.08, 0.22) | <.001 |
|  | Found it difficult | 0.21 | 0.07 | (0.07, 0.35) |  |
|  | Found it very difficult | 0.18 | 0.14 | (-0.09, 0.45) |  |
| Abuse (Ref: No) | Yes | 0.01 | 0.08 | (-0.14, 0.16) | 0.999 |
| Outsider growing up (Ref: No) | Yes | -0.05 | 0.09 | (-0.23, 0.12) | 0.816 |
| Self-rated health growing up (Ref: Good) | Excellent | 0.22 | 0.08 | (0.07, 0.37) | <.001 |
|  | Very good | 0.01 | 0.07 | (-0.13, 0.16) |  |
|  | Fair | -0.12 | 0.11 | (-0.34, 0.10) |  |
|  | Poor | -0.08 | 0.22 | (-0.51, 0.36) |  |
| Immigration status (Ref: Born in this country) | Born in another country | -0.79 | 0.28 | (-1.34, -0.24) | <.001 |
| Age 12 religious service attendance (Ref: Never) | At least 1/week | 0.28 | 0.09 | (0.10, 0.46) | <.001 |
|  | 1-3/month | 0.06 | 0.11 | (-0.15, 0.27) |  |
|  | <1/month | 0.14 | 0.11 | (-0.07, 0.35) |  |
| Age group (Ref: 1998-2005; current age: 18-24) | 1993-1998; age: 25-29 | 0.05 | 0.11 | (-0.17, 0.28) | <.001 |
|  | 1983-1993; age: 30-39 | 0.09 | 0.09 | (-0.08, 0.27) |  |
|  | 1973-1983; age 40-49 | 0.13 | 0.10 | (-0.07, 0.33) |  |
|  | 1963-1973; age 50-59 | 0.29 | 0.09 | (0.11, 0.47) |  |
|  | 1953-1963; age 60-69 | 0.14 | 0.11 | (-0.07, 0.36) |  |
|  | 1943 or earlier; age 80+ | -0.76 | 0.41 | (-1.58, 0.05) |  |
|  | 1943-1953; age 70-79 | -0.02 | 0.16 | (-0.32, 0.29) |  |
| Gender (Ref: Male) | Female | 0.14 | 0.06 | (0.02, 0.26) | <.001 |
|  | Other | -0.97 | 0.74 | (-2.42, 0.47) |  |
| Religious affiliation at age 12 (Ref: No religion/Atheist/Agnostic) | Christianity | 0.15 | 0.15 | (-0.14, 0.45) | 0.001 |
|  | Some other religion | 0.75 | 0.31 | (0.14, 1.37) |  |
| Race and ethnicity plurality (Ref: Majority) | Minority | 0.06 | 0.06 | (-0.06, 0.17) | 0.34 |

***Table S11c. Sensitivity to unmeasured confounding of childhood predictors in Mexico***

| Variable | Category | E-value for Estimate | E-value for 95% CI |
| --- | --- | --- | --- |
| Relationship with mother (Ref: Very bad/Somewhat bad)) | Very good/Somewhat good | 1.66 | 1.00 |
| Relationship with father (Ref: Very bad/Somewhat bad)) | Very good/Somewhat good | 1.32 | 1.00 |
| Parent marital status (Ref: Parents married) | No, divorced | 1.02 | 1.00 |
|  | Never | 1.02 | 1.00 |
|  | No, one or both of them had died | 1.39 | 1.00 |
| Subjective financial status of family growing up (Ref: Got by) | Lived comfortably | 1.25 | 1.00 |
|  | Found it difficult | 1.51 | 1.25 |
|  | Found it very difficult | 1.46 | 1.00 |
| Abuse (Ref: No) | Yes | 1.07 | 1.00 |
| Outsider growing up (Ref: No) | Yes | 1.21 | 1.00 |
| Self-rated health growing up (Ref: Good) | Excellent | 1.53 | 1.24 |
|  | Very good | 1.09 | 1.00 |
|  | Fair | 1.35 | 1.00 |
|  | Poor | 1.27 | 1.00 |
| Immigration status (Ref: Born in this country) | Born in another country | 2.55 | 1.56 |
| Age 12 religious service attendance (Ref: Never) | At least 1/week | 1.64 | 1.31 |
|  | 1-3/month | 1.23 | 1.00 |
|  | <1/month | 1.40 | 1.00 |
| Age group (Ref: 1998-2005; current age: 18-24) | 1993-1998; age: 25-29 | 1.21 | 1.00 |
|  | 1983-1993; age: 30-39 | 1.30 | 1.00 |
|  | 1973-1983; age 40-49 | 1.37 | 1.00 |
|  | 1963-1973; age 50-59 | 1.65 | 1.34 |
|  | 1953-1963; age 60-69 | 1.40 | 1.00 |
|  | 1943 or earlier; age 80+ | 2.51 | 1.00 |
|  | 1943-1953; age 70-79 | 1.11 | 1.00 |
| Gender (Ref: Male) | Female | 1.39 | 1.12 |
|  | Other | 2.94 | 1.00 |
| Religious affiliation at age 12 (Ref: No religion/Atheist/Agnostic) | Christianity | 1.42 | 1.00 |
|  | Some other religion | 2.49 | 1.39 |
| Race and ethnicity plurality (Ref: Majority) | Minority | 1.22 | 1.00 |

***Table S12a. Nationally representative descriptive statistics for Nigeria***

| **Characteristic** | **N = 6,827**^1^ |
| --- | --- |
| **Relationship with mother** |  |
| Very good | 5,986 (88%) |
| Somewhat good | 648 (9.5%) |
| Somewhat bad | 62 (0.9%) |
| Very bad | 18 (0.3%) |
| Does not apply | 104 (1.5%) |
| Missing | 9 (0.1%) |
| **Relationship with father** |  |
| Very good | 5,578 (82%) |
| Somewhat good | 924 (14%) |
| Somewhat bad | 76 (1.1%) |
| Very bad | 43 (0.6%) |
| Does not apply | 177 (2.6%) |
| Missing | 29 (0.4%) |
| **Parent marital status** |  |
| Yes, married | 5,568 (82%) |
| No, divorced | 307 (4.5%) |
| Never married | 335 (4.9%) |
| No, one or both of them had died | 462 (6.8%) |
| Missing | 154 (2.3%) |
| **Subjective financial status of family growing up** |  |
| Lived comfortably | 2,192 (32%) |
| Got by | 2,381 (35%) |
| Found it difficult | 1,661 (24%) |
| Found it very difficult | 563 (8.3%) |
| Missing | 29 (0.4%) |
| **Abuse** |  |
| Yes | 880 (13%) |
| No | 5,851 (86%) |
| Missing | 96 (1.4%) |
| **Outsider growing up** |  |
| Yes | 669 (9.8%) |
| No | 6,059 (89%) |
| Missing | 99 (1.5%) |
| **Self-rated health growing up** |  |
| Excellent | 2,644 (39%) |
| Very good | 2,613 (38%) |
| Good | 1,152 (17%) |
| Fair | 306 (4.5%) |
| Poor | 98 (1.4%) |
| Missing | 14 (0.2%) |
| **Immigration status** |  |
| Born in this country | 6,779 (99%) |
| Born in another country | 47 (0.7%) |
| Missing | 1 (<0.1%) |
| **Age 12 religious service attendance** |  |
| At least 1/week | 5,907 (87%) |
| 1-3/month | 600 (8.8%) |
| <1/month | 136 (2.0%) |
| Never | 138 (2.0%) |
| Missing | 45 (0.7%) |
| **Age group** |  |
| 1998-2005; current age: 18-24 | 1,533 (22%) |
| 1993-1998; age: 25-29 | 1,193 (17%) |
| 1983-1993; age: 30-39 | 1,943 (28%) |
| 1973-1983; age 40-49 | 1,059 (16%) |
| 1963-1973; age 50-59 | 619 (9.1%) |
| 1953-1963; age 60-69 | 296 (4.3%) |
| 1943 or earlier; age 80+ | 50 (0.7%) |
| 1943-1953; age 70-79 | 133 (2.0%) |
| Missing | 0 (0%) |
| **Gender** |  |
| Male | 3,371 (49%) |
| Female | 3,456 (51%) |
| Other | 0 (<0.1%) |
| Missing | 0 (0%) |
| **Religious affiliation at age 12** |  |
| Buddhism | 0 (<0.1%) |
| Christianity | 3,463 (51%) |
| Confucianism | 0 (<0.1%) |
| Islam | 3,314 (49%) |
| No religion/Atheist/Agnostic | 19 (0.3%) |
| Primal, Animist, or Folk religion | 17 (0.3%) |
| Missing | 14 (0.2%) |
| **Race and ethnicity** |  |
| Edo | 116 (1.7%) |
| Efik | 48 (0.7%) |
| Fulani | 266 (3.9%) |
| Hausa | 2,342 (34%) |
| Ibibio | 180 (2.6%) |
| Idoma | 61 (0.9%) |
| Igala | 77 (1.1%) |
| Igbo (Ibo) | 1,111 (16%) |
| Ijaw | 110 (1.6%) |
| Kanuri | 31 (0.5%) |
| Other | 1,014 (15%) |
| Tiv | 198 (2.9%) |
| Urhobo | 38 (0.6%) |
| Yoruba | 1,230 (18%) |
| Missing | 4 (<0.1%) |
| ^1^n (%) | |

***Table S12b. Regression of self-rated hope on childhood predictors for Nigeria***

| Variable | Category | Estimate | SE | 95% CI | Global p-value |
| --- | --- | --- | --- | --- | --- |
| Relationship with mother (Ref: Very bad/Somewhat bad) | Very good/Somewhat good | -0.78 | 0.29 | (-1.36, -0.21) | <.001 |
| Relationship with father (Ref: Very bad/Somewhat bad) | Very good/Somewhat good | -0.03 | 0.22 | (-0.46, 0.40) | 0.996 |
| Parent marital status (Ref: Parents married) | No, divorced | -0.42 | 0.20 | (-0.81, -0.04) | <.001 |
|  | Never | -0.13 | 0.14 | (-0.40, 0.14) |  |
|  | No, one or both of them had died | -0.50 | 0.16 | (-0.81, -0.19) |  |
| Subjective financial status of family growing up (Ref: Got by) | Lived comfortably | -0.02 | 0.10 | (-0.21, 0.17) | 1 |
|  | Found it difficult | 0.03 | 0.09 | (-0.15, 0.21) |  |
|  | Found it very difficult | -0.09 | 0.15 | (-0.39, 0.22) |  |
| Abuse (Ref: No) | Yes | -0.06 | 0.11 | (-0.27, 0.15) | 0.826 |
| Outsider growing up (Ref: No) | Yes | -0.08 | 0.12 | (-0.32, 0.16) | 0.75 |
| Self-rated health growing up (Ref: Good) | Excellent | -0.03 | 0.11 | (-0.24, 0.18) | 0.886 |
|  | Very good | -0.11 | 0.10 | (-0.32, 0.09) |  |
|  | Fair | -0.10 | 0.19 | (-0.46, 0.27) |  |
|  | Poor | -0.32 | 0.28 | (-0.87, 0.23) |  |
| Immigration status (Ref: Born in this country) | Born in another country | -0.19 | 0.34 | (-0.86, 0.48) | 0.856 |
| Age 12 religious service attendance (Ref: Never) | At least 1/week | 0.24 | 0.26 | (-0.27, 0.75) | 0.031 |
|  | 1-3/month | 0.11 | 0.28 | (-0.45, 0.67) |  |
|  | <1/month | -0.25 | 0.33 | (-0.89, 0.40) |  |
| Age group (Ref: 1998-2005; current age: 18-24) | 1993-1998; age: 25-29 | -0.06 | 0.08 | (-0.23, 0.10) | 1 |
|  | 1983-1993; age: 30-39 | -0.10 | 0.09 | (-0.28, 0.08) |  |
|  | 1973-1983; age 40-49 | -0.03 | 0.12 | (-0.26, 0.20) |  |
|  | 1963-1973; age 50-59 | 0.08 | 0.16 | (-0.23, 0.38) |  |
|  | 1953-1963; age 60-69 | -0.12 | 0.25 | (-0.61, 0.36) |  |
|  | 1943 or earlier; age 80+ | -0.38 | 0.68 | (-1.70, 0.94) |  |
|  | 1943-1953; age 70-79 | -0.08 | 0.57 | (-1.20, 1.04) |  |
| Gender (Ref: Male) | Female | -0.01 | 0.07 | (-0.15, 0.13) | 0.039 |
|  | Other | 0.25 | 0.14 | (-0.02, 0.52) |  |
| Religious affiliation at age 12 (Ref: Christianity) | Islam | -0.23 | 0.10 | (-0.43, -0.03) | <.001 |
|  | Some other religion | 0.74 | 0.21 | (0.33, 1.15) |  |
| Race and ethnicity plurality (Ref: Majority) | Minority | 0.08 | 0.12 | (-0.15, 0.30) | 0.757 |

***Table S12c. Sensitivity to unmeasured confounding of childhood predictors in Nigeria***

| Variable | Category | E-value for Estimate | E-value for 95% CI |
| --- | --- | --- | --- |
| Relationship with mother (Ref: Very bad/Somewhat bad)) | Very good/Somewhat good | 2.26 | 1.45 |
| Relationship with father (Ref: Very bad/Somewhat bad)) | Very good/Somewhat good | 1.13 | 1.00 |
| Parent marital status (Ref: Parents married) | No, divorced | 1.75 | 1.16 |
|  | Never | 1.33 | 1.00 |
|  | No, one or both of them had died | 1.85 | 1.42 |
| Subjective financial status of family growing up (Ref: Got by) | Lived comfortably | 1.10 | 1.00 |
|  | Found it difficult | 1.13 | 1.00 |
|  | Found it very difficult | 1.25 | 1.00 |
| Abuse (Ref: No) | Yes | 1.20 | 1.00 |
| Outsider growing up (Ref: No) | Yes | 1.24 | 1.00 |
| Self-rated health growing up (Ref: Good) | Excellent | 1.13 | 1.00 |
|  | Very good | 1.29 | 1.00 |
|  | Fair | 1.27 | 1.00 |
|  | Poor | 1.60 | 1.00 |
| Immigration status (Ref: Born in this country) | Born in another country | 1.42 | 1.00 |
| Age 12 religious service attendance (Ref: Never) | At least 1/week | 1.49 | 1.00 |
|  | 1-3/month | 1.29 | 1.00 |
|  | <1/month | 1.50 | 1.00 |
| Age group (Ref: 1998-2005; current age: 18-24) | 1993-1998; age: 25-29 | 1.20 | 1.00 |
|  | 1983-1993; age: 30-39 | 1.27 | 1.00 |
|  | 1973-1983; age 40-49 | 1.14 | 1.00 |
|  | 1963-1973; age 50-59 | 1.23 | 1.00 |
|  | 1953-1963; age 60-69 | 1.31 | 1.00 |
|  | 1943 or earlier; age 80+ | 1.68 | 1.00 |
|  | 1943-1953; age 70-79 | 1.23 | 1.00 |
| Gender (Ref: Male) | Female | 1.07 | 1.00 |
|  | Other | 1.51 | 1.00 |
| Religious affiliation at age 12 (Ref: Christianity) | Some other religion | 2.20 | 1.62 |
| Race and ethnicity plurality (Ref: Majority) | Minority | 1.23 | 1.00 |
|  | Islam | 1.47 | 1.14 |

***Table S13a. Nationally representative descriptive statistics for Philippines***

| **Characteristic** | **N = 5,292**^1^ |
| --- | --- |
| **Relationship with mother** |  |
| Very good | 3,333 (63%) |
| Somewhat good | 1,703 (32%) |
| Somewhat bad | 124 (2.3%) |
| Very bad | 39 (0.7%) |
| Does not apply | 59 (1.1%) |
| Missing | 35 (0.7%) |
| **Relationship with father** |  |
| Very good | 3,443 (65%) |
| Somewhat good | 1,429 (27%) |
| Somewhat bad | 159 (3.0%) |
| Very bad | 58 (1.1%) |
| Does not apply | 108 (2.0%) |
| Missing | 95 (1.8%) |
| **Parent marital status** |  |
| Yes, married | 4,575 (86%) |
| No, divorced | 64 (1.2%) |
| Never married | 517 (9.8%) |
| No, one or both of them had died | 51 (1.0%) |
| Missing | 85 (1.6%) |
| **Subjective financial status of family growing up** |  |
| Lived comfortably | 937 (18%) |
| Got by | 3,006 (57%) |
| Found it difficult | 1,055 (20%) |
| Found it very difficult | 291 (5.5%) |
| Missing | 3 (<0.1%) |
| **Abuse** |  |
| Yes | 420 (7.9%) |
| No | 4,837 (91%) |
| Missing | 35 (0.7%) |
| **Outsider growing up** |  |
| Yes | 395 (7.5%) |
| No | 4,884 (92%) |
| Missing | 13 (0.2%) |
| **Self-rated health growing up** |  |
| Excellent | 1,041 (20%) |
| Very good | 559 (11%) |
| Good | 2,174 (41%) |
| Fair | 1,246 (24%) |
| Poor | 272 (5.1%) |
| Missing | 0 (<0.1%) |
| **Immigration status** |  |
| Born in this country | 5,284 (100%) |
| Born in another country | 8 (0.1%) |
| Missing | 0 (0%) |
| **Age 12 religious service attendance** |  |
| At least 1/week | 2,453 (46%) |
| 1-3/month | 1,699 (32%) |
| <1/month | 892 (17%) |
| Never | 201 (3.8%) |
| Missing | 47 (0.9%) |
| **Age group** |  |
| 1998-2005; current age: 18-24 | 1,073 (20%) |
| 1993-1998; age: 25-29 | 695 (13%) |
| 1983-1993; age: 30-39 | 1,160 (22%) |
| 1973-1983; age 40-49 | 972 (18%) |
| 1963-1973; age 50-59 | 732 (14%) |
| 1953-1963; age 60-69 | 495 (9.4%) |
| 1943 or earlier; age 80+ | 23 (0.4%) |
| 1943-1953; age 70-79 | 143 (2.7%) |
| Missing | 0 (0%) |
| **Gender** |  |
| Male | 2,625 (50%) |
| Female | 2,643 (50%) |
| Other | 13 (0.2%) |
| Missing | 11 (0.2%) |
| **Religious affiliation at age 12** |  |
| Baha’i | 1 (<0.1%) |
| Buddhism | 1 (<0.1%) |
| Christianity | 4,968 (94%) |
| Islam | 276 (5.2%) |
| No religion/Atheist/Agnostic | 9 (0.2%) |
| Primal, Animist, or Folk religion | 14 (0.3%) |
| Sikhism | 4 (<0.1%) |
| Some other religion | 9 (0.2%) |
| Missing | 11 (0.2%) |
| **Race and ethnicity** |  |
| Aeta | 1 (<0.1%) |
| Badjao | 2 (<0.1%) |
| Bicolano/Bikolano | 300 (5.7%) |
| Cebuano | 656 (12%) |
| Chinese-Filipino | 3 (<0.1%) |
| Igorot | 42 (0.8%) |
| Ilocano/Ilokano | 429 (8.1%) |
| Ilonggo/Hiligaynon | 428 (8.1%) |
| Kapampangan | 107 (2.0%) |
| Maguindanaoan | 84 (1.6%) |
| Mangyan | 2 (<0.1%) |
| Maranao | 39 (0.7%) |
| Masbateno | 54 (1.0%) |
| Other | 244 (4.6%) |
| Pangasinense | 107 (2.0%) |
| Tagalog | 1,691 (32%) |
| Tausug | 94 (1.8%) |
| Visayan/Bisaya | 739 (14%) |
| Waray | 216 (4.1%) |
| Zamboangueno | 51 (1.0%) |
| Missing | 3 (<0.1%) |
| ^1^n (%) | |

***Table S13b. Regression of self-rated hope on childhood predictors for Philippines***

| Variable | Category | Estimate | SE | 95% CI | Global p-value |
| --- | --- | --- | --- | --- | --- |
| Relationship with mother (Ref: Very bad/Somewhat bad) | Very good/Somewhat good | 0.33 | 0.20 | (-0.07, 0.73) | <.001 |
| Relationship with father (Ref: Very bad/Somewhat bad) | Very good/Somewhat good | -0.04 | 0.14 | (-0.31, 0.22) | 0.808 |
| Parent marital status (Ref: Parents married) | No, divorced | -0.58 | 0.42 | (-1.39, 0.24) | 0.749 |
|  | Never | 0.04 | 0.10 | (-0.16, 0.23) |  |
|  | No, one or both of them had died | 0.09 | 0.27 | (-0.44, 0.62) |  |
| Subjective financial status of family growing up (Ref: Got by) | Lived comfortably | 0.15 | 0.08 | (-0.01, 0.31) | 0.005 |
|  | Found it difficult | 0.00 | 0.08 | (-0.15, 0.15) |  |
|  | Found it very difficult | 0.30 | 0.16 | (-0.02, 0.61) |  |
| Abuse (Ref: No) | Yes | -0.32 | 0.14 | (-0.60, -0.03) | <.001 |
| Outsider growing up (Ref: No) | Yes | -0.30 | 0.15 | (-0.60, 0.00) | 0.001 |
| Self-rated health growing up (Ref: Good) | Excellent | 0.11 | 0.08 | (-0.06, 0.27) | <.001 |
|  | Very good | -0.12 | 0.10 | (-0.31, 0.07) |  |
|  | Fair | -0.23 | 0.07 | (-0.38, -0.08) |  |
|  | Poor | -0.37 | 0.17 | (-0.70, -0.05) |  |
| Immigration status (Ref: Born in this country) | Born in another country | -1.33 | 0.63 | (-2.57, -0.10) | <.001 |
| Age 12 religious service attendance (Ref: Never) | At least 1/week | 0.72 | 0.29 | (0.16, 1.28) | <.001 |
|  | 1-3/month | 0.62 | 0.30 | (0.04, 1.20) |  |
|  | <1/month | 0.63 | 0.30 | (0.05, 1.21) |  |
| Age group (Ref: 1998-2005; current age: 18-24) | 1993-1998; age: 25-29 | 0.26 | 0.10 | (0.07, 0.45) | <.001 |
|  | 1983-1993; age: 30-39 | 0.25 | 0.08 | (0.08, 0.41) |  |
|  | 1973-1983; age 40-49 | 0.12 | 0.10 | (-0.07, 0.32) |  |
|  | 1963-1973; age 50-59 | -0.04 | 0.12 | (-0.28, 0.19) |  |
|  | 1953-1963; age 60-69 | -0.22 | 0.14 | (-0.50, 0.06) |  |
|  | 1943 or earlier; age 80+ | 0.13 | 0.49 | (-0.82, 1.08) |  |
|  | 1943-1953; age 70-79 | -0.45 | 0.23 | (-0.90, 0.00) |  |
| Gender (Ref: Male) | Female | 0.07 | 0.06 | (-0.04, 0.19) | 0.087 |
|  | Other | -0.56 | 0.48 | (-1.50, 0.38) |  |
| Religious affiliation at age 12 (Ref: Christianity) | Islam | -0.10 | 0.18 | (-0.46, 0.26) | 0.976 |
|  | Some other religion | -0.27 | 0.44 | (-1.14, 0.60) |  |
| Race and ethnicity plurality (Ref: Majority) | Minority | -0.21 | 0.06 | (-0.34, -0.08) | <.001 |

***Table S13c. Sensitivity to unmeasured confounding of childhood predictors in Philippines***

| Variable | Category | E-value for Estimate | E-value for 95% CI |
| --- | --- | --- | --- |
| Relationship with mother (Ref: Very bad/Somewhat bad)) | Very good/Somewhat good | 1.63 | 1.00 |
| Relationship with father (Ref: Very bad/Somewhat bad)) | Very good/Somewhat good | 1.17 | 1.00 |
| Parent marital status (Ref: Parents married) | No, divorced | 2.00 | 1.00 |
|  | Never | 1.15 | 1.00 |
|  | No, one or both of them had died | 1.27 | 1.00 |
| Subjective financial status of family growing up (Ref: Got by) | Lived comfortably | 1.37 | 1.00 |
|  | Found it difficult | 1.02 | 1.00 |
|  | Found it very difficult | 1.59 | 1.00 |
| Abuse (Ref: No) | Yes | 1.62 | 1.15 |
| Outsider growing up (Ref: No) | Yes | 1.59 | 1.00 |
| Self-rated health growing up (Ref: Good) | Excellent | 1.29 | 1.00 |
|  | Very good | 1.31 | 1.00 |
|  | Fair | 1.49 | 1.25 |
|  | Poor | 1.70 | 1.19 |
| Immigration status (Ref: Born in this country) | Born in another country | 3.30 | 1.29 |
| Age 12 religious service attendance (Ref: Never) | At least 1/week | 2.22 | 1.38 |
|  | 1-3/month | 2.06 | 1.17 |
|  | <1/month | 2.07 | 1.18 |
| Age group (Ref: 1998-2005; current age: 18-24) | 1993-1998; age: 25-29 | 1.54 | 1.23 |
|  | 1983-1993; age: 30-39 | 1.52 | 1.26 |
|  | 1973-1983; age 40-49 | 1.33 | 1.00 |
|  | 1963-1973; age 50-59 | 1.17 | 1.00 |
|  | 1953-1963; age 60-69 | 1.48 | 1.00 |
|  | 1943 or earlier; age 80+ | 1.33 | 1.00 |
|  | 1943-1953; age 70-79 | 1.81 | 1.00 |
| Gender (Ref: Male) | Female | 1.23 | 1.00 |
|  | Other | 1.98 | 1.00 |
| Religious affiliation at age 12 (Ref: Christianity) | Some other religion | 1.55 | 1.00 |
| Race and ethnicity plurality (Ref: Majority) | Minority | 1.46 | 1.25 |
|  | Islam | 1.28 | 1.00 |

***Table S14a. Nationally representative descriptive statistics for Poland***

| **Characteristic** | **N = 10,389**^1^ |
| --- | --- |
| **Relationship with mother** |  |
| Very good | 4,879 (47%) |
| Somewhat good | 4,973 (48%) |
| Somewhat bad | 285 (2.7%) |
| Very bad | 58 (0.6%) |
| Does not apply | 80 (0.8%) |
| Missing | 112 (1.1%) |
| **Relationship with father** |  |
| Very good | 4,231 (41%) |
| Somewhat good | 4,984 (48%) |
| Somewhat bad | 516 (5.0%) |
| Very bad | 78 (0.7%) |
| Does not apply | 407 (3.9%) |
| Missing | 173 (1.7%) |
| **Parent marital status** |  |
| Yes, married | 8,972 (86%) |
| No, divorced | 587 (5.7%) |
| Never married | 193 (1.9%) |
| No, one or both of them had died | 313 (3.0%) |
| Missing | 324 (3.1%) |
| **Subjective financial status of family growing up** |  |
| Lived comfortably | 1,384 (13%) |
| Got by | 6,257 (60%) |
| Found it difficult | 2,133 (21%) |
| Found it very difficult | 509 (4.9%) |
| Missing | 106 (1.0%) |
| **Abuse** |  |
| Yes | 325 (3.1%) |
| No | 10,009 (96%) |
| Missing | 55 (0.5%) |
| **Outsider growing up** |  |
| Yes | 490 (4.7%) |
| No | 9,615 (93%) |
| Missing | 284 (2.7%) |
| **Self-rated health growing up** |  |
| Excellent | 2,676 (26%) |
| Very good | 5,371 (52%) |
| Good | 1,779 (17%) |
| Fair | 406 (3.9%) |
| Poor | 123 (1.2%) |
| Missing | 34 (0.3%) |
| **Immigration status** |  |
| Born in this country | 10,258 (99%) |
| Born in another country | 108 (1.0%) |
| Missing | 23 (0.2%) |
| **Age 12 religious service attendance** |  |
| At least 1/week | 4,751 (46%) |
| 1-3/month | 2,689 (26%) |
| <1/month | 2,161 (21%) |
| Never | 354 (3.4%) |
| Missing | 434 (4.2%) |
| **Age group** |  |
| 1998-2005; current age: 18-24 | 955 (9.2%) |
| 1993-1998; age: 25-29 | 761 (7.3%) |
| 1983-1993; age: 30-39 | 2,159 (21%) |
| 1973-1983; age 40-49 | 1,956 (19%) |
| 1963-1973; age 50-59 | 1,670 (16%) |
| 1953-1963; age 60-69 | 1,909 (18%) |
| 1943 or earlier; age 80+ | 145 (1.4%) |
| 1943-1953; age 70-79 | 833 (8.0%) |
| Missing | 1 (<0.1%) |
| **Gender** |  |
| Male | 4,974 (48%) |
| Female | 5,387 (52%) |
| Other | 3 (<0.1%) |
| Missing | 26 (0.2%) |
| **Religious affiliation at age 12** |  |
| Buddhism | 2 (<0.1%) |
| Christianity | 9,861 (95%) |
| Islam | 3 (<0.1%) |
| No religion/Atheist/Agnostic | 482 (4.6%) |
| Primal, Animist, or Folk religion | 5 (<0.1%) |
| Sikhism | 1 (<0.1%) |
| Missing | 35 (0.3%) |
| **Race and ethnicity** |  |
| Belarussian | 2 (<0.1%) |
| German | 4 (<0.1%) |
| Kashubians | 3 (<0.1%) |
| Other | 4 (<0.1%) |
| Polish | 10,309 (99%) |
| Silesia | 14 (0.1%) |
| Ukrainian | 38 (0.4%) |
| Missing | 14 (0.1%) |
| ^1^n (%) | |

***Table S14b. Regression of self-rated hope on childhood predictors for Poland***

| Variable | Category | Estimate | SE | 95% CI | Global p-value |
| --- | --- | --- | --- | --- | --- |
| Relationship with mother (Ref: Very bad/Somewhat bad) | Very good/Somewhat good | 0.35 | 0.28 | (-0.19, 0.89) | <.001 |
| Relationship with father (Ref: Very bad/Somewhat bad) | Very good/Somewhat good | 0.15 | 0.22 | (-0.28, 0.59) | 0.156 |
| Parent marital status (Ref: Parents married) | No, divorced | -0.65 | 0.13 | (-0.89, -0.40) | <.001 |
|  | Never | -1.04 | 0.39 | (-1.82, -0.27) |  |
|  | No, one or both of them had died | -0.25 | 0.24 | (-0.71, 0.22) |  |
| Subjective financial status of family growing up (Ref: Got by) | Lived comfortably | 0.04 | 0.09 | (-0.13, 0.21) | 1 |
|  | Found it difficult | -0.01 | 0.08 | (-0.16, 0.15) |  |
|  | Found it very difficult | -0.06 | 0.22 | (-0.48, 0.37) |  |
| Abuse (Ref: No) | Yes | -0.25 | 0.22 | (-0.69, 0.18) | 0.155 |
| Outsider growing up (Ref: No) | Yes | -0.22 | 0.19 | (-0.60, 0.15) | 0.032 |
| Self-rated health growing up (Ref: Good) | Excellent | 0.38 | 0.12 | (0.14, 0.62) | <.001 |
|  | Very good | 0.13 | 0.10 | (-0.07, 0.33) |  |
|  | Fair | -0.41 | 0.25 | (-0.90, 0.09) |  |
|  | Poor | 0.48 | 0.44 | (-0.38, 1.34) |  |
| Immigration status (Ref: Born in this country) | Born in another country | -0.38 | 0.48 | (-1.32, 0.55) | 0.565 |
| Age 12 religious service attendance (Ref: Never) | At least 1/week | 0.77 | 0.20 | (0.38, 1.16) | <.001 |
|  | 1-3/month | 0.48 | 0.20 | (0.09, 0.87) |  |
|  | <1/month | 0.23 | 0.18 | (-0.13, 0.59) |  |
| Age group (Ref: 1998-2005; current age: 18-24) | 1993-1998; age: 25-29 | -0.25 | 0.12 | (-0.48, -0.02) | <.001 |
|  | 1983-1993; age: 30-39 | -0.38 | 0.10 | (-0.58, -0.19) |  |
|  | 1973-1983; age 40-49 | -0.54 | 0.11 | (-0.75, -0.33) |  |
|  | 1963-1973; age 50-59 | -0.69 | 0.13 | (-0.93, -0.44) |  |
|  | 1953-1963; age 60-69 | -0.58 | 0.13 | (-0.84, -0.32) |  |
|  | 1943 or earlier; age 80+ | -1.05 | 0.38 | (-1.79, -0.31) |  |
|  | 1943-1953; age 70-79 | -0.44 | 0.19 | (-0.81, -0.08) |  |
| Gender (Ref: Male) | Female | 0.11 | 0.06 | (0.00, 0.22) | <.001 |
|  | Other | -1.59 | 1.07 | (-3.69, 0.50) |  |
| Religious affiliation at age 12 (Ref: No religion/Atheist/Agnostic) | Christianity | -0.35 | 0.18 | (-0.71, 0.01) | <.001 |
|  | Some other religion | -1.29 | 0.63 | (-2.53, -0.05) |  |
| Race and ethnicity plurality (Ref: Majority) | Minority | 0.16 | 0.37 | (-0.58, 0.89) | 0.95 |

***Table S14c. Sensitivity to unmeasured confounding of childhood predictors in Poland***

| Variable | Category | E-value for Estimate | E-value for 95% CI |
| --- | --- | --- | --- |
| Relationship with mother (Ref: Very bad/Somewhat bad)) | Very good/Somewhat good | 1.71 | 1.00 |
| Relationship with father (Ref: Very bad/Somewhat bad)) | Very good/Somewhat good | 1.39 | 1.00 |
| Parent marital status (Ref: Parents married) | No, divorced | 2.20 | 1.80 |
|  | Never | 2.93 | 1.59 |
|  | No, one or both of them had died | 1.55 | 1.00 |
| Subjective financial status of family growing up (Ref: Got by) | Lived comfortably | 1.18 | 1.00 |
|  | Found it difficult | 1.06 | 1.00 |
|  | Found it very difficult | 1.22 | 1.00 |
| Abuse (Ref: No) | Yes | 1.56 | 1.00 |
| Outsider growing up (Ref: No) | Yes | 1.51 | 1.00 |
| Self-rated health growing up (Ref: Good) | Excellent | 1.76 | 1.38 |
|  | Very good | 1.36 | 1.00 |
|  | Fair | 1.80 | 1.00 |
|  | Poor | 1.92 | 1.00 |
| Immigration status (Ref: Born in this country) | Born in another country | 1.77 | 1.00 |
| Age 12 religious service attendance (Ref: Never) | At least 1/week | 2.40 | 1.76 |
|  | 1-3/month | 1.92 | 1.29 |
|  | <1/month | 1.52 | 1.00 |
| Age group (Ref: 1998-2005; current age: 18-24) | 1993-1998; age: 25-29 | 1.56 | 1.11 |
|  | 1983-1993; age: 30-39 | 1.77 | 1.45 |
|  | 1973-1983; age 40-49 | 2.02 | 1.69 |
|  | 1963-1973; age 50-59 | 2.27 | 1.86 |
|  | 1953-1963; age 60-69 | 2.09 | 1.67 |
|  | 1943 or earlier; age 80+ | 2.94 | 1.66 |
|  | 1943-1953; age 70-79 | 1.86 | 1.27 |
| Gender (Ref: Male) | Female | 1.32 | 1.00 |
|  | Other | 4.19 | 1.00 |
| Religious affiliation at age 12 (Ref: No religion/Atheist/Agnostic) | Christianity | 1.71 | 1.00 |
|  | Some other religion | 3.44 | 1.20 |
| Race and ethnicity plurality (Ref: Majority) | Minority | 1.40 | 1.00 |

***Table S15a. Nationally representative descriptive statistics for South Africa***

| **Characteristic** | **N = 2,651**^1^ |
| --- | --- |
| **Relationship with mother** |  |
| Very good | 2,186 (82%) |
| Somewhat good | 263 (9.9%) |
| Somewhat bad | 51 (1.9%) |
| Very bad | 39 (1.5%) |
| Does not apply | 90 (3.4%) |
| Missing | 21 (0.8%) |
| **Relationship with father** |  |
| Very good | 1,656 (62%) |
| Somewhat good | 333 (13%) |
| Somewhat bad | 86 (3.3%) |
| Very bad | 159 (6.0%) |
| Does not apply | 331 (12%) |
| Missing | 85 (3.2%) |
| **Parent marital status** |  |
| Yes, married | 1,321 (50%) |
| No, divorced | 131 (5.0%) |
| Never married | 904 (34%) |
| No, one or both of them had died | 140 (5.3%) |
| Missing | 155 (5.8%) |
| **Subjective financial status of family growing up** |  |
| Lived comfortably | 1,050 (40%) |
| Got by | 875 (33%) |
| Found it difficult | 432 (16%) |
| Found it very difficult | 289 (11%) |
| Missing | 5 (0.2%) |
| **Abuse** |  |
| Yes | 450 (17%) |
| No | 2,149 (81%) |
| Missing | 52 (2.0%) |
| **Outsider growing up** |  |
| Yes | 434 (16%) |
| No | 2,211 (83%) |
| Missing | 6 (0.2%) |
| **Self-rated health growing up** |  |
| Excellent | 1,225 (46%) |
| Very good | 590 (22%) |
| Good | 370 (14%) |
| Fair | 266 (10%) |
| Poor | 183 (6.9%) |
| Missing | 17 (0.6%) |
| **Immigration status** |  |
| Born in this country | 2,511 (95%) |
| Born in another country | 139 (5.2%) |
| Missing | 1 (<0.1%) |
| **Age 12 religious service attendance** |  |
| At least 1/week | 1,681 (63%) |
| 1-3/month | 552 (21%) |
| <1/month | 175 (6.6%) |
| Never | 217 (8.2%) |
| Missing | 26 (1.0%) |
| **Age group** |  |
| 1998-2005; current age: 18-24 | 461 (17%) |
| 1993-1998; age: 25-29 | 364 (14%) |
| 1983-1993; age: 30-39 | 655 (25%) |
| 1973-1983; age 40-49 | 522 (20%) |
| 1963-1973; age 50-59 | 309 (12%) |
| 1953-1963; age 60-69 | 195 (7.4%) |
| 1943 or earlier; age 80+ | 17 (0.6%) |
| 1943-1953; age 70-79 | 120 (4.5%) |
| Missing | 9 (0.3%) |
| **Gender** |  |
| Male | 1,288 (49%) |
| Female | 1,356 (51%) |
| Other | 2 (<0.1%) |
| Missing | 4 (0.2%) |
| **Religious affiliation at age 12** |  |
| Buddhism | 11 (0.4%) |
| Christianity | 2,323 (88%) |
| Hinduism | 2 (<0.1%) |
| Islam | 52 (2.0%) |
| No religion/Atheist/Agnostic | 107 (4.1%) |
| Primal, Animist, or Folk religion | 117 (4.4%) |
| Shinto | 2 (<0.1%) |
| Some other religion | 7 (0.3%) |
| Taoism | 1 (<0.1%) |
| Missing | 27 (1.0%) |
| **Race and ethnicity** |  |
| Asian/Indian | 6 (0.2%) |
| Black | 2,381 (90%) |
| Colored | 252 (9.5%) |
| Other | 1 (<0.1%) |
| White | 8 (0.3%) |
| Missing | 3 (0.1%) |
| ^1^n (%) | |

***Table S15b. Regression of self-rated hope on childhood predictors for South Africa***

| Variable | Category | Estimate | SE | 95% CI | Global p-value |
| --- | --- | --- | --- | --- | --- |
| Relationship with mother (Ref: Very bad/Somewhat bad) | Very good/Somewhat good | -0.05 | 0.25 | (-0.53, 0.44) | 0.974 |
| Relationship with father (Ref: Very bad/Somewhat bad) | Very good/Somewhat good | 0.02 | 0.17 | (-0.32, 0.35) | 0.922 |
| Parent marital status (Ref: Parents married) | No, divorced | 0.29 | 0.17 | (-0.05, 0.63) | 0.269 |
|  | Never | -0.08 | 0.12 | (-0.32, 0.16) |  |
|  | No, one or both of them had died | -0.02 | 0.25 | (-0.51, 0.47) |  |
| Subjective financial status of family growing up (Ref: Got by) | Lived comfortably | 0.00 | 0.12 | (-0.24, 0.24) | 1 |
|  | Found it difficult | -0.10 | 0.18 | (-0.46, 0.25) |  |
|  | Found it very difficult | 0.04 | 0.21 | (-0.37, 0.45) |  |
| Abuse (Ref: No) | Yes | -0.23 | 0.17 | (-0.56, 0.11) | 0.071 |
| Outsider growing up (Ref: No) | Yes | 0.09 | 0.15 | (-0.21, 0.39) | 0.828 |
| Self-rated health growing up (Ref: Good) | Excellent | 0.42 | 0.17 | (0.08, 0.76) | <.001 |
|  | Very good | 0.37 | 0.17 | (0.04, 0.70) |  |
|  | Fair | 0.50 | 0.22 | (0.07, 0.93) |  |
|  | Poor | -0.11 | 0.29 | (-0.68, 0.45) |  |
| Immigration status (Ref: Born in this country) | Born in another country | -0.17 | 0.24 | (-0.64, 0.30) | 0.673 |
| Age 12 religious service attendance (Ref: Never) | At least 1/week | 0.37 | 0.28 | (-0.18, 0.92) | 0.834 |
|  | 1-3/month | 0.38 | 0.28 | (-0.17, 0.93) |  |
|  | <1/month | 0.40 | 0.34 | (-0.26, 1.07) |  |
| Age group (Ref: 1998-2005; current age: 18-24) | 1993-1998; age: 25-29 | -0.09 | 0.16 | (-0.41, 0.23) | <.001 |
|  | 1983-1993; age: 30-39 | 0.03 | 0.15 | (-0.26, 0.32) |  |
|  | 1973-1983; age 40-49 | -0.38 | 0.18 | (-0.72, -0.03) |  |
|  | 1963-1973; age 50-59 | 0.09 | 0.20 | (-0.31, 0.49) |  |
|  | 1953-1963; age 60-69 | -0.24 | 0.27 | (-0.77, 0.29) |  |
|  | 1943 or earlier; age 80+ | 0.82 | 0.21 | (0.41, 1.22) |  |
|  | 1943-1953; age 70-79 | -0.19 | 0.36 | (-0.90, 0.53) |  |
| Gender (Ref: Male) | Female | -0.23 | 0.11 | (-0.44, -0.02) | <.001 |
|  | Other | -0.67 | 0.37 | (-1.39, 0.05) |  |
| Religious affiliation at age 12 (Ref: No religion/Atheist/Agnostic) | Christianity | -0.34 | 0.40 | (-1.13, 0.44) | 0.994 |
|  | Primal, Animist, or Folk religion | -0.26 | 0.45 | (-1.14, 0.63) |  |
|  | Some other religion | -0.36 | 0.48 | (-1.30, 0.58) |  |
| Race and ethnicity plurality (Ref: Majority) | Minority | -0.15 | 0.23 | (-0.60, 0.30) | 0.739 |

***Table S15c. Sensitivity to unmeasured confounding of childhood predictors in South Africa***

| Variable | Category | E-value for Estimate | E-value for 95% CI |
| --- | --- | --- | --- |
| Relationship with mother (Ref: Very bad/Somewhat bad)) | Very good/Somewhat good | 1.17 | 1.00 |
| Relationship with father (Ref: Very bad/Somewhat bad)) | Very good/Somewhat good | 1.10 | 1.00 |
| Parent marital status (Ref: Parents married) | No, divorced | 1.54 | 1.00 |
|  | Never | 1.23 | 1.00 |
|  | No, one or both of them had died | 1.11 | 1.00 |
| Subjective financial status of family growing up (Ref: Got by) | Lived comfortably | 1.03 | 1.00 |
|  | Found it difficult | 1.27 | 1.00 |
|  | Found it very difficult | 1.15 | 1.00 |
| Abuse (Ref: No) | Yes | 1.45 | 1.00 |
| Outsider growing up (Ref: No) | Yes | 1.25 | 1.00 |
| Self-rated health growing up (Ref: Good) | Excellent | 1.71 | 1.24 |
|  | Very good | 1.65 | 1.16 |
|  | Fair | 1.81 | 1.21 |
|  | Poor | 1.29 | 1.00 |
| Immigration status (Ref: Born in this country) | Born in another country | 1.38 | 1.00 |
| Age 12 religious service attendance (Ref: Never) | At least 1/week | 1.65 | 1.00 |
|  | 1-3/month | 1.66 | 1.00 |
|  | <1/month | 1.68 | 1.00 |
| Age group (Ref: 1998-2005; current age: 18-24) | 1993-1998; age: 25-29 | 1.25 | 1.00 |
|  | 1983-1993; age: 30-39 | 1.13 | 1.00 |
|  | 1973-1983; age 40-49 | 1.65 | 1.13 |
|  | 1963-1973; age 50-59 | 1.24 | 1.00 |
|  | 1953-1963; age 60-69 | 1.47 | 1.00 |
|  | 1943 or earlier; age 80+ | 2.25 | 1.70 |
|  | 1943-1953; age 70-79 | 1.39 | 1.00 |
| Gender (Ref: Male) | Female | 1.46 | 1.11 |
|  | Other | 2.04 | 1.00 |
| Religious affiliation at age 12 (Ref: No religion/Atheist/Agnostic) | Christianity | 1.61 | 1.00 |
|  | Some other religion | 1.63 | 1.00 |
| Race and ethnicity plurality (Ref: Majority) | Minority | 1.35 | 1.00 |
|  | Primal, Animist, or Folk religion | 1.49 | 1.00 |

***Table S16a. Nationally representative descriptive statistics for Spain***

| **Characteristic** | **N = 6,290**^1^ |
| --- | --- |
| **Relationship with mother** |  |
| Very good | 4,557 (72%) |
| Somewhat good | 1,258 (20%) |
| Somewhat bad | 248 (3.9%) |
| Very bad | 92 (1.5%) |
| Does not apply | 107 (1.7%) |
| Missing | 28 (0.4%) |
| **Relationship with father** |  |
| Very good | 4,131 (66%) |
| Somewhat good | 1,397 (22%) |
| Somewhat bad | 309 (4.9%) |
| Very bad | 178 (2.8%) |
| Does not apply | 243 (3.9%) |
| Missing | 33 (0.5%) |
| **Parent marital status** |  |
| Yes, married | 5,285 (84%) |
| No, divorced | 378 (6.0%) |
| Never married | 312 (5.0%) |
| No, one or both of them had died | 126 (2.0%) |
| Missing | 188 (3.0%) |
| **Subjective financial status of family growing up** |  |
| Lived comfortably | 2,041 (32%) |
| Got by | 2,956 (47%) |
| Found it difficult | 1,154 (18%) |
| Found it very difficult | 110 (1.7%) |
| Missing | 29 (0.5%) |
| **Abuse** |  |
| Yes | 659 (10%) |
| No | 5,510 (88%) |
| Missing | 122 (1.9%) |
| **Outsider growing up** |  |
| Yes | 579 (9.2%) |
| No | 5,637 (90%) |
| Missing | 75 (1.2%) |
| **Self-rated health growing up** |  |
| Excellent | 2,450 (39%) |
| Very good | 2,286 (36%) |
| Good | 1,235 (20%) |
| Fair | 164 (2.6%) |
| Poor | 135 (2.1%) |
| Missing | 20 (0.3%) |
| **Immigration status** |  |
| Born in this country | 5,479 (87%) |
| Born in another country | 788 (13%) |
| Missing | 23 (0.4%) |
| **Age 12 religious service attendance** |  |
| At least 1/week | 2,391 (38%) |
| 1-3/month | 1,132 (18%) |
| <1/month | 1,287 (20%) |
| Never | 1,445 (23%) |
| Missing | 36 (0.6%) |
| **Age group** |  |
| 1998-2005; current age: 18-24 | 594 (9.4%) |
| 1993-1998; age: 25-29 | 450 (7.2%) |
| 1983-1993; age: 30-39 | 1,111 (18%) |
| 1973-1983; age 40-49 | 1,396 (22%) |
| 1963-1973; age 50-59 | 1,252 (20%) |
| 1953-1963; age 60-69 | 977 (16%) |
| 1943 or earlier; age 80+ | 43 (0.7%) |
| 1943-1953; age 70-79 | 467 (7.4%) |
| Missing | 0 (0%) |
| **Gender** |  |
| Male | 3,142 (50%) |
| Female | 3,119 (50%) |
| Other | 6 (0.1%) |
| Missing | 22 (0.4%) |
| **Religious affiliation at age 12** |  |
| Buddhism | 8 (0.1%) |
| Christianity | 5,119 (81%) |
| Confucianism | 1 (<0.1%) |
| Hinduism | 5 (<0.1%) |
| Islam | 132 (2.1%) |
| Judaism | 5 (<0.1%) |
| No religion/Atheist/Agnostic | 972 (15%) |
| Primal, Animist, or Folk religion | 4 (<0.1%) |
| Sikhism | 2 (<0.1%) |
| Some other religion | 13 (0.2%) |
| Missing | 29 (0.5%) |
| **Race and ethnicity** |  |
| Missing | 6,290 (100%) |
| ^1^n (%) | |

***Table S16b. Regression of self-rated hope on childhood predictors for Spain***

| Variable | Category | Estimate | SE | 95% CI | Global p-value |
| --- | --- | --- | --- | --- | --- |
| Relationship with mother (Ref: Very bad/Somewhat bad) | Very good/Somewhat good | 0.46 | 0.14 | (0.20, 0.73) | <.001 |
| Relationship with father (Ref: Very bad/Somewhat bad) | Very good/Somewhat good | 0.07 | 0.11 | (-0.15, 0.28) | 0.255 |
| Parent marital status (Ref: Parents married) | No, divorced | 0.00 | 0.12 | (-0.24, 0.24) | 0.205 |
|  | Never | -0.03 | 0.13 | (-0.28, 0.22) |  |
|  | No, one or both of them had died | -0.39 | 0.20 | (-0.78, 0.00) |  |
| Subjective financial status of family growing up (Ref: Got by) | Lived comfortably | 0.07 | 0.07 | (-0.06, 0.20) | 0.943 |
|  | Found it difficult | 0.02 | 0.10 | (-0.17, 0.21) |  |
|  | Found it very difficult | -0.13 | 0.27 | (-0.67, 0.40) |  |
| Abuse (Ref: No) | Yes | -0.24 | 0.11 | (-0.45, -0.04) | <.001 |
| Outsider growing up (Ref: No) | Yes | -0.17 | 0.10 | (-0.37, 0.03) | 0.007 |
| Self-rated health growing up (Ref: Good) | Excellent | 0.56 | 0.10 | (0.37, 0.75) | <.001 |
|  | Very good | 0.31 | 0.09 | (0.14, 0.48) |  |
|  | Fair | 0.38 | 0.20 | (-0.01, 0.77) |  |
|  | Poor | 0.66 | 0.26 | (0.15, 1.17) |  |
| Immigration status (Ref: Born in this country) | Born in another country | 0.83 | 0.08 | (0.68, 0.97) | <.001 |
| Age 12 religious service attendance (Ref: Never) | At least 1/week | 0.20 | 0.08 | (0.03, 0.36) | <.001 |
|  | 1-3/month | 0.07 | 0.09 | (-0.11, 0.24) |  |
|  | <1/month | -0.05 | 0.09 | (-0.23, 0.13) |  |
| Age group (Ref: 1998-2005; current age: 18-24) | 1993-1998; age: 25-29 | -0.08 | 0.14 | (-0.36, 0.20) | 0.125 |
|  | 1983-1993; age: 30-39 | 0.03 | 0.12 | (-0.20, 0.27) |  |
|  | 1973-1983; age 40-49 | 0.11 | 0.12 | (-0.12, 0.33) |  |
|  | 1963-1973; age 50-59 | 0.19 | 0.12 | (-0.05, 0.43) |  |
|  | 1953-1963; age 60-69 | 0.22 | 0.13 | (-0.05, 0.48) |  |
|  | 1943 or earlier; age 80+ | 0.24 | 0.33 | (-0.41, 0.89) |  |
|  | 1943-1953; age 70-79 | -0.06 | 0.19 | (-0.42, 0.31) |  |
| Gender (Ref: Male) | Female | -0.06 | 0.06 | (-0.18, 0.06) | 0.048 |
|  | Other | 0.38 | 0.25 | (-0.11, 0.87) |  |
| Religious affiliation at age 12 (Ref: No religion/Atheist/Agnostic) | Christianity | 0.10 | 0.09 | (-0.07, 0.28) | 0.189 |
|  | Some other religion | -0.19 | 0.24 | (-0.67, 0.28) |  |

***Table S16c. Sensitivity to unmeasured confounding of childhood predictors in Spain***

| Variable | Category | E-value for Estimate | E-value for 95% CI |
| --- | --- | --- | --- |
| Relationship with mother (Ref: Very bad/Somewhat bad)) | Very good/Somewhat good | 1.80 | 1.42 |
| Relationship with father (Ref: Very bad/Somewhat bad)) | Very good/Somewhat good | 1.21 | 1.00 |
| Parent marital status (Ref: Parents married) | No, divorced | 1.00 | 1.00 |
|  | Never | 1.14 | 1.00 |
|  | No, one or both of them had died | 1.69 | 1.00 |
| Subjective financial status of family growing up (Ref: Got by) | Lived comfortably | 1.23 | 1.00 |
|  | Found it difficult | 1.11 | 1.00 |
|  | Found it very difficult | 1.33 | 1.00 |
| Abuse (Ref: No) | Yes | 1.49 | 1.15 |
| Outsider growing up (Ref: No) | Yes | 1.39 | 1.00 |
| Self-rated health growing up (Ref: Good) | Excellent | 1.93 | 1.67 |
|  | Very good | 1.59 | 1.34 |
|  | Fair | 1.68 | 1.00 |
|  | Poor | 2.08 | 1.36 |
| Immigration status (Ref: Born in this country) | Born in another country | 2.32 | 2.10 |
| Age 12 religious service attendance (Ref: Never) | At least 1/week | 1.43 | 1.15 |
|  | 1-3/month | 1.21 | 1.00 |
|  | <1/month | 1.18 | 1.00 |
| Age group (Ref: 1998-2005; current age: 18-24) | 1993-1998; age: 25-29 | 1.24 | 1.00 |
|  | 1983-1993; age: 30-39 | 1.15 | 1.00 |
|  | 1973-1983; age 40-49 | 1.29 | 1.00 |
|  | 1963-1973; age 50-59 | 1.41 | 1.00 |
|  | 1953-1963; age 60-69 | 1.45 | 1.00 |
|  | 1943 or earlier; age 80+ | 1.48 | 1.00 |
|  | 1943-1953; age 70-79 | 1.20 | 1.00 |
| Gender (Ref: Male) | Female | 1.20 | 1.00 |
|  | Other | 1.68 | 1.00 |
| Religious affiliation at age 12 (Ref: No religion/Atheist/Agnostic) | Christianity | 1.28 | 1.00 |
|  | Some other religion | 1.42 | 1.00 |

***Table S17a. Nationally representative descriptive statistics for Tanzania***

| **Characteristic** | **N = 9,075**^1^ |
| --- | --- |
| **Relationship with mother** |  |
| Very good | 7,739 (85%) |
| Somewhat good | 796 (8.8%) |
| Somewhat bad | 84 (0.9%) |
| Very bad | 84 (0.9%) |
| Does not apply | 303 (3.3%) |
| Missing | 70 (0.8%) |
| **Relationship with father** |  |
| Very good | 6,831 (75%) |
| Somewhat good | 1,101 (12%) |
| Somewhat bad | 203 (2.2%) |
| Very bad | 247 (2.7%) |
| Does not apply | 550 (6.1%) |
| Missing | 142 (1.6%) |
| **Parent marital status** |  |
| Yes, married | 6,929 (76%) |
| No, divorced | 678 (7.5%) |
| Never married | 751 (8.3%) |
| No, one or both of them had died | 313 (3.4%) |
| Missing | 404 (4.4%) |
| **Subjective financial status of family growing up** |  |
| Lived comfortably | 2,611 (29%) |
| Got by | 2,909 (32%) |
| Found it difficult | 2,679 (30%) |
| Found it very difficult | 814 (9.0%) |
| Missing | 61 (0.7%) |
| **Abuse** |  |
| Yes | 716 (7.9%) |
| No | 8,328 (92%) |
| Missing | 32 (0.3%) |
| **Outsider growing up** |  |
| Yes | 734 (8.1%) |
| No | 8,320 (92%) |
| Missing | 22 (0.2%) |
| **Self-rated health growing up** |  |
| Excellent | 2,406 (27%) |
| Very good | 2,036 (22%) |
| Good | 2,946 (32%) |
| Fair | 1,177 (13%) |
| Poor | 456 (5.0%) |
| Missing | 54 (0.6%) |
| **Immigration status** |  |
| Born in this country | 9,048 (100%) |
| Born in another country | 25 (0.3%) |
| Missing | 1 (<0.1%) |
| **Age 12 religious service attendance** |  |
| At least 1/week | 5,580 (61%) |
| 1-3/month | 2,383 (26%) |
| <1/month | 333 (3.7%) |
| Never | 595 (6.6%) |
| Missing | 184 (2.0%) |
| **Age group** |  |
| 1998-2005; current age: 18-24 | 2,284 (25%) |
| 1993-1998; age: 25-29 | 1,349 (15%) |
| 1983-1993; age: 30-39 | 2,060 (23%) |
| 1973-1983; age 40-49 | 1,503 (17%) |
| 1963-1973; age 50-59 | 912 (10%) |
| 1953-1963; age 60-69 | 575 (6.3%) |
| 1943 or earlier; age 80+ | 93 (1.0%) |
| 1943-1953; age 70-79 | 297 (3.3%) |
| Missing | 2 (<0.1%) |
| **Gender** |  |
| Male | 4,299 (47%) |
| Female | 4,776 (53%) |
| Other | 0 (0%) |
| Missing | 0 (0%) |
| **Religious affiliation at age 12** |  |
| Baha’i | 1 (<0.1%) |
| Christianity | 5,651 (62%) |
| Islam | 3,060 (34%) |
| No religion/Atheist/Agnostic | 345 (3.8%) |
| Primal, Animist, or Folk religion | 11 (0.1%) |
| Missing | 7 (<0.1%) |
| **Race and ethnicity** |  |
| African | 9,060 (100%) |
| Arab | 11 (0.1%) |
| Indian | 3 (<0.1%) |
| Missing | 2 (<0.1%) |
| ^1^n (%) | |

***Table S17b. Regression of self-rated hope on childhood predictors for Tanzania***

| Variable | Category | Estimate | SE | 95% CI | Global p-value |
| --- | --- | --- | --- | --- | --- |
| Relationship with mother (Ref: Very bad/Somewhat bad) | Very good/Somewhat good | -0.20 | 0.21 | (-0.61, 0.21) | 0.016 |
| Relationship with father (Ref: Very bad/Somewhat bad) | Very good/Somewhat good | -0.07 | 0.16 | (-0.39, 0.25) | 0.557 |
| Parent marital status (Ref: Parents married) | No, divorced | -0.16 | 0.15 | (-0.46, 0.13) | 0.027 |
|  | Never | 0.12 | 0.11 | (-0.10, 0.35) |  |
|  | No, one or both of them had died | -0.35 | 0.22 | (-0.78, 0.09) |  |
| Subjective financial status of family growing up (Ref: Got by) | Lived comfortably | -0.03 | 0.09 | (-0.21, 0.15) | 0.675 |
|  | Found it difficult | -0.03 | 0.08 | (-0.20, 0.13) |  |
|  | Found it very difficult | -0.27 | 0.17 | (-0.61, 0.07) |  |
| Abuse (Ref: No) | Yes | -0.40 | 0.15 | (-0.70, -0.10) | <.001 |
| Outsider growing up (Ref: No) | Yes | -0.23 | 0.14 | (-0.51, 0.05) | 0.012 |
| Self-rated health growing up (Ref: Good) | Excellent | 0.20 | 0.10 | (0.01, 0.38) | 0.001 |
|  | Very good | 0.22 | 0.10 | (0.02, 0.43) |  |
|  | Fair | 0.19 | 0.11 | (-0.03, 0.41) |  |
|  | Poor | -0.19 | 0.20 | (-0.59, 0.21) |  |
| Immigration status (Ref: Born in this country) | Born in another country | -0.14 | 0.62 | (-1.37, 1.08) | 0.996 |
| Age 12 religious service attendance (Ref: Never) | At least 1/week | 0.28 | 0.18 | (-0.07, 0.63) | <.001 |
|  | 1-3/month | -0.05 | 0.20 | (-0.44, 0.34) |  |
|  | <1/month | -0.28 | 0.29 | (-0.85, 0.29) |  |
| Age group (Ref: 1998-2005; current age: 18-24) | 1993-1998; age: 25-29 | 0.19 | 0.10 | (0.00, 0.39) | <.001 |
|  | 1983-1993; age: 30-39 | -0.02 | 0.10 | (-0.22, 0.17) |  |
|  | 1973-1983; age 40-49 | -0.24 | 0.11 | (-0.45, -0.02) |  |
|  | 1963-1973; age 50-59 | -0.38 | 0.14 | (-0.66, -0.10) |  |
|  | 1953-1963; age 60-69 | -0.35 | 0.18 | (-0.71, 0.00) |  |
|  | 1943 or earlier; age 80+ | -1.42 | 0.56 | (-2.51, -0.33) |  |
|  | 1943-1953; age 70-79 | -0.75 | 0.31 | (-1.36, -0.15) |  |
| Gender (Ref: Male) | Female | -0.05 | 0.07 | (-0.19, 0.10) | 0.768 |
| Religious affiliation at age 12 (Ref: No religion/Atheist/Agnostic) | Christianity | 0.69 | 0.34 | (0.03, 1.35) | <.001 |
|  | Islam | 0.52 | 0.34 | (-0.15, 1.20) |  |
|  | Some other religion | 1.83 | 0.36 | (1.13, 2.54) |  |
| Race and ethnicity plurality (Ref: Majority) | Minority | -1.59 | 0.95 | (-3.46, 0.28) | 0.009 |

***Table S17c. Sensitivity to unmeasured confounding of childhood predictors in Tanzania***

| Variable | Category | E-value for Estimate | E-value for 95% CI |
| --- | --- | --- | --- |
| Relationship with mother (Ref: Very bad/Somewhat bad)) | Very good/Somewhat good | 1.35 | 1.00 |
| Relationship with father (Ref: Very bad/Somewhat bad)) | Very good/Somewhat good | 1.18 | 1.00 |
| Parent marital status (Ref: Parents married) | No, divorced | 1.31 | 1.00 |
|  | Never | 1.26 | 1.00 |
|  | No, one or both of them had died | 1.52 | 1.00 |
| Subjective financial status of family growing up (Ref: Got by) | Lived comfortably | 1.11 | 1.00 |
|  | Found it difficult | 1.12 | 1.00 |
|  | Found it very difficult | 1.43 | 1.00 |
| Abuse (Ref: No) | Yes | 1.57 | 1.24 |
| Outsider growing up (Ref: No) | Yes | 1.39 | 1.00 |
| Self-rated health growing up (Ref: Good) | Excellent | 1.35 | 1.07 |
|  | Very good | 1.38 | 1.09 |
|  | Fair | 1.34 | 1.00 |
|  | Poor | 1.35 | 1.00 |
| Immigration status (Ref: Born in this country) | Born in another country | 1.29 | 1.00 |
| Age 12 religious service attendance (Ref: Never) | At least 1/week | 1.44 | 1.00 |
|  | 1-3/month | 1.15 | 1.00 |
|  | <1/month | 1.44 | 1.00 |
| Age group (Ref: 1998-2005; current age: 18-24) | 1993-1998; age: 25-29 | 1.35 | 1.00 |
|  | 1983-1993; age: 30-39 | 1.10 | 1.00 |
|  | 1973-1983; age 40-49 | 1.40 | 1.09 |
|  | 1963-1973; age 50-59 | 1.55 | 1.23 |
|  | 1953-1963; age 60-69 | 1.52 | 1.03 |
|  | 1943 or earlier; age 80+ | 2.69 | 1.50 |
|  | 1943-1953; age 70-79 | 1.94 | 1.30 |
| Gender (Ref: Male) | Female | 1.15 | 1.00 |
| Religious affiliation at age 12 (Ref: No religion/Atheist/Agnostic) | Christianity | 1.87 | 1.12 |
|  | Some other religion | 3.24 | 2.35 |
| Race and ethnicity plurality (Ref: Majority) | Minority | 2.91 | 1.00 |
|  | Islam | 1.70 | 1.00 |

***Table S18a. Nationally representative descriptive statistics for Turkey***

| **Characteristic** | **N = 1,473**^1^ |
| --- | --- |
| **Relationship with mother** |  |
| Very good | 970 (66%) |
| Somewhat good | 401 (27%) |
| Somewhat bad | 48 (3.2%) |
| Very bad | 26 (1.8%) |
| Does not apply | 21 (1.4%) |
| Missing | 7 (0.5%) |
| **Relationship with father** |  |
| Very good | 795 (54%) |
| Somewhat good | 425 (29%) |
| Somewhat bad | 73 (5.0%) |
| Very bad | 95 (6.5%) |
| Does not apply | 60 (4.1%) |
| Missing | 25 (1.7%) |
| **Parent marital status** |  |
| Yes, married | 1,325 (90%) |
| No, divorced | 57 (3.9%) |
| Never married | 7 (0.5%) |
| No, one or both of them had died | 61 (4.1%) |
| Missing | 23 (1.5%) |
| **Subjective financial status of family growing up** |  |
| Lived comfortably | 498 (34%) |
| Got by | 647 (44%) |
| Found it difficult | 218 (15%) |
| Found it very difficult | 108 (7.3%) |
| Missing | 2 (0.1%) |
| **Abuse** |  |
| Yes | 158 (11%) |
| No | 1,290 (88%) |
| Missing | 25 (1.7%) |
| **Outsider growing up** |  |
| Yes | 157 (11%) |
| No | 1,306 (89%) |
| Missing | 9 (0.6%) |
| **Self-rated health growing up** |  |
| Excellent | 377 (26%) |
| Very good | 410 (28%) |
| Good | 419 (28%) |
| Fair | 220 (15%) |
| Poor | 47 (3.2%) |
| Missing | 0 (<0.1%) |
| **Immigration status** |  |
| Born in this country | 1,415 (96%) |
| Born in another country | 58 (4.0%) |
| Missing | 0 (0%) |
| **Age 12 religious service attendance** |  |
| At least 1/week | 609 (41%) |
| 1-3/month | 238 (16%) |
| <1/month | 225 (15%) |
| Never | 383 (26%) |
| Missing | 18 (1.2%) |
| **Age group** |  |
| 1998-2005; current age: 18-24 | 222 (15%) |
| 1993-1998; age: 25-29 | 152 (10%) |
| 1983-1993; age: 30-39 | 315 (21%) |
| 1973-1983; age 40-49 | 312 (21%) |
| 1963-1973; age 50-59 | 225 (15%) |
| 1953-1963; age 60-69 | 164 (11%) |
| 1943 or earlier; age 80+ | 18 (1.2%) |
| 1943-1953; age 70-79 | 65 (4.4%) |
| Missing | 0 (0%) |
| **Gender** |  |
| Male | 754 (51%) |
| Female | 719 (49%) |
| Other | 0 (0%) |
| Missing | 0 (0%) |
| **Religious affiliation at age 12** |  |
| Christianity | 1 (<0.1%) |
| Islam | 1,439 (98%) |
| Judaism | 1 (<0.1%) |
| No religion/Atheist/Agnostic | 13 (0.9%) |
| Missing | 19 (1.3%) |
| **Race and ethnicity** |  |
| Albanian | 8 (0.5%) |
| Arab | 51 (3.5%) |
| Armenian | 1 (<0.1%) |
| Azeri | 9 (0.6%) |
| Bosnian | 5 (0.3%) |
| Circassian | 19 (1.3%) |
| Georgian | 4 (0.3%) |
| Greek | 1 (<0.1%) |
| Kurdish/Zaza | 252 (17%) |
| Laz | 25 (1.7%) |
| Other | 58 (3.9%) |
| Turkish | 1,030 (70%) |
| Uyghur | 1 (<0.1%) |
| Missing | 9 (0.6%) |
| ^1^n (%) | |

***Table S18b. Regression of self-rated hope on childhood predictors for Turkey***

| Variable | Category | Estimate | SE | 95% CI | Global p-value |
| --- | --- | --- | --- | --- | --- |
| Relationship with mother (Ref: Very bad/Somewhat bad) | Very good/Somewhat good | -0.09 | 0.38 | (-0.82, 0.65) | 0.853 |
| Relationship with father (Ref: Very bad/Somewhat bad) | Very good/Somewhat good | 0.09 | 0.27 | (-0.44, 0.62) | 0.759 |
| Parent marital status (Ref: Parents married) | No, divorced | 1.00 | 0.35 | (0.32, 1.69) | <.001 |
|  | Never | -0.02 | 0.90 | (-1.80, 1.75) |  |
|  | No, one or both of them had died | -0.06 | 0.52 | (-1.07, 0.95) |  |
| Subjective financial status of family growing up (Ref: Got by) | Lived comfortably | 0.51 | 0.18 | (0.15, 0.87) | <.001 |
|  | Found it difficult | -0.28 | 0.30 | (-0.88, 0.32) |  |
|  | Found it very difficult | -0.23 | 0.45 | (-1.11, 0.66) |  |
| Abuse (Ref: No) | Yes | -0.10 | 0.25 | (-0.59, 0.39) | 0.959 |
| Outsider growing up (Ref: No) | Yes | -0.69 | 0.27 | (-1.22, -0.16) | <.001 |
| Self-rated health growing up (Ref: Good) | Excellent | 0.27 | 0.23 | (-0.18, 0.71) | 0.414 |
|  | Very good | 0.07 | 0.22 | (-0.36, 0.50) |  |
|  | Fair | -0.13 | 0.28 | (-0.68, 0.42) |  |
|  | Poor | -0.75 | 0.56 | (-1.84, 0.35) |  |
| Immigration status (Ref: Born in this country) | Born in another country | 0.93 | 0.32 | (0.30, 1.56) | <.001 |
| Age 12 religious service attendance (Ref: Never) | At least 1/week | 0.69 | 0.23 | (0.24, 1.14) | <.001 |
|  | 1-3/month | 0.27 | 0.27 | (-0.27, 0.81) |  |
|  | <1/month | -0.20 | 0.29 | (-0.76, 0.36) |  |
| Age group (Ref: 1998-2005; current age: 18-24) | 1993-1998; age: 25-29 | 0.14 | 0.31 | (-0.47, 0.76) | <.001 |
|  | 1983-1993; age: 30-39 | 0.32 | 0.27 | (-0.20, 0.84) |  |
|  | 1973-1983; age 40-49 | 0.68 | 0.26 | (0.16, 1.19) |  |
|  | 1963-1973; age 50-59 | 1.05 | 0.27 | (0.52, 1.58) |  |
|  | 1953-1963; age 60-69 | 0.47 | 0.35 | (-0.22, 1.16) |  |
|  | 1943 or earlier; age 80+ | 1.73 | 0.62 | (0.52, 2.94) |  |
|  | 1943-1953; age 70-79 | -0.24 | 0.61 | (-1.43, 0.94) |  |
| Gender (Ref: Male) | Female | 0.25 | 0.18 | (-0.10, 0.61) | 0.048 |
| Religious affiliation at age 12 (Ref: Islam) | Some other religion | -0.30 | 0.67 | (-1.63, 1.03) | 0.909 |
| Race and ethnicity plurality (Ref: Majority) | Minority | -0.03 | 0.20 | (-0.43, 0.37) | 1 |

***Table S18c. Sensitivity to unmeasured confounding of childhood predictors in Turkey***

| Variable | Category | E-value for Estimate | E-value for 95% CI |
| --- | --- | --- | --- |
| Relationship with mother (Ref: Very bad/Somewhat bad)) | Very good/Somewhat good | 1.21 | 1.00 |
| Relationship with father (Ref: Very bad/Somewhat bad)) | Very good/Somewhat good | 1.21 | 1.00 |
| Parent marital status (Ref: Parents married) | No, divorced | 2.20 | 1.49 |
|  | Never | 1.10 | 1.00 |
|  | No, one or both of them had died | 1.17 | 1.00 |
| Subjective financial status of family growing up (Ref: Got by) | Lived comfortably | 1.68 | 1.29 |
|  | Found it difficult | 1.44 | 1.00 |
|  | Found it very difficult | 1.38 | 1.00 |
| Abuse (Ref: No) | Yes | 1.22 | 1.00 |
| Outsider growing up (Ref: No) | Yes | 1.86 | 1.30 |
| Self-rated health growing up (Ref: Good) | Excellent | 1.43 | 1.00 |
|  | Very good | 1.18 | 1.00 |
|  | Fair | 1.26 | 1.00 |
|  | Poor | 1.92 | 1.00 |
| Immigration status (Ref: Born in this country) | Born in another country | 2.11 | 1.46 |
| Age 12 religious service attendance (Ref: Never) | At least 1/week | 1.86 | 1.40 |
|  | 1-3/month | 1.43 | 1.00 |
|  | <1/month | 1.35 | 1.00 |
| Age group (Ref: 1998-2005; current age: 18-24) | 1993-1998; age: 25-29 | 1.29 | 1.00 |
|  | 1983-1993; age: 30-39 | 1.49 | 1.00 |
|  | 1973-1983; age 40-49 | 1.85 | 1.31 |
|  | 1963-1973; age 50-59 | 2.24 | 1.69 |
|  | 1953-1963; age 60-69 | 1.64 | 1.00 |
|  | 1943 or earlier; age 80+ | 3.07 | 1.69 |
|  | 1943-1953; age 70-79 | 1.40 | 1.00 |
| Gender (Ref: Male) | Female | 1.41 | 1.00 |
| Religious affiliation at age 12 (Ref: Islam) | Some other religion | 1.46 | 1.00 |
| Race and ethnicity plurality (Ref: Majority) | Minority | 1.11 | 1.00 |

***Table S19a. Nationally representative descriptive statistics for United Kingdom***

| **Characteristic** | **N = 5,368**^1^ |
| --- | --- |
| **Relationship with mother** |  |
| Very good | 3,435 (64%) |
| Somewhat good | 1,338 (25%) |
| Somewhat bad | 325 (6.1%) |
| Very bad | 150 (2.8%) |
| Does not apply | 92 (1.7%) |
| Missing | 27 (0.5%) |
| **Relationship with father** |  |
| Very good | 2,907 (54%) |
| Somewhat good | 1,383 (26%) |
| Somewhat bad | 407 (7.6%) |
| Very bad | 321 (6.0%) |
| Does not apply | 321 (6.0%) |
| Missing | 29 (0.5%) |
| **Parent marital status** |  |
| Yes, married | 4,343 (81%) |
| No, divorced | 481 (9.0%) |
| Never married | 315 (5.9%) |
| No, one or both of them had died | 154 (2.9%) |
| Missing | 75 (1.4%) |
| **Subjective financial status of family growing up** |  |
| Lived comfortably | 2,552 (48%) |
| Got by | 1,933 (36%) |
| Found it difficult | 632 (12%) |
| Found it very difficult | 230 (4.3%) |
| Missing | 22 (0.4%) |
| **Abuse** |  |
| Yes | 864 (16%) |
| No | 4,455 (83%) |
| Missing | 49 (0.9%) |
| **Outsider growing up** |  |
| Yes | 1,017 (19%) |
| No | 4,308 (80%) |
| Missing | 43 (0.8%) |
| **Self-rated health growing up** |  |
| Excellent | 2,154 (40%) |
| Very good | 1,736 (32%) |
| Good | 995 (19%) |
| Fair | 332 (6.2%) |
| Poor | 130 (2.4%) |
| Missing | 20 (0.4%) |
| **Immigration status** |  |
| Born in this country | 4,659 (87%) |
| Born in another country | 682 (13%) |
| Missing | 27 (0.5%) |
| **Age 12 religious service attendance** |  |
| At least 1/week | 1,732 (32%) |
| 1-3/month | 733 (14%) |
| <1/month | 903 (17%) |
| Never | 1,972 (37%) |
| Missing | 28 (0.5%) |
| **Age group** |  |
| 1998-2005; current age: 18-24 | 490 (9.1%) |
| 1993-1998; age: 25-29 | 391 (7.3%) |
| 1983-1993; age: 30-39 | 946 (18%) |
| 1973-1983; age 40-49 | 827 (15%) |
| 1963-1973; age 50-59 | 949 (18%) |
| 1953-1963; age 60-69 | 889 (17%) |
| 1943 or earlier; age 80+ | 163 (3.0%) |
| 1943-1953; age 70-79 | 711 (13%) |
| Missing | 1 (<0.1%) |
| **Gender** |  |
| Male | 2,557 (48%) |
| Female | 2,789 (52%) |
| Other | 14 (0.3%) |
| Missing | 9 (0.2%) |
| **Religious affiliation at age 12** |  |
| Baha’i | 5 (<0.1%) |
| Buddhism | 15 (0.3%) |
| Christianity | 3,461 (64%) |
| Confucianism | 3 (<0.1%) |
| Hinduism | 88 (1.6%) |
| Islam | 230 (4.3%) |
| Jainism | 0 (<0.1%) |
| Judaism | 59 (1.1%) |
| No religion/Atheist/Agnostic | 1,409 (26%) |
| Primal, Animist, or Folk religion | 22 (0.4%) |
| Sikhism | 30 (0.6%) |
| Some other religion | 24 (0.5%) |
| Taoism | 2 (<0.1%) |
| Missing | 21 (0.4%) |
| **Race and ethnicity** |  |
| Asian | 426 (7.9%) |
| Black | 152 (2.8%) |
| Other | 96 (1.8%) |
| White | 4,647 (87%) |
| Missing | 47 (0.9%) |
| ^1^n (%) | |

***Table S19b. Regression of self-rated hope on childhood predictors for United Kingdom***

| Variable | Category | Estimate | SE | 95% CI | Global p-value |
| --- | --- | --- | --- | --- | --- |
| Relationship with mother (Ref: Very bad/Somewhat bad) | Very good/Somewhat good | 0.28 | 0.17 | (-0.06, 0.62) | <.001 |
| Relationship with father (Ref: Very bad/Somewhat bad) | Very good/Somewhat good | 0.16 | 0.13 | (-0.10, 0.42) | 0.001 |
| Parent marital status (Ref: Parents married) | No, divorced | 0.01 | 0.16 | (-0.31, 0.33) | 0.998 |
|  | Never | -0.01 | 0.22 | (-0.44, 0.42) |  |
|  | No, one or both of them had died | -0.27 | 0.35 | (-0.95, 0.42) |  |
| Subjective financial status of family growing up (Ref: Got by) | Lived comfortably | 0.00 | 0.09 | (-0.18, 0.18) | 0.058 |
|  | Found it difficult | 0.14 | 0.14 | (-0.12, 0.41) |  |
|  | Found it very difficult | -0.51 | 0.29 | (-1.09, 0.06) |  |
| Abuse (Ref: No) | Yes | -0.32 | 0.13 | (-0.56, -0.07) | <.001 |
| Outsider growing up (Ref: No) | Yes | -0.11 | 0.12 | (-0.34, 0.12) | 0.352 |
| Self-rated health growing up (Ref: Good) | Excellent | 0.63 | 0.12 | (0.39, 0.87) | <.001 |
|  | Very good | 0.51 | 0.12 | (0.28, 0.75) |  |
|  | Fair | -0.57 | 0.22 | (-1.00, -0.15) |  |
|  | Poor | -0.46 | 0.41 | (-1.27, 0.35) |  |
| Immigration status (Ref: Born in this country) | Born in another country | 0.20 | 0.13 | (-0.06, 0.45) | 0.024 |
| Age 12 religious service attendance (Ref: Never) | At least 1/week | 0.18 | 0.12 | (-0.07, 0.42) | <.001 |
|  | 1-3/month | 0.36 | 0.13 | (0.10, 0.63) |  |
|  | <1/month | 0.06 | 0.13 | (-0.19, 0.31) |  |
| Age group (Ref: 1998-2005; current age: 18-24) | 1993-1998; age: 25-29 | 0.43 | 0.21 | (0.02, 0.84) | 0.473 |
|  | 1983-1993; age: 30-39 | 0.13 | 0.19 | (-0.25, 0.50) |  |
|  | 1973-1983; age 40-49 | 0.17 | 0.19 | (-0.20, 0.55) |  |
|  | 1963-1973; age 50-59 | 0.06 | 0.19 | (-0.32, 0.43) |  |
|  | 1953-1963; age 60-69 | 0.12 | 0.21 | (-0.28, 0.52) |  |
|  | 1943 or earlier; age 80+ | 0.29 | 0.32 | (-0.34, 0.93) |  |
|  | 1943-1953; age 70-79 | 0.23 | 0.22 | (-0.19, 0.66) |  |
| Gender (Ref: Male) | Female | -0.03 | 0.08 | (-0.20, 0.13) | 0.024 |
|  | Other | -1.41 | 0.71 | (-2.79, -0.03) |  |
| Religious affiliation at age 12 (Ref: No religion/Atheist/Agnostic) | Christianity | 0.58 | 0.12 | (0.34, 0.81) | <.001 |
|  | Islam | 0.49 | 0.22 | (0.05, 0.93) |  |
|  | Some other religion | 0.12 | 0.22 | (-0.32, 0.55) |  |
| Race and ethnicity plurality (Ref: Majority) | Minority | 0.46 | 0.15 | (0.15, 0.76) | <.001 |

***Table S19c. Sensitivity to unmeasured confounding of childhood predictors in United Kingdom***

| Variable | Category | E-value for Estimate | E-value for 95% CI |
| --- | --- | --- | --- |
| Relationship with mother (Ref: Very bad/Somewhat bad)) | Very good/Somewhat good | 1.49 | 1.00 |
| Relationship with father (Ref: Very bad/Somewhat bad)) | Very good/Somewhat good | 1.34 | 1.00 |
| Parent marital status (Ref: Parents married) | No, divorced | 1.08 | 1.00 |
|  | Never | 1.07 | 1.00 |
|  | No, one or both of them had died | 1.48 | 1.00 |
| Subjective financial status of family growing up (Ref: Got by) | Lived comfortably | 1.04 | 1.00 |
|  | Found it difficult | 1.31 | 1.00 |
|  | Found it very difficult | 1.77 | 1.00 |
| Abuse (Ref: No) | Yes | 1.54 | 1.21 |
| Outsider growing up (Ref: No) | Yes | 1.27 | 1.00 |
| Self-rated health growing up (Ref: Good) | Excellent | 1.92 | 1.63 |
|  | Very good | 1.77 | 1.49 |
|  | Fair | 1.85 | 1.33 |
|  | Poor | 1.72 | 1.00 |
| Immigration status (Ref: Born in this country) | Born in another country | 1.39 | 1.00 |
| Age 12 religious service attendance (Ref: Never) | At least 1/week | 1.36 | 1.00 |
|  | 1-3/month | 1.59 | 1.25 |
|  | <1/month | 1.19 | 1.00 |
| Age group (Ref: 1998-2005; current age: 18-24) | 1993-1998; age: 25-29 | 1.67 | 1.10 |
|  | 1983-1993; age: 30-39 | 1.29 | 1.00 |
|  | 1973-1983; age 40-49 | 1.36 | 1.00 |
|  | 1963-1973; age 50-59 | 1.18 | 1.00 |
|  | 1953-1963; age 60-69 | 1.28 | 1.00 |
|  | 1943 or earlier; age 80+ | 1.51 | 1.00 |
|  | 1943-1953; age 70-79 | 1.43 | 1.00 |
| Gender (Ref: Male) | Female | 1.14 | 1.00 |
|  | Other | 2.98 | 1.13 |
| Religious affiliation at age 12 (Ref: No religion/Atheist/Agnostic) | Christianity | 1.85 | 1.57 |
|  | Some other religion | 1.28 | 1.00 |
| Race and ethnicity plurality (Ref: Majority) | Minority | 1.71 | 1.33 |
|  | Islam | 1.75 | 1.17 |

***Table S20a. Nationally representative descriptive statistics for United States***

| **Characteristic** | **N = 38,312**^1^ |
| --- | --- |
| **Relationship with mother** |  |
| Very good | 20,590 (54%) |
| Somewhat good | 11,525 (30%) |
| Somewhat bad | 3,523 (9.2%) |
| Very bad | 1,874 (4.9%) |
| Does not apply | 694 (1.8%) |
| Missing | 106 (0.3%) |
| **Relationship with father** |  |
| Very good | 15,313 (40%) |
| Somewhat good | 12,665 (33%) |
| Somewhat bad | 4,879 (13%) |
| Very bad | 2,604 (6.8%) |
| Does not apply | 2,811 (7.3%) |
| Missing | 38 (0.1%) |
| **Parent marital status** |  |
| Yes, married | 27,415 (72%) |
| No, divorced | 6,325 (17%) |
| Never married | 3,048 (8.0%) |
| No, one or both of them had died | 1,024 (2.7%) |
| Missing | 500 (1.3%) |
| **Subjective financial status of family growing up** |  |
| Lived comfortably | 15,116 (39%) |
| Got by | 15,682 (41%) |
| Found it difficult | 5,152 (13%) |
| Found it very difficult | 2,342 (6.1%) |
| Missing | 19 (<0.1%) |
| **Abuse** |  |
| Yes | 10,026 (26%) |
| No | 28,045 (73%) |
| Missing | 242 (0.6%) |
| **Outsider growing up** |  |
| Yes | 10,185 (27%) |
| No | 27,714 (72%) |
| Missing | 413 (1.1%) |
| **Self-rated health growing up** |  |
| Excellent | 16,866 (44%) |
| Very good | 12,108 (32%) |
| Good | 6,444 (17%) |
| Fair | 2,303 (6.0%) |
| Poor | 520 (1.4%) |
| Missing | 71 (0.2%) |
| **Immigration status** |  |
| Born in this country | 34,865 (91%) |
| Born in another country | 3,020 (7.9%) |
| Missing | 427 (1.1%) |
| **Age 12 religious service attendance** |  |
| At least 1/week | 18,609 (49%) |
| 1-3/month | 6,644 (17%) |
| <1/month | 5,829 (15%) |
| Never | 7,085 (18%) |
| Missing | 145 (0.4%) |
| **Age group** |  |
| 1998-2005; current age: 18-24 | 2,682 (7.0%) |
| 1993-1998; age: 25-29 | 3,540 (9.2%) |
| 1983-1993; age: 30-39 | 7,284 (19%) |
| 1973-1983; age 40-49 | 5,649 (15%) |
| 1963-1973; age 50-59 | 6,745 (18%) |
| 1953-1963; age 60-69 | 6,832 (18%) |
| 1943 or earlier; age 80+ | 1,525 (4.0%) |
| 1943-1953; age 70-79 | 4,054 (11%) |
| Missing | 0 (0%) |
| **Gender** |  |
| Male | 18,222 (48%) |
| Female | 19,562 (51%) |
| Other | 392 (1.0%) |
| Missing | 136 (0.4%) |
| **Religious affiliation at age 12** |  |
| Baha’i | 4 (<0.1%) |
| Buddhism | 172 (0.4%) |
| Christianity | 30,444 (79%) |
| Confucianism | 8 (<0.1%) |
| Hinduism | 203 (0.5%) |
| Islam | 220 (0.6%) |
| Jainism | 18 (<0.1%) |
| Judaism | 787 (2.1%) |
| No religion/Atheist/Agnostic | 5,845 (15%) |
| Primal, Animist, or Folk religion | 67 (0.2%) |
| Shinto | 6 (<0.1%) |
| Sikhism | 47 (0.1%) |
| Some other religion | 359 (0.9%) |
| Taoism | 17 (<0.1%) |
| Missing | 115 (0.3%) |
| **Race and ethnicity** |  |
| Asian | 2,466 (6.4%) |
| Black | 4,501 (12%) |
| Hispanic | 6,724 (18%) |
| Other | 997 (2.6%) |
| White | 23,605 (62%) |
| Missing | 20 (<0.1%) |
| ^1^n (%) | |

***Table S20b. Regression of self-rated hope on childhood predictors for United States***

| Variable | Category | Estimate | SE | 95% CI | Global p-value |
| --- | --- | --- | --- | --- | --- |
| Relationship with mother (Ref: Very bad/Somewhat bad) | Very good/Somewhat good | 0.24 | 0.11 | (0.03, 0.45) | <.001 |
| Relationship with father (Ref: Very bad/Somewhat bad) | Very good/Somewhat good | 0.25 | 0.09 | (0.07, 0.42) | <.001 |
| Parent marital status (Ref: Parents married) | No, divorced | 0.07 | 0.09 | (-0.12, 0.25) | 0.364 |
|  | Never | 0.05 | 0.20 | (-0.36, 0.45) |  |
|  | No, one or both of them had died | -0.41 | 0.26 | (-0.92, 0.10) |  |
| Subjective financial status of family growing up (Ref: Got by) | Lived comfortably | 0.11 | 0.06 | (0.00, 0.22) | 0.006 |
|  | Found it difficult | 0.17 | 0.10 | (-0.02, 0.36) |  |
|  | Found it very difficult | 0.23 | 0.15 | (-0.07, 0.53) |  |
| Abuse (Ref: No) | Yes | -0.23 | 0.08 | (-0.38, -0.07) | <.001 |
| Outsider growing up (Ref: No) | Yes | -0.35 | 0.09 | (-0.52, -0.18) | <.001 |
| Self-rated health growing up (Ref: Good) | Excellent | 0.84 | 0.09 | (0.65, 1.02) | <.001 |
|  | Very good | 0.43 | 0.10 | (0.24, 0.62) |  |
|  | Fair | -0.30 | 0.21 | (-0.72, 0.12) |  |
|  | Poor | -0.18 | 0.46 | (-1.08, 0.73) |  |
| Immigration status (Ref: Born in this country) | Born in another country | 0.27 | 0.13 | (0.03, 0.52) | <.001 |
| Age 12 religious service attendance (Ref: Never) | At least 1/week | 0.20 | 0.10 | (0.01, 0.39) | <.001 |
|  | 1-3/month | 0.08 | 0.11 | (-0.13, 0.28) |  |
|  | <1/month | 0.00 | 0.11 | (-0.22, 0.22) |  |
| Age group (Ref: 1998-2005; current age: 18-24) | 1993-1998; age: 25-29 | 0.09 | 0.24 | (-0.37, 0.56) | <.001 |
|  | 1983-1993; age: 30-39 | 0.40 | 0.21 | (-0.01, 0.81) |  |
|  | 1973-1983; age 40-49 | 0.41 | 0.20 | (0.01, 0.80) |  |
|  | 1963-1973; age 50-59 | 0.68 | 0.19 | (0.30, 1.07) |  |
|  | 1953-1963; age 60-69 | 0.81 | 0.19 | (0.43, 1.19) |  |
|  | 1943 or earlier; age 80+ | 0.91 | 0.22 | (0.48, 1.34) |  |
|  | 1943-1953; age 70-79 | 0.86 | 0.19 | (0.48, 1.24) |  |
| Gender (Ref: Male) | Female | 0.22 | 0.05 | (0.12, 0.33) | <.001 |
|  | Other | -1.02 | 0.39 | (-1.78, -0.27) |  |
| Religious affiliation at age 12 (Ref: No religion/Atheist/Agnostic) | Christianity | 0.47 | 0.12 | (0.24, 0.70) | <.001 |
|  | Judaism | 0.08 | 0.15 | (-0.22, 0.37) |  |
|  | Some other religion | 0.10 | 0.25 | (-0.38, 0.58) |  |
| Race and ethnicity plurality (Ref: Majority) | Minority | 0.35 | 0.06 | (0.22, 0.48) | <.001 |

***Table S20c. Sensitivity to unmeasured confounding of childhood predictors in United States***

| Variable | Category | E-value for Estimate | E-value for 95% CI |
| --- | --- | --- | --- |
| Relationship with mother (Ref: Very bad/Somewhat bad)) | Very good/Somewhat good | 1.49 | 1.14 |
| Relationship with father (Ref: Very bad/Somewhat bad)) | Very good/Somewhat good | 1.50 | 1.22 |
| Parent marital status (Ref: Parents married) | No, divorced | 1.21 | 1.00 |
|  | Never | 1.17 | 1.00 |
|  | No, one or both of them had died | 1.73 | 1.00 |
| Subjective financial status of family growing up (Ref: Got by) | Lived comfortably | 1.29 | 1.04 |
|  | Found it difficult | 1.39 | 1.00 |
|  | Found it very difficult | 1.47 | 1.00 |
| Abuse (Ref: No) | Yes | 1.46 | 1.22 |
| Outsider growing up (Ref: No) | Yes | 1.63 | 1.39 |
| Self-rated health growing up (Ref: Good) | Excellent | 2.32 | 2.06 |
|  | Very good | 1.74 | 1.48 |
|  | Fair | 1.57 | 1.00 |
|  | Poor | 1.39 | 1.00 |
| Immigration status (Ref: Born in this country) | Born in another country | 1.53 | 1.12 |
| Age 12 religious service attendance (Ref: Never) | At least 1/week | 1.42 | 1.07 |
|  | 1-3/month | 1.23 | 1.00 |
|  | <1/month | 1.02 | 1.00 |
| Age group (Ref: 1998-2005; current age: 18-24) | 1993-1998; age: 25-29 | 1.26 | 1.00 |
|  | 1983-1993; age: 30-39 | 1.71 | 1.00 |
|  | 1973-1983; age 40-49 | 1.71 | 1.07 |
|  | 1963-1973; age 50-59 | 2.10 | 1.57 |
|  | 1953-1963; age 60-69 | 2.29 | 1.75 |
|  | 1943 or earlier; age 80+ | 2.44 | 1.82 |
|  | 1943-1953; age 70-79 | 2.37 | 1.82 |
| Gender (Ref: Male) | Female | 1.46 | 1.30 |
|  | Other | 2.62 | 1.53 |
| Religious affiliation at age 12 (Ref: No religion/Atheist/Agnostic) | Christianity | 1.81 | 1.49 |
|  | Some other religion | 1.27 | 1.00 |
| Race and ethnicity plurality (Ref: Majority) | Minority | 1.64 | 1.46 |
|  | Judaism | 1.23 | 1.00 |

***Table S21a. Nationally representative descriptive statistics for Sweden***

| **Characteristic** | **N = 15,068**^1^ |
| --- | --- |
| **Relationship with mother** |  |
| Very good | 8,743 (58%) |
| Somewhat good | 4,513 (30%) |
| Somewhat bad | 1,194 (7.9%) |
| Very bad | 371 (2.5%) |
| Does not apply | 216 (1.4%) |
| Missing | 30 (0.2%) |
| **Relationship with father** |  |
| Very good | 7,134 (47%) |
| Somewhat good | 4,885 (32%) |
| Somewhat bad | 1,588 (11%) |
| Very bad | 725 (4.8%) |
| Does not apply | 720 (4.8%) |
| Missing | 16 (0.1%) |
| **Parent marital status** |  |
| Yes, married | 10,887 (72%) |
| No, divorced | 1,927 (13%) |
| Never married | 1,747 (12%) |
| No, one or both of them had died | 362 (2.4%) |
| Missing | 145 (1.0%) |
| **Subjective financial status of family growing up** |  |
| Lived comfortably | 5,951 (39%) |
| Got by | 7,717 (51%) |
| Found it difficult | 1,238 (8.2%) |
| Found it very difficult | 140 (0.9%) |
| Missing | 22 (0.1%) |
| **Abuse** |  |
| Yes | 2,288 (15%) |
| No | 12,735 (85%) |
| Missing | 45 (0.3%) |
| **Outsider growing up** |  |
| Yes | 1,867 (12%) |
| No | 13,034 (86%) |
| Missing | 168 (1.1%) |
| **Self-rated health growing up** |  |
| Excellent | 5,733 (38%) |
| Very good | 5,124 (34%) |
| Good | 2,669 (18%) |
| Fair | 1,108 (7.4%) |
| Poor | 397 (2.6%) |
| Missing | 38 (0.2%) |
| **Immigration status** |  |
| Born in this country | 13,922 (92%) |
| Born in another country | 1,052 (7.0%) |
| Missing | 94 (0.6%) |
| **Age 12 religious service attendance** |  |
| At least 1/week | 955 (6.3%) |
| 1-3/month | 1,362 (9.0%) |
| <1/month | 6,224 (41%) |
| Never | 6,472 (43%) |
| Missing | 54 (0.4%) |
| **Age group** |  |
| 1998-2005; current age: 18-24 | 1,515 (10%) |
| 1993-1998; age: 25-29 | 1,399 (9.3%) |
| 1983-1993; age: 30-39 | 2,398 (16%) |
| 1973-1983; age 40-49 | 2,221 (15%) |
| 1963-1973; age 50-59 | 2,493 (17%) |
| 1953-1963; age 60-69 | 2,168 (14%) |
| 1943 or earlier; age 80+ | 621 (4.1%) |
| 1943-1953; age 70-79 | 2,253 (15%) |
| Missing | 0 (0%) |
| **Gender** |  |
| Male | 7,536 (50%) |
| Female | 7,493 (50%) |
| Other | 27 (0.2%) |
| Missing | 12 (<0.1%) |
| **Religious affiliation at age 12** |  |
| Baha’i | 3 (<0.1%) |
| Buddhism | 41 (0.3%) |
| Christianity | 10,617 (70%) |
| Confucianism | 4 (<0.1%) |
| Hinduism | 16 (0.1%) |
| Islam | 462 (3.1%) |
| Judaism | 51 (0.3%) |
| No religion/Atheist/Agnostic | 3,738 (25%) |
| Primal, Animist, or Folk religion | 31 (0.2%) |
| Shinto | 1 (<0.1%) |
| Sikhism | 9 (<0.1%) |
| Some other religion | 69 (0.5%) |
| Missing | 26 (0.2%) |
| **Race and ethnicity** |  |
| Missing | 15,068 (100%) |
| ^1^n (%) | |

***Table S21b. Regression of self-rated hope on childhood predictors for Sweden***

| Variable | Category | Estimate | SE | 95% CI | Global p-value |
| --- | --- | --- | --- | --- | --- |
| Relationship with mother (Ref: Very bad/Somewhat bad) | Very good/Somewhat good | 0.02 | 0.09 | (-0.15, 0.19) | 0.938 |
| Relationship with father (Ref: Very bad/Somewhat bad) | Very good/Somewhat good | 0.03 | 0.07 | (-0.11, 0.18) | 0.463 |
| Parent marital status (Ref: Parents married) | No, divorced | 0.20 | 0.07 | (0.05, 0.35) | 0.001 |
|  | Never | 0.00 | 0.08 | (-0.14, 0.15) |  |
|  | No, one or both of them had died | 0.03 | 0.16 | (-0.28, 0.35) |  |
| Subjective financial status of family growing up (Ref: Got by) | Lived comfortably | 0.19 | 0.05 | (0.10, 0.28) | <.001 |
|  | Found it difficult | 0.04 | 0.09 | (-0.14, 0.23) |  |
|  | Found it very difficult | 0.36 | 0.29 | (-0.20, 0.93) |  |
| Abuse (Ref: No) | Yes | -0.15 | 0.07 | (-0.29, -0.01) | <.001 |
| Outsider growing up (Ref: No) | Yes | -0.36 | 0.09 | (-0.53, -0.19) | <.001 |
| Self-rated health growing up (Ref: Good) | Excellent | 1.21 | 0.07 | (1.08, 1.34) | <.001 |
|  | Very good | 0.61 | 0.07 | (0.48, 0.74) |  |
|  | Fair | -0.15 | 0.11 | (-0.37, 0.06) |  |
|  | Poor | -0.54 | 0.20 | (-0.93, -0.14) |  |
| Immigration status (Ref: Born in this country) | Born in another country | 0.29 | 0.09 | (0.10, 0.47) | <.001 |
| Age 12 religious service attendance (Ref: Never) | At least 1/week | 0.36 | 0.09 | (0.17, 0.54) | <.001 |
|  | 1-3/month | -0.04 | 0.08 | (-0.19, 0.11) |  |
|  | <1/month | 0.04 | 0.05 | (-0.05, 0.13) |  |
| Age group (Ref: 1998-2005; current age: 18-24) | 1993-1998; age: 25-29 | 0.14 | 0.10 | (-0.06, 0.33) | 0.986 |
|  | 1983-1993; age: 30-39 | 0.07 | 0.09 | (-0.11, 0.24) |  |
|  | 1973-1983; age 40-49 | 0.04 | 0.09 | (-0.14, 0.21) |  |
|  | 1963-1973; age 50-59 | 0.09 | 0.09 | (-0.08, 0.27) |  |
|  | 1953-1963; age 60-69 | 0.13 | 0.09 | (-0.04, 0.31) |  |
|  | 1943 or earlier; age 80+ | 0.15 | 0.13 | (-0.10, 0.39) |  |
|  | 1943-1953; age 70-79 | 0.12 | 0.09 | (-0.06, 0.30) |  |
| Gender (Ref: Male) | Female | 0.13 | 0.04 | (0.04, 0.21) | <.001 |
|  | Other | -1.73 | 0.54 | (-2.79, -0.66) |  |
| Religious affiliation at age 12 (Ref: No religion/Atheist/Agnostic) | Christianity | 0.26 | 0.05 | (0.15, 0.36) | <.001 |
|  | Some other religion | 0.64 | 0.13 | (0.38, 0.89) |  |

***Table S21c. Sensitivity to unmeasured confounding of childhood predictors in Sweden***

| Variable | Category | E-value for Estimate | E-value for 95% CI |
| --- | --- | --- | --- |
| Relationship with mother (Ref: Very bad/Somewhat bad)) | Very good/Somewhat good | 1.10 | 1.00 |
| Relationship with father (Ref: Very bad/Somewhat bad)) | Very good/Somewhat good | 1.13 | 1.00 |
| Parent marital status (Ref: Parents married) | No, divorced | 1.38 | 1.17 |
|  | Never | 1.04 | 1.00 |
|  | No, one or both of them had died | 1.13 | 1.00 |
| Subjective financial status of family growing up (Ref: Got by) | Lived comfortably | 1.37 | 1.25 |
|  | Found it difficult | 1.15 | 1.00 |
|  | Found it very difficult | 1.58 | 1.00 |
| Abuse (Ref: No) | Yes | 1.31 | 1.05 |
| Outsider growing up (Ref: No) | Yes | 1.58 | 1.37 |
| Self-rated health growing up (Ref: Good) | Excellent | 2.61 | 2.44 |
|  | Very good | 1.86 | 1.71 |
|  | Fair | 1.32 | 1.00 |
|  | Poor | 1.78 | 1.31 |
| Immigration status (Ref: Born in this country) | Born in another country | 1.49 | 1.25 |
| Age 12 religious service attendance (Ref: Never) | At least 1/week | 1.57 | 1.35 |
|  | 1-3/month | 1.15 | 1.00 |
|  | <1/month | 1.14 | 1.00 |
| Age group (Ref: 1998-2005; current age: 18-24) | 1993-1998; age: 25-29 | 1.30 | 1.00 |
|  | 1983-1993; age: 30-39 | 1.20 | 1.00 |
|  | 1973-1983; age 40-49 | 1.14 | 1.00 |
|  | 1963-1973; age 50-59 | 1.23 | 1.00 |
|  | 1953-1963; age 60-69 | 1.29 | 1.00 |
|  | 1943 or earlier; age 80+ | 1.31 | 1.00 |
|  | 1943-1953; age 70-79 | 1.27 | 1.00 |
| Gender (Ref: Male) | Female | 1.28 | 1.15 |
|  | Other | 3.38 | 1.93 |
| Religious affiliation at age 12 (Ref: No religion/Atheist/Agnostic) | Christianity | 1.45 | 1.31 |
|  | Some other religion | 1.89 | 1.60 |

***Table S22a. Nationally representative descriptive statistics for Hong Kong***

| **Characteristic** | **N = 3,012**^1^ |
| --- | --- |
| **Relationship with mother** |  |
| Very good | 1,077 (36%) |
| Somewhat good | 1,164 (39%) |
| Somewhat bad | 293 (9.7%) |
| Very bad | 49 (1.6%) |
| Does not apply | 426 (14%) |
| Missing | 3 (<0.1%) |
| **Relationship with father** |  |
| Very good | 868 (29%) |
| Somewhat good | 1,089 (36%) |
| Somewhat bad | 393 (13%) |
| Very bad | 102 (3.4%) |
| Does not apply | 557 (19%) |
| Missing | 3 (0.1%) |
| **Parent marital status** |  |
| Yes, married | 2,752 (91%) |
| No, divorced | 114 (3.8%) |
| Never married | 40 (1.3%) |
| No, one or both of them had died | 50 (1.7%) |
| Missing | 56 (1.8%) |
| **Subjective financial status of family growing up** |  |
| Lived comfortably | 906 (30%) |
| Got by | 1,527 (51%) |
| Found it difficult | 473 (16%) |
| Found it very difficult | 84 (2.8%) |
| Missing | 22 (0.7%) |
| **Abuse** |  |
| Yes | 318 (11%) |
| No | 2,688 (89%) |
| Missing | 5 (0.2%) |
| **Outsider growing up** |  |
| Yes | 664 (22%) |
| No | 2,224 (74%) |
| Missing | 124 (4.1%) |
| **Self-rated health growing up** |  |
| Excellent | 545 (18%) |
| Very good | 1,073 (36%) |
| Good | 863 (29%) |
| Fair | 426 (14%) |
| Poor | 91 (3.0%) |
| Missing | 13 (0.4%) |
| **Immigration status** |  |
| Born in this country | 2,637 (88%) |
| Born in another country | 321 (11%) |
| Missing | 53 (1.8%) |
| **Age 12 religious service attendance** |  |
| At least 1/week | 432 (14%) |
| 1-3/month | 528 (18%) |
| <1/month | 753 (25%) |
| Never | 1,295 (43%) |
| Missing | 4 (0.1%) |
| **Age group** |  |
| 1998-2005; current age: 18-24 | 217 (7.2%) |
| 1993-1998; age: 25-29 | 198 (6.6%) |
| 1983-1993; age: 30-39 | 507 (17%) |
| 1973-1983; age 40-49 | 580 (19%) |
| 1963-1973; age 50-59 | 711 (24%) |
| 1953-1963; age 60-69 | 620 (21%) |
| 1943 or earlier; age 80+ | 15 (0.5%) |
| 1943-1953; age 70-79 | 164 (5.5%) |
| Missing | 0 (0%) |
| **Gender** |  |
| Male | 1,390 (46%) |
| Female | 1,620 (54%) |
| Other | 2 (<0.1%) |
| Missing | 0 (0%) |
| **Religious affiliation at age 12** |  |
| Buddhism | 323 (11%) |
| Chinese folk/traditional religion | 108 (3.6%) |
| Christianity | 715 (24%) |
| Confucianism | 10 (0.3%) |
| Hinduism | 27 (0.9%) |
| Islam | 86 (2.9%) |
| Jainism | 1 (<0.1%) |
| Judaism | 16 (0.5%) |
| No religion/Atheist/Agnostic | 1,601 (53%) |
| Primal, Animist, or Folk religion | 15 (0.5%) |
| Shinto | 18 (0.6%) |
| Sikhism | 4 (0.1%) |
| Some other religion | 5 (0.2%) |
| Taoism | 81 (2.7%) |
| Missing | 1 (<0.1%) |
| **Race and ethnicity** |  |
| Chinese (Cantonese) | 1,930 (64%) |
| Chinese (Chaoshan) | 201 (6.7%) |
| Chinese (Fujianese) | 117 (3.9%) |
| Chinese (Hakka) | 121 (4.0%) |
| Chinese (Other ethnicity) | 264 (8.8%) |
| Chinese (Shanghainese) | 89 (2.9%) |
| East Asian (Korean, Japanese) | 10 (0.3%) |
| Other | 4 (0.1%) |
| South Asian (Indian, Nepalese, Pakistani) | 17 (0.6%) |
| Southeast Asian (Filipino, Indonesian, Thailand) | 46 (1.5%) |
| Taiwanese | 14 (0.4%) |
| White | 15 (0.5%) |
| Missing | 184 (6.1%) |
| ^1^n (%) | |

***Table S22b. Regression of self-rated hope on childhood predictors for Hong Kong***

| Variable | Category | Estimate | SE | 95% CI | Global p-value |
| --- | --- | --- | --- | --- | --- |
| Relationship with mother (Ref: Very bad/Somewhat bad) | Very good/Somewhat good | 0.45 | 0.14 | (0.18, 0.73) | <.001 |
| Relationship with father (Ref: Very bad/Somewhat bad) | Very good/Somewhat good | 0.11 | 0.13 | (-0.14, 0.36) | 0.035 |
| Parent marital status (Ref: Parents married) | No, divorced | -0.02 | 0.30 | (-0.62, 0.57) | 1 |
|  | Never | 0.10 | 0.42 | (-0.72, 0.93) |  |
|  | No, one or both of them had died | -0.05 | 0.49 | (-1.01, 0.91) |  |
| Subjective financial status of family growing up (Ref: Got by) | Lived comfortably | 0.66 | 0.10 | (0.46, 0.86) | <.001 |
|  | Found it difficult | -0.22 | 0.16 | (-0.53, 0.09) |  |
|  | Found it very difficult | 0.00 | 0.43 | (-0.84, 0.84) |  |
| Abuse (Ref: No) | Yes | -0.06 | 0.15 | (-0.35, 0.24) | 0.961 |
| Outsider growing up (Ref: No) | Yes | -0.02 | 0.13 | (-0.27, 0.23) | 0.996 |
| Self-rated health growing up (Ref: Good) | Excellent | 1.71 | 0.16 | (1.40, 2.03) | <.001 |
|  | Very good | 0.85 | 0.11 | (0.63, 1.08) |  |
|  | Fair | -0.66 | 0.15 | (-0.95, -0.37) |  |
|  | Poor | -1.12 | 0.46 | (-2.01, -0.22) |  |
| Immigration status (Ref: Born in this country) | Born in another country | 0.13 | 0.18 | (-0.23, 0.48) | 0.697 |
| Age 12 religious service attendance (Ref: Never) | At least 1/week | 0.66 | 0.17 | (0.32, 1.00) | <.001 |
|  | 1-3/month | 0.52 | 0.14 | (0.25, 0.78) |  |
|  | <1/month | 0.19 | 0.12 | (-0.03, 0.42) |  |
| Age group (Ref: 1998-2005; current age: 18-24) | 1993-1998; age: 25-29 | -0.02 | 0.21 | (-0.42, 0.39) | <.001 |
|  | 1983-1993; age: 30-39 | 0.13 | 0.15 | (-0.17, 0.42) |  |
|  | 1973-1983; age 40-49 | 0.31 | 0.14 | (0.03, 0.59) |  |
|  | 1963-1973; age 50-59 | 0.57 | 0.14 | (0.29, 0.85) |  |
|  | 1953-1963; age 60-69 | 0.88 | 0.17 | (0.55, 1.20) |  |
|  | 1943 or earlier; age 80+ | 0.47 | 0.26 | (-0.04, 0.99) |  |
|  | 1943-1953; age 70-79 | 1.19 | 0.29 | (0.63, 1.75) |  |
| Gender (Ref: Male) | Female | 0.12 | 0.08 | (-0.04, 0.28) | 0.005 |
|  | Other | -0.47 | 0.29 | (-1.03, 0.09) |  |
| Religious affiliation at age 12 (Ref: No religion/Atheist/Agnostic) | Buddhism | 0.05 | 0.14 | (-0.23, 0.34) | 0.524 |
|  | Chinese folk/traditional religion | 0.24 | 0.23 | (-0.21, 0.68) |  |
|  | Christianity | 0.03 | 0.13 | (-0.23, 0.29) |  |
|  | Some other religion | 0.26 | 0.15 | (-0.04, 0.56) |  |
| Race and ethnicity plurality (Ref: Majority) | Minority | 0.06 | 0.10 | (-0.13, 0.25) | 0.797 |

***Table S22c. Sensitivity to unmeasured confounding of childhood predictors in Hong Kong***

| Variable | Category | E-value for Estimate | E-value for 95% CI |
| --- | --- | --- | --- |
| Relationship with mother (Ref: Very bad/Somewhat bad)) | Very good/Somewhat good | 1.73 | 1.37 |
| Relationship with father (Ref: Very bad/Somewhat bad)) | Very good/Somewhat good | 1.28 | 1.00 |
| Parent marital status (Ref: Parents married) | No, divorced | 1.11 | 1.00 |
|  | Never | 1.26 | 1.00 |
|  | No, one or both of them had died | 1.17 | 1.00 |
| Subjective financial status of family growing up (Ref: Got by) | Lived comfortably | 1.99 | 1.74 |
|  | Found it difficult | 1.43 | 1.00 |
|  | Found it very difficult | 1.02 | 1.00 |
| Abuse (Ref: No) | Yes | 1.19 | 1.00 |
| Outsider growing up (Ref: No) | Yes | 1.10 | 1.00 |
| Self-rated health growing up (Ref: Good) | Excellent | 3.62 | 3.07 |
|  | Very good | 2.25 | 1.96 |
|  | Fair | 2.00 | 1.63 |
|  | Poor | 2.63 | 1.44 |
| Immigration status (Ref: Born in this country) | Born in another country | 1.30 | 1.00 |
| Age 12 religious service attendance (Ref: Never) | At least 1/week | 2.00 | 1.57 |
|  | 1-3/month | 1.81 | 1.47 |
|  | <1/month | 1.40 | 1.00 |
| Age group (Ref: 1998-2005; current age: 18-24) | 1993-1998; age: 25-29 | 1.09 | 1.00 |
|  | 1983-1993; age: 30-39 | 1.30 | 1.00 |
|  | 1973-1983; age 40-49 | 1.55 | 1.14 |
|  | 1963-1973; age 50-59 | 1.87 | 1.52 |
|  | 1953-1963; age 60-69 | 2.28 | 1.85 |
|  | 1943 or earlier; age 80+ | 1.76 | 1.00 |
|  | 1943-1953; age 70-79 | 2.74 | 1.95 |
| Gender (Ref: Male) | Female | 1.29 | 1.00 |
|  | Other | 1.75 | 1.00 |
| Religious affiliation at age 12 (Ref: No religion/Atheist/Agnostic) | Christianity | 1.13 | 1.00 |
|  | Some other religion | 1.48 | 1.00 |
| Race and ethnicity plurality (Ref: Majority) | Minority | 1.19 | 1.00 |
|  | Buddhism | 1.18 | 1.00 |
|  | Chinese folk/traditional religion | 1.45 | 1.00 |
